# Genomic insights into diverse bacterial taxa that degrade extracellular DNA in marine sediments

**DOI:** 10.1038/s41564-021-00917-9

**Published:** 2021-06-14

**Authors:** Kenneth Wasmund, Claus Pelikan, Arno Schintlmeister, Michael Wagner, Margarete Watzka, Andreas Richter, Srijak Bhatnagar, Amy Noel, Casey R. J. Hubert, Thomas Rattei, Thilo Hofmann, Bela Hausmann, Craig W. Herbold, Alexander Loy

**Affiliations:** 1 Division of Microbial Ecology, Centre for Microbiology and Environmental Systems Science, University of Vienna, Vienna, Austria; 2 Austrian Polar Research Institute, Vienna, Austria; 3 Department of Chemistry and Bioscience, Aalborg University, Aalborg, Denmark; 4 Large-Instrument Facility for Environmental and Isotope Mass Spectrometry, Centre for Microbiology and Environmental Systems Science, University of Vienna, Vienna, Austria; 5 Division of Terrestrial Ecosystem Research, Centre for Microbiology and Environmental Systems Science, University of Vienna, Vienna, Austria; 6 Geomicrobiology Group, Department of Biological Sciences, University of Calgary, Calgary, Alberta Canada; 7 Division of Computational Systems Biology, Centre for Microbiology and Environmental Systems Science, University of Vienna, Vienna, Austria; 8 Division of Environmental Geosciences, Centre for Microbiology and Environmental Systems Science, University of Vienna, Vienna, Austria; 9 Joint Microbiome Facility of the Medical University of Vienna and the University of Vienna, Vienna, Austria; 10 Department of Laboratory Medicine, Medical University of Vienna, Vienna, Austria

**Keywords:** Environmental microbiology, Environmental sciences

## Abstract

Extracellular DNA is a major macromolecule in global element cycles, and is a particularly crucial phosphorus, nitrogen and carbon source for microorganisms in the seafloor. Nevertheless, the identities, ecophysiology and genetic features of DNA-foraging microorganisms in marine sediments are largely unknown. Here, we combined microcosm experiments, DNA stable isotope probing (SIP), single-cell SIP using nano-scale secondary isotope mass spectrometry (NanoSIMS) and genome-centric metagenomics to study microbial catabolism of DNA and its subcomponents in marine sediments. ^13^C-DNA added to sediment microcosms was largely degraded within 10 d and mineralized to ^13^CO_2_. SIP probing of DNA revealed diverse ‘*Candidatus* Izemoplasma’, *Lutibacter*, *Shewanella* and Fusibacteraceae incorporated DNA-derived ^13^C-carbon. NanoSIMS confirmed incorporation of ^13^C into individual bacterial cells of Fusibacteraceae sorted from microcosms. Genomes of the ^13^C-labelled taxa all encoded enzymatic repertoires for catabolism of DNA or subcomponents of DNA. Comparative genomics indicated that diverse ‘*Candidatus* Izemoplasmatales’ (former Tenericutes) are exceptional because they encode multiple (up to five) predicted extracellular nucleases and are probably specialized DNA-degraders. Analyses of additional sediment metagenomes revealed extracellular nuclease genes are prevalent among Bacteroidota at diverse sites. Together, our results reveal the identities and functional properties of microorganisms that may contribute to the key ecosystem function of degrading and recycling DNA in the seabed.

## Abstract (web summary)

Using microcosms, stable isotope probing, genome-resolved metagenomics and NanoSIMS, the authors identify diverse bacterial taxa that can degrade extracellular DNA in marine sediments, including ‘*Candidatus* Izemoplasma’, which encode numerous extracellular nucleases.

## Main

Subsurface environments including marine sediments harbour the bulk of microbial biomass on Earth^1^. Nevertheless, the functional capabilities and ecological roles of most of the diversity of subsurface microorganisms remain completely unknown^2^. The vast majority of microorganisms inhabiting marine sediments are ultimately sustained by heterotrophic metabolisms. Heterotrophs are predominantly fuelled by organic matter derived from primary production in the overlying water column and/or from land-derived inputs^3,4^. Additionally, cell debris released after the death and lysis of organisms, as well as fermentation products and excreted exometabolites, supply organic molecules for the growth of microorganisms^5,6^. The identities of microorganisms that use different kinds of organic molecules in marine sediments are, however, largely unknown.

In general, the most abundant of the various classes of organic molecules available for catabolism by microorganisms in marine sediments, and in most other environments, include proteins, lipids, carbohydrates and nucleic acids—the molecules that make up most of the biomass of any cell or virus^7–9^. Of these, DNA is known to contribute substantially to oceanic and sedimentary biogeochemical cycles, acting as an important source of carbon, nitrogen and phosphorus, and providing an energy source^10–12^. Estimates suggest ‘bioavailable’ DNA, that is, DNA available for digestion by extracellular nucleases, supplies microbial communities of coastal and deep-sea sediments with 2−4% of their carbon requirements, 7–4% of their nitrogen requirements and a remarkable 20–47% of their phosphorus requirements^10,12^. Analyses of microbial 16S ribosomal RNA genes derived from extracellular- and intracellular-DNA pools in marine sediments showed that extracellular DNA is rapidly turned over in both shallow and deeper sediments (down to 10–12 m)^13,14^.

Some microorganisms are capable of catabolizing DNA via concerted steps involving extracellular digestion^15^, import systems^16^ and catabolic breakdown of imported subcomponents in the cytoplasm^17^. Degradation products of nucleic acids such as urea and ammonium, as well as CO_2_ and acetate^18^, are also important nutrients for other members of microbial communities. Nucleic acids might be especially important phosphorus sources in sediments rich in metal-oxides such as Fe(III)- or Mn(IV)-oxides, whereby strong sorption of phosphorus to the metal-oxides can occur, thereby diminishing bioavailable pools of this crucial nutrient^19^. Despite the fact that nutrient-rich and ubiquitous DNA molecules are available in marine sediments, our knowledge of microorganisms that degrade and mineralize DNA remains poor^20^.

In this study, we identified microbial players that degrade and catabolise extracellular DNA in marine sediments. To identify and study the ecophysiology of DNA-degrading microorganisms, we performed a functional microbiome approach including marine sediment microcosm experiments supplemented with ^13^C-labelled DNA or individual unlabelled DNA subcomponents, DNA-based SIP (DNA-SIP), single-cell SIP using NanoSIMS, metagenomics and comparative genomics. The combined results provided multiple lines of evidence for the ability to degrade DNA by diverse groups of poorly understood bacteria, thereby highlighting potential niche occupation and in situ functions of these microorganisms in the global seafloor.

## Results

### Microcosm experiments show mineralization of DNA under cold, anoxic conditions

**Extended Data Fig. 1:**
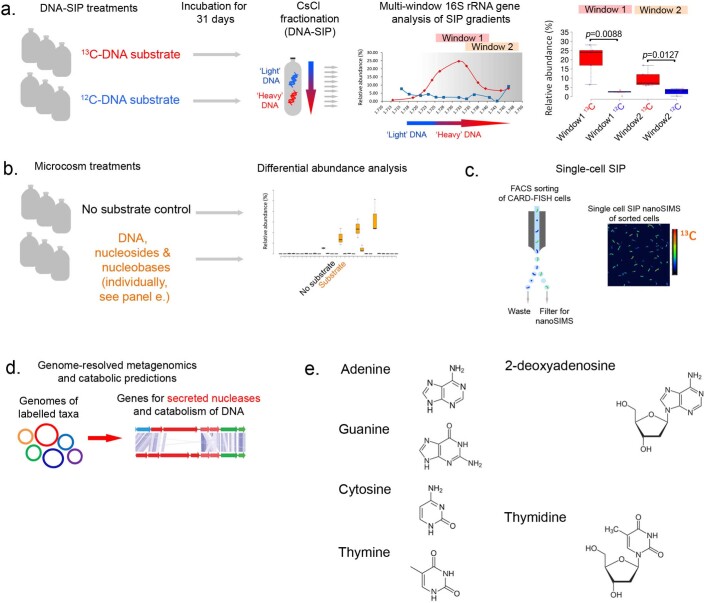
Schematic of study components. **a**, DNA-SIP showing multi-window statistical analyses to identify ^13^C-labelled taxa from gradients. **b**, Shows microcosm set-up and differential abundance analyses. **c**, Cell-sorting of CARD-FISH labelled cells via FACS to concentrate cells for NanoSIMS analysis. **d**, Genome-resolved metagenomic analyses to identify genetic basis for DNA-degrading potential. **e**, Molecular structures of nucleobases and nucleosides supplemented to microcosms (as depicted in panel c.).

**Extended Data Fig. 2:**
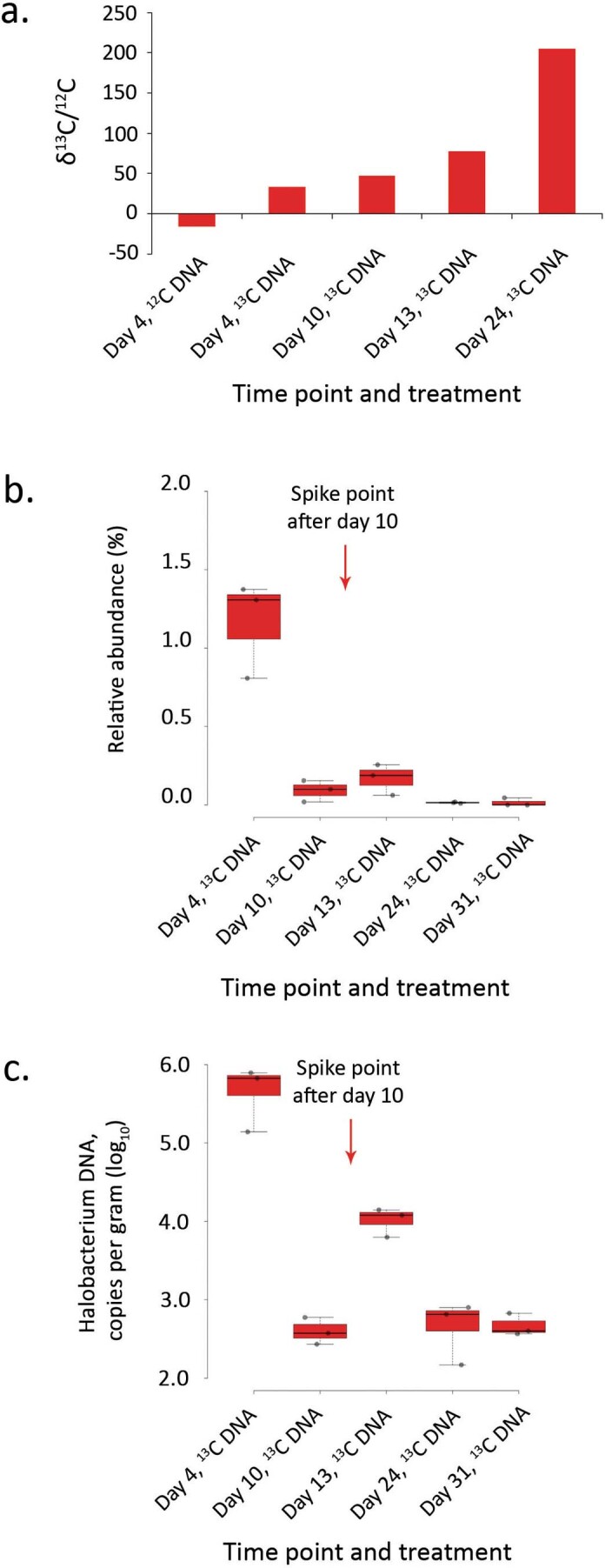
Supplemented archaeal DNA is degraded. **a**, Isotopic composition of CO_2_ from the headspace of a single representative microcosm from each time point. **b**, Relative abundances of 16S rRNA genes of the archaeon *Halobacterium salinarum* among triplicate microcosms (*n* = 3), of which its DNA was added to the microcosms as substrate. **c**, Quantification of *Halobacterium salinarum* DNA as determined by qPCR (*n* = 3 technical replicates) among triplicate microcosms (*n* = 3). It should be noted that relative abundances and qPCR data were not determined at day 0 because the *Halobacterium* DNA was supplemented after sub-sampling. ‘Spike point’ denotes the day at which additional *Halobacterium* DNA was added to the microcosms after sub-sampling (see Materials and Methods). In box plots: center lines show the medians; box limits indicate the 25th and 75th percentiles as determined by R software; whiskers extend 1.5 times the interquartile range from the 25th and 75th percentiles; data points are plotted as open circles. Source data

Anoxic microcosms with slurries of marine sediments from Baffin Bay, Greenland, were individually amended with ^13^C-labelled or unlabelled genomic DNA from the archaeon *Halobacterium salinarum*, as well as the unlabelled nucleobases adenine, thymine, guanine and cytosine, and the nucleosides 2-deoxyadenosine and thymidine (Extended Data Fig. 1). Mineralization of added DNA to CO_2_ was confirmed by isotopic analysis of CO_2_ in the headspace of the microcosms. This showed CO_2_ became enriched in ^13^C already at early time points (4 d), and that ^13^C-CO_2_ was continuously produced over time (Extended Data Fig. 2a). Bacterial community analyses via amplicon sequencing of 16S rRNA genes from the microcosms revealed that the supplemented *Halobacterium* DNA was mostly depleted from approximately 1.3% to 0.1% from day 4 to day 10, respectively, and was only present in minor relative abundances (<0.1%) from day 13 and after (Extended Data Fig. 2b). Quantitative PCR (qPCR) targeting *Halobacterium* DNA also revealed similar trends, that is, a large depletion from day 4 to day 10, and minor amounts remaining after day 13 (Extended Data Fig. 2c).

**Extended Data Fig. 3:**
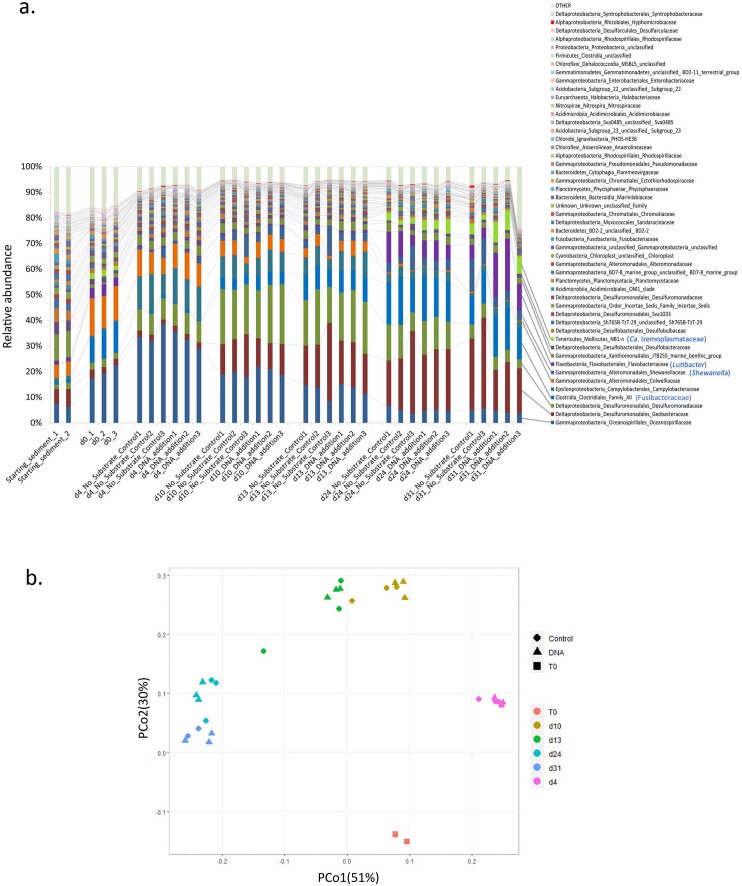
Microbial communities in microcosms. **a**, Major taxa of microbial communities among starting sediments and microcosms throughout incubation. The graph is based on 16S rRNA gene amplicon sequencing data. Relative abundances of family-level classifications are presented, with Phyla_Order_Family’ taxonomic strings presented for all non-proteobacterial lineages, and ‘Class_Order_Family’ taxonomic strings for all proteobacterial lineages. SILVA 16S rRNA taxonomic names are presented for all taxa, and Genome Taxonomy Database (GTDB) taxonomic names for taxa determined to be ^13^C-labelled in SIP (Fig. 1) are given in blue parentheses. The top 50 most abundant families are presented, and all others are combined as ‘Other Families’. Only ^12^C-DNA supplemented treatments and ‘No Substrate Controls’ are shown for clarity. **b**, PCoA plot of microbial community differences among treatments supplemented with DNA versus controls, over time. Source data

Analyses of elements using inductively coupled plasma mass spectrometry showed high concentrations of total manganese and iron that may support the high relative abundances of potential metal-reducing populations that were detected (Supplementary Information). For instance, bacterial taxa related to known metal-reducing microorganisms such as Geobacteraceae and Desulfuromonadaceae, which often dominate in sediments rich in metal-oxides^21–23^, collectively dominated both starting sediments and sediments in microcosms during the incubations (Extended Data Fig. 3a). Bacterial community compositions among microcosms treated with DNA and controls were similar over the time of the experiment (Supplementary Information and Extended Data Fig. 3b). Comparisons of genera present within in situ sediments and microcosms showed that most of the abundant genera present in situ were present in microcosms (Supplementary Table 1 and Supplementary Information).

### DNA-SIP identifies select taxa that incorporate ^13^C-carbon from DNA

**Fig. 1:**
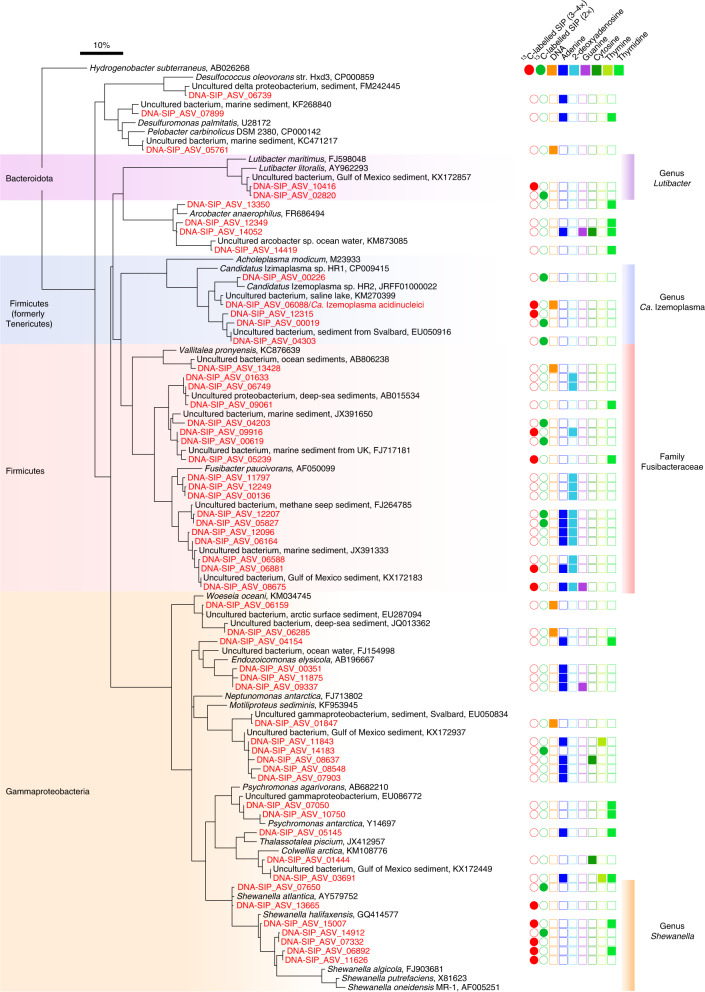
A distinct subset of bacteria use DNA or subcomponents of DNA in sediment microcosms. Phylogeny of 16S rRNA ASVs determined to be enriched in ^13^C in microcosms with ^13^C-DNA as substrate and/or to have significantly increased in relative gene abundance in microcosms with unlabelled DNA or selected DNA subcomponents. ASVs that were determined as ^13^C-labelled by DNA-SIP at 3–4 points (3–4×) are indicated with bright-red-filled circles. ASVs that were determined as ^13^C-labelled by DNA-SIP at two time points (2×) are indicated with green-filled circles. ASVs that increased in relative abundances in microcosms at ≥2 time points in response to additions of DNA, adenine, 2-deoxyadenosine, guanine, cytosine, thymine and thymidine are indicated with filled squares with orange, dark-blue, light-blue, purple, dark-green, green and bright-green colours, respectively. 16S rRNA ASV sequences recovered in this study are highlighted in red. Taxonomic groups that included DNA-degrading bacteria as determined by DNA-SIP analyses are highlighted by shaded colours in the bars. GenBank accessions are included for 16S rRNA gene reference sequences. The scale bar represents 10% sequence divergence. Source data

**Extended Data Fig. 4:**
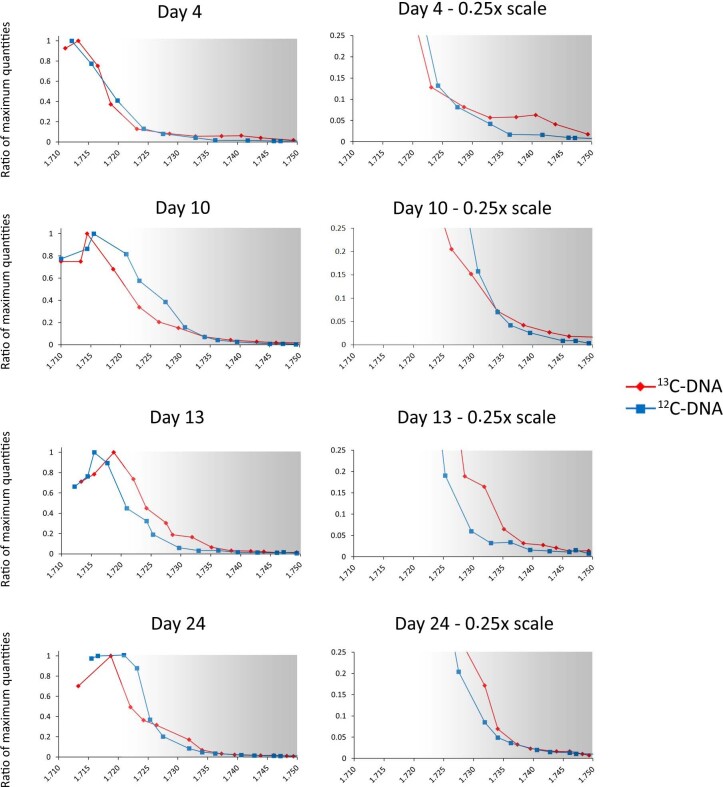
Quantification of total bacterial 16S rRNA genes across buoyant density fractions. Fractions were collected from gradients from ^13^C-DNA and ^12^C-DNA treatments, and quantities of 16S rRNA genes were assessed by qPCR. Values presented are the ratios of maximum quantities detected from each gradient. Grey shaded areas represent ‘heavy’ ends of the gradients. Source data

**Extended Data Fig. 5:**
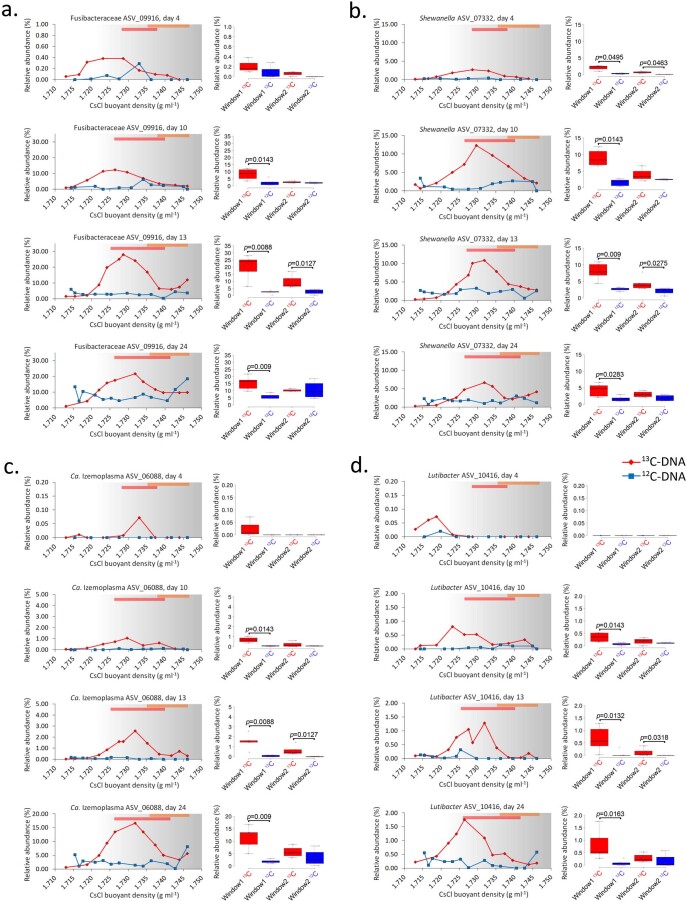
DNA-SIP reveals ^13^C-carbon uptake from ^13^C-DNA by specific taxa. Line plots and box plots of relative abundances of 16S rRNA gene ASVs determined to be enriched in ^13^C by amplicon sequencing across density gradients. **a**, Fusibacteraceae ASV_09916 **b**, *Shewanella* ASV_07332 **c**, *Ca*. Izemoplasma ASV_06088 **d**, *Lutibacter* ASV_10416. ASVs of the most abundant of the four taxa determined to be ^13^C-labelled at 3 or more time points are presented, while other are listed in Fig. 1. Relative abundances determined across ^13^C and ^12^C gradients are plotted in red diamonds and blue squares, respectively. Grey shaded areas indicate the ‘heavy’ ends of the gradients. The red and orange shaded horizontal bars indicate fractions included in Window 1 and Window 2 for statistical comparisons of relative abundances of taxa between ^13^C and ^12^C gradients (Supplementary Table 3). The accompanying box plots show the comparisons of relative abundances from between ^13^C and ^12^C gradients from both Window 1 and Window 2. In the box plots: the centre lines show medians; box limits indicate the 25th and 75th percentiles; whiskers extend 1.5 times the interquartile range from the 25th and 75th percentiles. *p* values and bars are presented where significant differences between relative abundances between ^13^C and ^12^C gradients were obtained using the Wilcoxon Signed Rank Sum Test within Rhea (Lagkouvardos *et al*. 2017).

**Extended Data Fig. 6:**
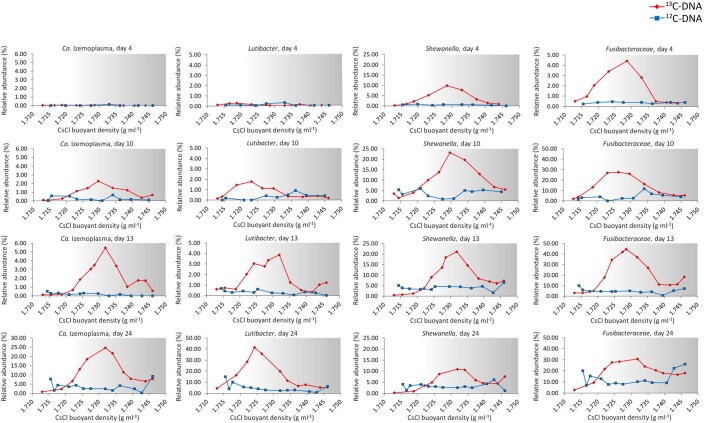
Summed relative abundances of ASVs in taxonomic groups determined to be enriched in ^13^C by amplicon sequencing of 16S rRNA genes across density gradients. Relative abundances determined across ^13^C and ^12^C gradients are plotted in red diamonds and blue squares, respectively. Grey shaded areas indicate the ‘heavy’ ends of the gradients. Source data

**Extended Data Fig. 7:**
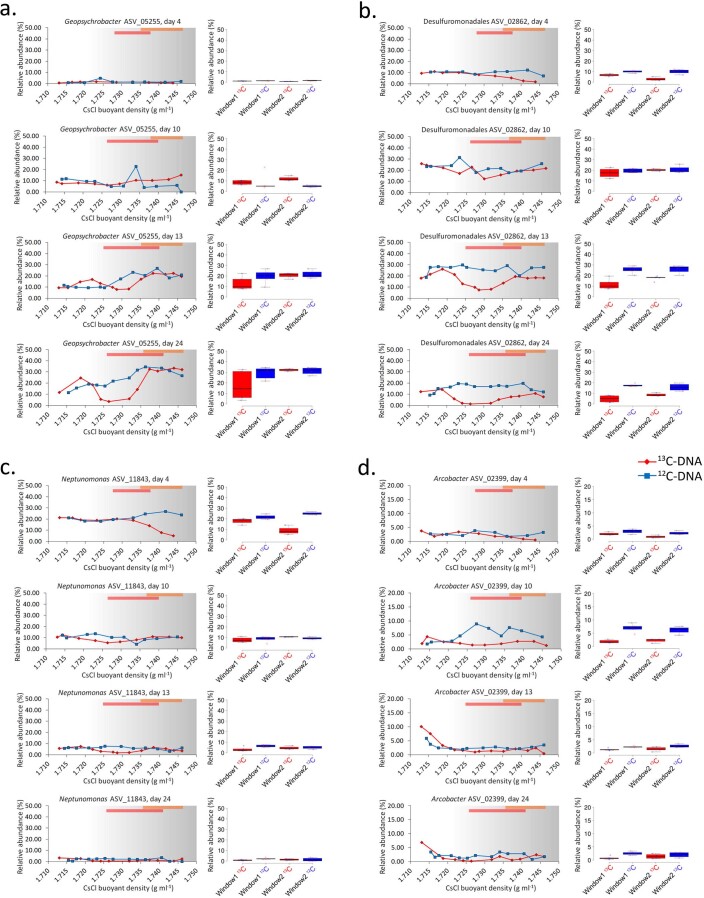
DNA-SIP gradients show unlabelled taxa have similar relative abundances across gradients. Line plots and box plots of relative abundances of the four most abundant 16S rRNA gene ASVs (on average in microcosms) across density gradient fractions. **a**. *Geopsychrobacter* ASV_05255 **b**. Desulfuromonadales ASV_02862 **c**. *Neptunomonas* ASV_11843 **d**. *Arcobacter* ASV_02399. Relative abundances determined across ^13^C and ^12^C gradients are plotted in red diamonds and blue squares, respectively. Grey shaded areas indicate the ‘heavy’ ends of the gradients. The red and orange shaded horizontal bars indicate fractions included in Window 1 and Window 2 for statistical comparisons of relative abundances of taxa between ^13^C and ^12^C gradients (Supplementary Table 2). The accompanying box plots show the comparisons of relative abundances of 16S rRNA genes from between ^13^C- and ^12^C-treatment DNA-SIP gradients, from both Window 1 and Window 2 of the gradients. The values in the box plots are derived from DNA-SIP gradients that were each performed one time, using DNA pooled from the triplicate microcosms. In the box plots: the centre lines show medians; box limits indicate the 25th and 75th percentiles; whiskers extend 1.5 times the interquartile range from the 25th and 75th percentiles.

DNA-SIP identified bacterial taxa that incorporated ^13^C-carbon from the added ^13^C-DNA during replication and growth, and therefore must have: (1) catabolised the added DNA and/or subcomponents of DNA; (2) salvaged nucleobases or nucleosides for incorporation during replication; or (3) utilized fermentation products released from catabolism of the added DNA. Numbers of bacterial 16S rRNA genes determined from qPCR analyses across all gradient density fractions revealed slightly higher abundances of 16S rRNA genes in some ‘heavy’ fractions (≥1.725 g ml^−1^)^24^ from incubations with ^13^C-DNA compared with ^12^C-DNA, from several time points, that is, days 4, 10, 13 and 24 (Extended Data Fig. 4). Although these qPCR results did not indicate large amounts of ^13^C-carbon incorporation into the overall community DNA, ^13^C-carbon incorporation into DNA of specific bacterial taxa was revealed by their increases in relative abundances across the heavy density fractions from ^13^C-DNA treatments. To reveal this, amplicon sequencing of 16S rRNA genes was performed across fractions from various CsCl density gradients (Supplementary Table 2). We identified individual amplicon sequence variants (ASVs) that displayed significantly increased relative abundances in multiple windows of heavy density fractions (>1.725 g ml^−1^) from ^13^C-DNA treatments versus similar densities from ^12^C-DNA control treatments (Extended Data Fig. 1). This identified individual ASVs (*n* = 12) that were significantly enriched at three or more time points (Supplementary Table 3), and these belonged to four bacterial groups (Fig. 1 and Extended Data Fig. 5). Fusibacteraceae ASV_09916 reached the highest relative abundances in heavy densities at any time point, with up to 28% relative abundance in heavy densities around 1.729 g ml^−1^ in gradients from ^13^C-DNA treatments at day 13, versus 3% relative abundance in similar densities in control gradients from ^12^C-DNA treatments (Extended Data Fig. 5). The summed relative abundances of all ASVs belonging to these four ^13^C-labelled taxa also collectively showed large increases in relative abundances in heavy fractions of gradients from ^13^C-DNA treatments versus ^12^C-DNA treatments (Extended Data Fig. 6). This was in stark contrast to ASVs of other abundant taxa that were determined as unlabelled, which showed similar relative abundances across heavy fractions from ^13^C-DNA treatments and ^12^C-DNA control gradients (Extended Data Fig. 7).

**Extended Data Fig. 8:**
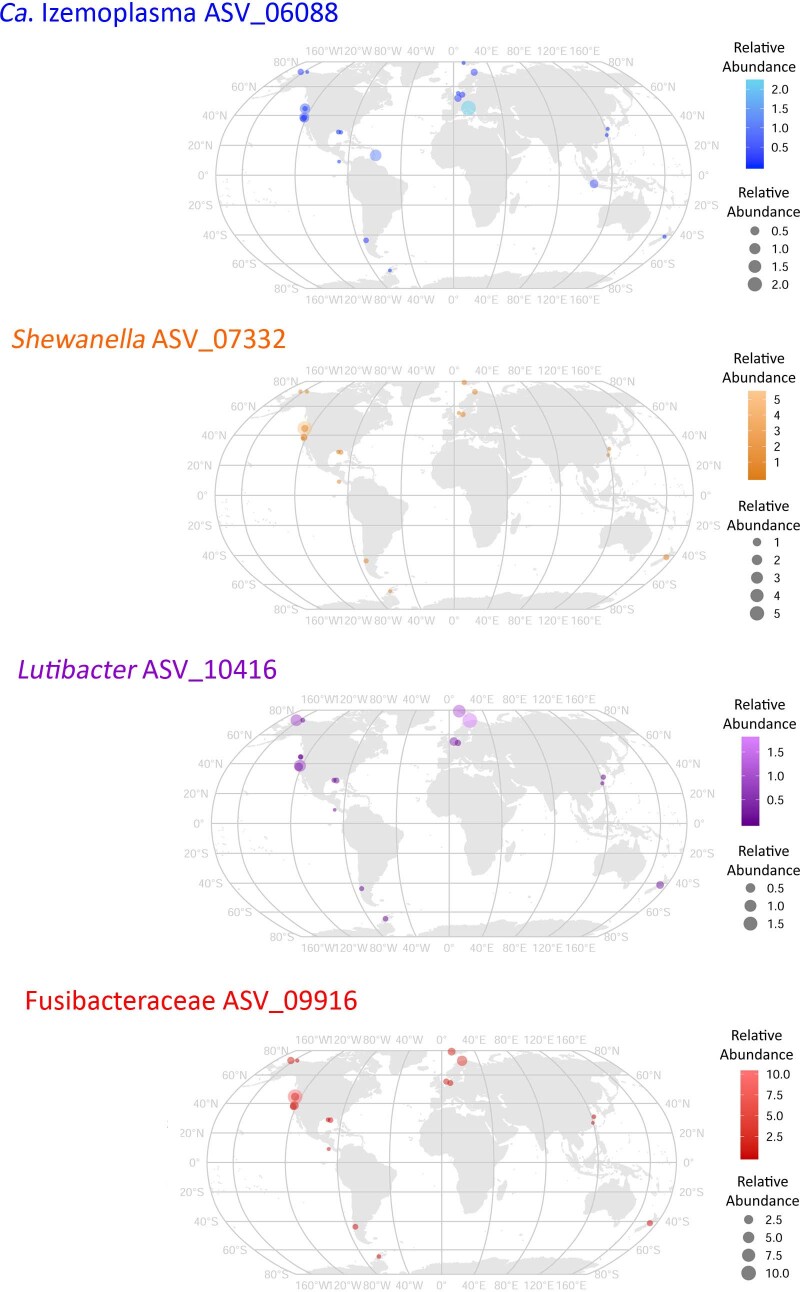
Global presence and relative abundances of 16S rRNA gene sequences related to the top ASVs identified as ^13^C-labelled from the DNA-SIP analysis. 16S rRNA gene sequences were analysed from publicly available Short Read Archive (SRA) datasets via the IMNGS server (see Materials and Methods). In total 247 SRA sampples from marine sediments that contained sequences from all four taxa were identified, and the relative abundances of these taxa in representative samples from each site are presented (*n* = 18). Five additional samples that contained high abundances of *Ca*. Izemoplasma sequences were also plotted. Sequence identity cut-offs of ≥95% were used for *Ca*. Izemoplasma, *Shewanella* and Fusibacteraceae, while ≥97% was used for *Lutibacter*, when querying the SRAs via IMNGS. These cut-offs were applied to reflect the phylogenetic breadth of the labelling among these taxa as determined by DNA-SIP. The size and shading of the circles on the maps correspond to the relative abundances of the specific taxa in each sample, and the scales are provided in the corresponding legends of each map. Source data

Phylogenetic analyses of 16S rRNA genes from the labelled ASVs showed that they were affiliated with: (1) the genus *Shewanella* (class Gammaproteobacteria); (2) the family Fusibacteraceae (phylum Firmicutes); (3) the genus ‘*Candidatus* Izemoplasma’ (Genome Taxonomy Database (GTDB) phylum Firmicutes, formerly phylum Tenericutes); and (4) the genus *Lutibacter* (GTDB phylum Bacteroidota, formerly phylum Bacteroidetes) (Fig. 1). Examination of 16S rRNA gene sequences in publicly available sequence databases showed that closely related sequences from the most strongly ^13^C-labelled ASVs of these groups are globally distributed in coastal and deep-sea sediments (Supplementary Information and Extended Data Fig. 8).

A potential limitation of our DNA-SIP analysis was that detection of ^13^C-labelling of DNA from bacteria with low genomic GC-contents may have been reduced because gradient densities <1.713 g ml^−1^, where low GC-content DNA is expected to accumulate^25^, were not recovered. Nevertheless, we detected clear ^13^C-labelling across the DNA-SIP gradients from taxa of the Fusibacteraceae, *Lutibacter* and *Ca*. Izemoplasma that have very low GC-contents, that is, 30–33%, suggesting this was not the case (further discussed in the Supplementary Information).

### Single-cell SIP confirms ^13^C-carbon uptake from ^13^C-DNA into individual cells

**Fig. 2:**
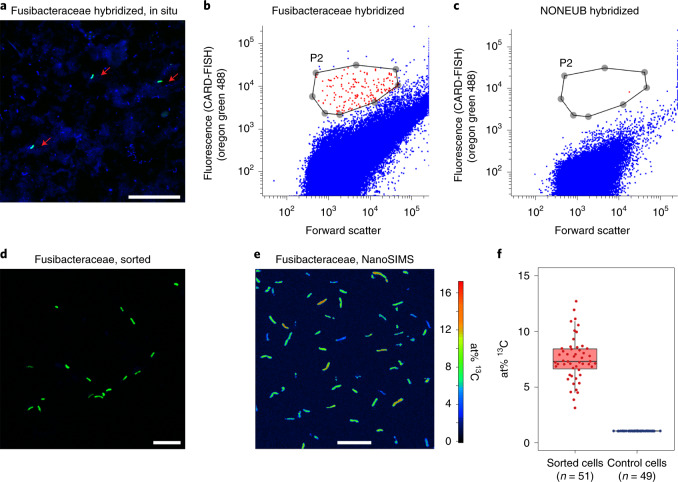
Direct evidence for uptake of ^13^C-carbon from ^13^C-DNA into single cells of bacteria. **a**, Representative CARD-FISH image of Fusibacteraceae in situ with probe Fusi-06 (green are CARD-FISH-hybridized cells; blue are cells counterstained by DAPI; red arrows indicate fluorescently labelled cells conferred by CARD-FISH). Scale bar, 25 μm. **b**, Representative FACS dot-plot showing Fusibacteraceae CARD-FISH-hybridized cells. The sorting gate is shown as grey circles and joining lines. **c**, Representative FACS dot-plot showing NONEUB CARD-FISH-hybridized controls. The sorting gate is shown as grey circles and joining lines. **d**, Representative CARD-FISH image of Fusibacteraceae cells after sorting (green are CARD-FISH-hybridized cells; blue are cells counterstained by DAPI). Scale bar, 10 μm. **e**, NanoSIMS visualization of the ^13^C isotope content (at% ^13^C) of sorted Fusibacteraceae cells after ^13^C-DNA incubation. Scale bar, 10 μm. **f**, ^13^C/(^12^C + ^13^C) isotope fraction (at% ^13^C) values of sorted cells and isotopically unlabelled bacterial cells obtained from region-of-interest-specific NanoSIMS image data evaluation, revealing significant ^13^C isotope enrichment (*P* < 0.001) of Fusibacteraceae cells (Methods). Data points refer to single-cell values; *n* refers to the number of analysed cells. Box plots display the summary statistics where boxes indicate the interquartile ranges (IQR), whiskers show the range of values that are within 1.5 × IQR and horizontal lines indicate the medians. CARD-FISH (**a**) and FACS (**d**) experiments targeting Fusibacteraceae cells were performed multiple times (*n* = 6), while NanoSIMS imaging (**e**) was performed once. Source data

We also confirmed incorporation of ^13^C-carbon into individual bacterial cells using NanoSIMS. For this, we specifically targeted cells of Fusibacteraceae sampled from microcosms from days 10 and 13, with catalysed reporter deposition fluorescence in situ hybridization (CARD-FISH) (Fig. 2a). We then sorted fluorescently labelled cells by fluorescence-activated cell sorting (FACS) to specifically select the target cells for NanoSIMS (Fig. 2b–d). NanoSIMS revealed significant (*P* ≤ 0.001) enhancement of the ^13^C content (7.5 ± 1.9 atom% (at%) ^13^C) in numerous cells (*n* = 51) relative to control cells with natural isotopic composition (1.08 ± 0.008 at% ^13^C) (Fig. 2e,f). This therefore provided unequivocal evidence for incorporation of ^13^C-carbon from ^13^C-DNA into Fusibacteraceae cells. Importantly, this validates ^13^C incorporation by Fusibacteraceae determined by DNA-SIP, and therefore validates ^13^C-labelling of the other taxa that we also determined as ^13^C-labelled from DNA-SIP, that is, the *Ca*. Izemoplasma, *Lutibacter* and *Shewanella*. This is because they showed increased relative abundances in gradients from ^13^C-DNA versus ^12^C-DNA treatments similarly to the Fusibacteraceae, and passed the same strict statistical analyses for determining ^13^C-labelling by DNA-SIP (Extended Data Fig. 5).

### Distinct responses of diverse bacteria to additions of DNA, nucleobases or nucleosides

Complementary to the DNA-SIP analyses, we also tracked the relative abundances of individual ASVs in response to additions of DNA, as well as additions of individual subcomponents of DNA (Fig. 1 and Supplementary Data 1), that is, the nucleobases adenine, thymine, guanine and cytosine, and the nucleosides 2-deoxyadenosine and thymidine (Supplementary Data 1). Only ASVs that were significantly enriched at ≥2 time points, for each treatment, were considered further. This was performed to obtain indications for growth in response to the addition of these substrates. Among the ASVs that were identified as incorporating ^13^C-carbon from ^13^C-DNA by SIP analyses, *Ca*. Izemoplasma ASV_06088 showed significant increases in relative abundances in microcosms with DNA additions versus no-substrate controls, over multiple time points (Supplementary Data 1). Although enriched at only one time point, the *Lutibacter* ASV_02820, which was determined as ^13^C-labelled by DNA-SIP, was notable because it was highly enriched in the microcosms with DNA additions at day 31 versus controls, that is, 15.2% versus 4.4% (*P* = 0.0495) (Supplementary Data 1). For nucleobases, only five ASVs responded to the pyrimidines thymine or cytosine, while 18 ASVs responded to the purines adenine and/or guanine (Fig. 1). Numerous Fusibacteraceae ASVs (*n* = 13) were enriched in response to purine-based nucleobases and/or nucleosides, and particularly those containing adenine moieties (Fig. 1 and Supplementary Data 1). Higher responses were also typically obtained from nucleosides than nucleobases (Fig. 1, Supplementary Data 1 and Supplementary Information).

### Recovery of genomes of DNA-degrading taxa

**Fig. 3:**
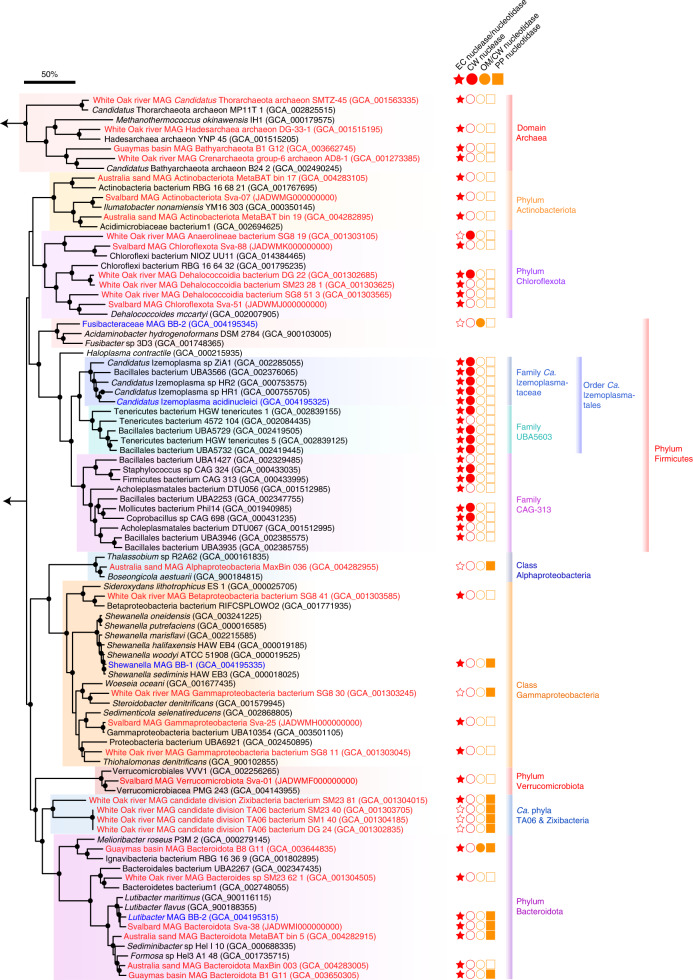
Metagenomics identifies diverse DNA-degrading taxa among distinct sediment sites. Genome-based phylogeny of MAGs that represent taxa determined to be ^13^C-labelled in our SIP experiments and MAGs representing microorganisms with potential for DNA degradation identified from additional publicly available metagenomes. MAGs recovered in this study are highlighted in blue. MAGs from public metagenomes with potential for DNA degradation are highlighted in red. Reference genomes for phylogenetic comparisons are labelled in black. The shaded bars delineate taxonomic groups. Key annotations related to DNA catabolism from all highlighted taxa and key Firmicute bacteria are presented in Supplementary Table 5. Red-filled stars indicate genes for extracellular (EC) nucleases or nucleotidases; red-filled circles indicate genes for cell-wall-bound (CW) nucleases; orange-filled circles indicate genes for outer-membrane-bound (OM) nucleotidases; orange-filled squares indicate genes for periplasmic (PP) nucleotidases. Arrows indicate branches are connected. Bootstrap values >90% are presented as black-filled circles on nodes. The scale bar represents 50% sequence divergence. Source data

**Fig. 4:**
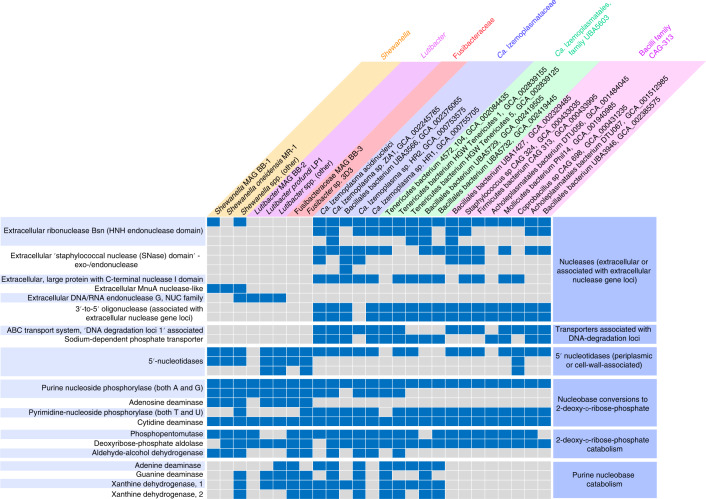
Key genes for DNA-degrading enzymes are encoded among DNA-degraders identified by DNA-SIP. Presence–absence of key genes for enzymes involved in DNA degradation, transport and catabolism of DNA subcomponents in MAGs and genomes of selected isolates. The presence of genes encoding the enzymes is indicated by filled blue boxes. Key annotations from MAGs identified in this study are listed in Supplementary Table 5.

Near complete (>90%) metagenome-assembled genomes (MAG) that were representative of the taxa labelled in the DNA-SIP experiments were recovered (Supplementary Table 4). These MAGs, together with other publicly available genomes and MAGs from related bacteria^19,26–29^, were analysed by phylogenomics (Fig. 3), and for genes encoding enzymes required to catabolise DNA (Fig. 4 and Supplementary Table 5). The *Ca*. Izemoplasma MAG contained a 16S rRNA gene sequence that was 100% identical to ASV_06088, which was labelled in the DNA-SIP experiment. All other MAGs recovered here did not contain 16S rRNA gene sequences. Phylogenomic analyses (Fig. 3) and average nucleotide identity (ANI) analyses (Supplementary Table 4) revealed that all MAGs constituted new species or genera within their respective phylogenetic groups. For the *Ca*. Izemoplasma MAG, we propose the species name *Ca*. Izemoplasma acidinucleici (Supplementary Information).

### Extracellular nucleases and catabolic enzymes for DNA subcomponents encoded in DNA-degraders confer capacity to use environmental DNA

**Fig. 5:**
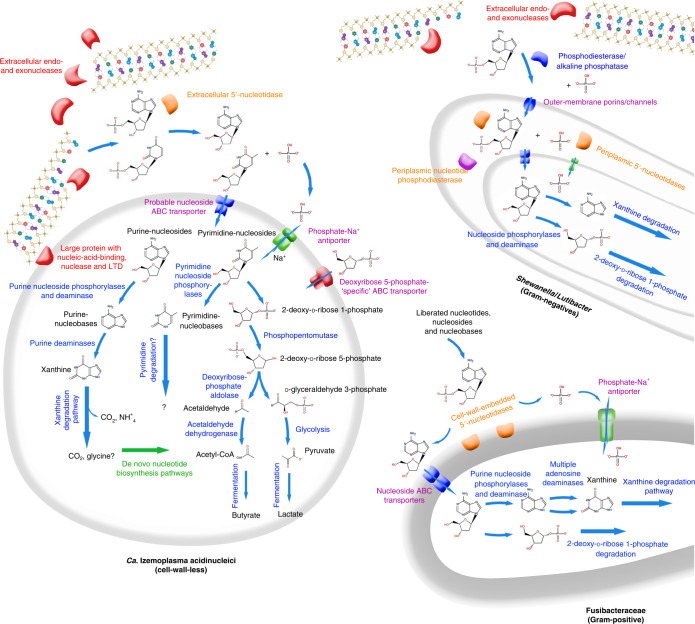
Taxon-centric schematic depiction of key enzymes and other functional proteins related to DNA catabolism and transport annotated from MAGs and close relatives. More detailed pathways for catabolism of DNA subcomponents are provided for *Ca*. Izemoplasma acidinucleici, and, for clarity, simplified versions of these redundant pathways are provided as arrows for the other organisms. LTD, lamin tail domain. DNA-subcomponent molecule structures are sourced from MetaCyc^84^. DNA molecules were created with BioRender.com. Large, black, bold fonts indicate taxa; small black fonts indicate molecules; blue fonts indicate enzymes or enzymatic pathways (as also indicated by corresponding blue arrows); red fonts indicate extracellular nucleases and/or associated proteins; orange fonts indicate secreted nucleotidases; magenta fonts indicate transporters; green fonts indicate biosynthetic pathways.

A key determinant for the ability to utilize DNA as a nutrient and/or energy source is the ability for extracellular digestion of DNA polymers. Importantly, extracellular nucleases can also be differentiated from nucleases used for recycling nucleic acids in the cytoplasm^15^. Accordingly, we identified genes for extracellular nucleases in MAGs and/or related reference genomes of *Shewanella*, *Lutibacter* and *Ca*. Izemoplasma populations (Figs. 4 and 5). We did not, however, find any genes related to extracellular nucleases in the *Fusibacteraceae* MAG BB-3 or reference genomes. Importantly, none of the other MAGs that we obtained and that represented unlabelled taxa encoded extracellular nucleases or nucleotidases. Strikingly, up to five extracellular nucleases were encoded in some of the marine *Ca*. Izemoplasmataceae (Fig. 4). Further, related MAGs from an undescribed *Ca*. Izemoplasmatales family, UBA5603, and a related yet undescribed *Bacilli* family, CAG-313 (Fig. 3), also harboured numerous copies of extracellular nuclease genes (Fig. 4). Encoded extracellular nucleases identified in our *Shewanella* MAG BB-2 are homologs to extracellular nucleases previously shown to be highly expressed when *Shewanella oneidensis* MR-1 was grown on DNA^19,30^.

**Fig. 6:**
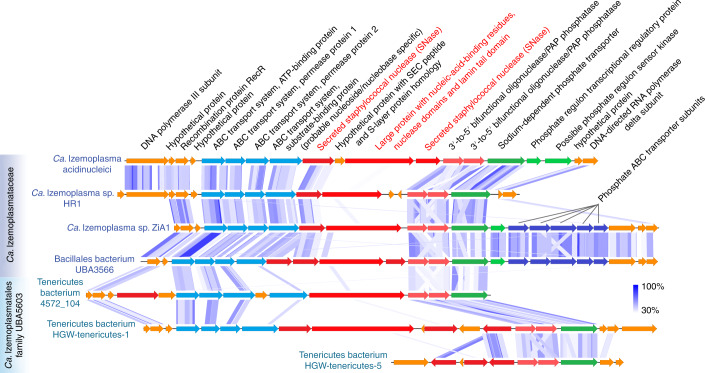
Putative ‘DNA-degradation loci’ in *Ca*. Izemoplasmatales MAGs reveal colocalization of functionally related DNA-degrading genes. Coloured arrows represent genes. Shaded blue lines between genes show regions with high sequence similarity and synteny as determined by tBLASTx using EasyFig. The shaded blue colour in the legend indicates the degree of amino acid percentage identity calculated between protein sequences translated from genes by tBLASTx. DNA-binding domains were predicted based on NCBI Conserved Domains (via BLASTP). Proteins predicted to be secreted are highlighted and labelled in red. Only scaffolds from MAGs with all or most genes present are included. Grey lines point to genes (dark-blue) for subunits of a ‘Phosphate ABC transporter’, which correspond to (from left to right): phosphate-binding subunit; permease protein 1; permease protein 2; ATP-binding protein; regulatory protein.

To facilitate the import of DNA subcomponents into cells, further deconstruction of nucleotides and nucleosides is necessary^31^. MAGs from the *Ca*. Izemoplasmataceae and *Ca*. Izemoplasmatales family UBA5603 harboured conspicuous genomic loci associated with multiple extracellular nuclease genes that encoded enzymes that may facilitate further digestion and import of nucleic acid components, that is, additional 3ʹ–5ʹ nucleases, phosphohydrolases, ABC-transporters and phosphate transporters (Fig. 6). Sequence homology searches of the ‘substrate-binding subunit’ of the ABC-transporters against the IMG database^32^ identified that the genomes of organisms with best hits also encoded nucleases or nucleosidases within the same genomic neighbourhood. This indicated that these are probable nucleoside or nucleobase transporters. Additionally, these loci encoded a large protein (1,049 amino acids) (Fig. 6) that was noteworthy because it contained nucleic-acid-binding, nuclease and ‘lamin tail’ domains (Extended Data Fig. 1 and Supplementary results and discussion).

The *Shewanella* MAG BB-2 recovered here encoded several nucleotidases previously shown to be critical for growth on extracellular DNA^19^, that is, excreted bifunctional 2ʹ,3ʹ cyclic nucleotide 2ʹ phosphodiesterase/3ʹ nucleotidases and a putative UDP-sugar hydrolase protein with 5ʹ nucleotidase domains (included under nucleotidases in Fig. 4). *Lutibacter* and *Shewanella* also harboured multiple copies of genes for 5ʹ-nucleotidases that were predicted to reside in their periplasms (Figs. 4 and 5). Similarly, *Fusibacteraceae* and *Ca*. Izemoplasmataceae genomes encoded 5ʹ-nucleotidases that were predicted to be located in their cell walls or extracellularly, respectively (Figs. 4 and 5). Dephosphorylation of nucleotides by nucleotidases is critical because cell membranes are impermeable to nucleotides due to negatively charged phosphate groups^33,34^.

**Extended Data Fig. 9:**
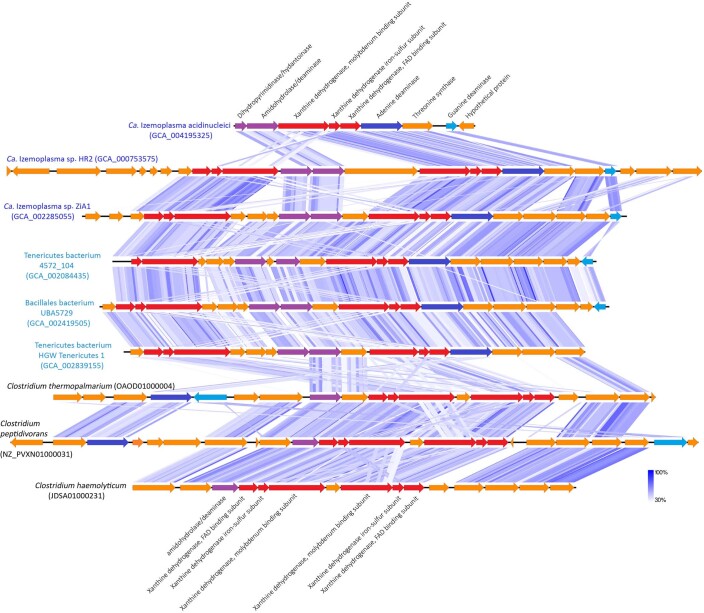
Schematic of gene arrangements of ‘xanthine-degradation loci’ in *Ca*. Izemoplasmatales MAGs and selected *Clostridium* spp. genomes. Shaded blue lines show regions with high sequence similarity and synteny as determined by tBLASTx using EasyFig. Genbank accession numbers are provided in parenthesis.

Once imported into the cytoplasm, purine- and pyrimidine-based deoxyribonucleosides may be further broken down, whereby the respective bases may then enter catabolic pathways or salvage pathways used for incorporation into newly synthesized nucleic acids. All DNA-degraders identified in this study have enzymes required to cleave nucleobases into the sugar and base components. We identified genes for ‘purine deoxyribonucleoside phosphorylases’ in most genomes of putative DNA-degraders (Fig. 4), while genes for ‘pyrimidine deoxyribonucleoside phosphorylases’ were also detected in most *Ca*. Izemoplasmataceae genomes and in the *Fusibacter* sp. 3D3 reference genome (Fig. 4). The *Fusibacter* sp. 3D3 reference genome harboured multiple copies of adenosine deaminase genes, indicating enhanced capabilities to use these molecules (Fig. 4). Genes for deaminases specific for both purine bases were present in *Fusibacteraceae* and various *Ca*. Izemoplasmataceae genomes (Fig. 4), while they were more sparsely identified among the other genomes (Fig. 4). In MAGs of the marine *Ca*. Izemoplasmataceae and the sister family UBA5603 (order *Ca*. Izemoplasmatales), genes for the two purine-specific deaminases were often located in close vicinity to each other, and were distinctively located in the same genomic region as genes for xanthine dehydrogenases and their accessory proteins (Extended Data Fig. 9). Xanthine dehydrogenases are key enzymes required for conversions of purine bases to xanthine, which is a common intermediate of purine breakdown^17^. These genomic loci were highly similar in *Ca*. Izemoplasmatales and the genomes of some *Clostridium* species (Extended Data Fig. 9), which are among the few biochemically characterized anaerobic purine-degraders^17,35^. Apart from indications for the dephosphorylation of pyrimidines described above, we were unable to identify genes for anaerobic catabolic degradation of pyrimidine bases. This was not possible because the enzymes involved in this process in anaerobic microorganisms are unknown. All MAGs representative of taxa that became labelled in our SIP experiment also had the potential to catabolise the 2-deoxy-d-ribose-phosphate sugar moiety (Fig. 4).

### De novo biosynthetic pathways for nucleotides encoded in DNA-degrading taxa

We identified large complements of genes encoding enzymes involved in the complete de novo synthesis of nucleotides in the genomes of most *Ca*. Izemoplasma (Supplementary Table 6 and Supplementary Information). All other DNA-degraders identified in this study belong to bacterial groups that do not require supplementation of nucleic acid building blocks to growth media and therefore have capacity to synthesize their own nucleotides^28,36–38^.

### Metagenomic screening shows specific DNA-degrading taxa are prevalent among additional sediment sites

Different DNA-degrading microorganisms may exist in marine sediment sites and/or depths with other physical and geochemical properties than those sediments studied here. We therefore searched MAGs from other sediment sites for potential DNA-degrading microorganisms. Because our metagenomic results showed secreted nucleases were only encoded by DNA-degrading taxa determined by DNA-SIP, we propose they can be used as biomarkers for DNA-degrading potential. We thus searched predicted secreted nucleases among 205 MAGs obtained from Svalbard fjord sediments (*n* = 97)^39,40^, permeable sandy sediments from Australia (*n* = 12)^41^, non-hydrothermal ‘control’ sediments from the Gulf of Mexico (*n* = 17)^42^ and White Oak River sediments from the USA (*n* = 79)^43^. Notably, six of the seven Bacteroidota MAGs, which were recovered from all four additional metagenomes, encoded both extracellular and periplasmic nucleases or nucleotidases (Fig. 3 and Supplementary Table 5). Further, various MAGs from the Chloroflexota, Actinobacteriota, Gammaproteobacteria, Verrucomicrobiota and Archaea encoded extracellular nucleases (Fig. 3 and Supplementary Table 5). Two of these White Oak River archaeal MAGs were affiliated with the Bathyarchaeota, while others belonged to Thorarchaeota and Hadesarchaeota. In addition, candidate phylum TA06 (*n* = 4) MAGs from White Oak River sediments harboured genes for predicted periplasmic nucleotidases (Fig. 3 and Supplementary Table 5), while one of these TA06 MAGs also encoded a predicted extracellular nuclease. Overall, these results highlight Bacteroidota as widespread DNA-degrading taxa, and further indicate a distinct subset of taxa degrade DNA in marine sediments.

## Discussion

This study revealed the identities of microbial players involved in the turn-over and recycling of extracellular DNA in anoxic marine sediments using culture-independent approaches. DNA-SIP showed a distinct subset of the community became ^13^C-labelled from the added ^13^C-DNA, revealing that only select members of the community have the functional capability to degrade DNA. The ^13^C-enrichment was detectable within 4 d of incubation in some taxa, and they appeared to become further enriched over time. Combined with the complete oxidation of the ^13^C-DNA to ^13^C-CO_2_, this therefore showed relatively fast, as well as ongoing, mineralization of DNA under the cold, anoxic in situ-like conditions. The single-cell analyses by NanoSIMS also confirmed the DNA-SIP results and showed clear enrichment of ^13^C-carbon in Fusibacteraceae cells. Because CARD-FISH causes drastic losses in the relative ^13^C content of treated cells (the degree of label dilution has been reported to be in the range from >25% up to 80%)^44–46^, the average cellular ^13^C content of 7.5 at% strongly suggests that the cells utilized a substantial proportion of the DNA-sourced carbon for growth. This is also corroborated by the homogeneous distribution of the isotopic label over the entire cellular volumes (Fig. 2e).

Members of the *Ca*. Izemoplasmataceae particularly stood out in this study because they became highly labelled in the DNA-SIP analyses, significantly increased in relative abundances when DNA was added to sediments and harboured various genes strongly indicating that they could catabolically exploit various subcomponents of DNA. Some *Ca*. Izemoplasmataceae encoded up to five extracellular nucleases, providing a clear indication that they can digest environmental DNA. Some of the extracellular nuclease genes were directly colocalized with various genes encoding enzymes for further digestion of DNA into oligonucleotides and nucleosides, as well as removal of phosphates from nucleotides, and for transport of liberated nucleobases and phosphate into the cells. Colocalizations of genes for enzymes that carry out concerted metabolic processes are commonly found in genomes of microorganisms, and provide strong indications for functional and metabolic links^47,48^. For example, polysaccharide utilization loci encode suites of enzymes for deconstructing and importing diverse complex polysaccharides into cells^49^. The genomic colocalizations of the genes identified in *Ca*. Izemoplasmataceae therefore suggest that degradation and transport processes for extracellular DNA are carried out in a co-ordinated manner. This therefore indicated that they are important nutrient acquisition strategies for these organisms, and, to our knowledge, such multipartite gene organizations for DNA degradation have not been described in any other microorganisms.

Up to now, little was known regarding the ecological or biogeochemical roles of *Ca*. Izemoplasmataceae in marine sediments. One study of MAGs derived from marine sediments hinted that *Ca*. Izemoplasmataceae may catabolise nucleotides and the sugar moiety of DNA, among other key features such as the ability to grow via fermentation of few simple sugars^26^. Recently, a *Ca*. Izemoplasmataceae bacterium (*Xianfuyuplasma coldseepsis*) was shown to use DNA for growth in vitro, thereby providing further evidence for this functional trait^50^. Our analyses therefore greatly expand the ecophysiological understanding of *Ca*. Izemoplasmataceae by showing that they can actively participate in the primary degradation of extracellular DNA polymers in anoxic marine sediments. The genomic analyses also show that they are evolutionarily adapted for DNA catabolism, since they have an unprecedented high gene-dosage for extracellular nucleases, relatively small genomes and limited catabolic repertoire for organics other than DNA. We therefore propose that DNA may help them establish their primary niche in sediments. We also show that DNA-degrading capabilities are present in the *Ca*. Izemoplasmatales family UBA5603, which appears to be a sister clade of the marine *Ca*. Izemoplasmataceae that is primarily derived from the terrestrial subsurface. Genomes of the family UBA5603 did, however, lack many genes for enzymes required for de novo nucleotide biosynthesis, suggesting that they may degrade DNA for either, or both, salvage and/or catabolic purposes.

The detection of ^13^C-labelled *Shewanella* and *Lutibacter* in the DNA-SIP analyses served as a further indication that bona fide DNA-degrading bacteria were detected by our DNA-SIP approach. This is because some *Shewanella* strains are known to grow by catabolising DNA as a sole nutrient and energy source in vitro^19^. Similarly, members of the genus *Lutibacter* have been shown to possess hydrolytic activity for DNA in vitro, although it has not been reported whether they can grow using DNA as a sole nutrient^37^. This study therefore provides important confirmation that these functional properties are used under in situ-like conditions and that they may be important nutrient acquisition strategies for these bacteria in marine sediments. *Shewanella* are known metal-reducing bacteria, and their DNA-degrading activities may therefore be especially important in metal-rich Arctic sediments such as those studied here, or in subsurface redox zones where metal-oxides drive anaerobic respiration and organic carbon mineralization, and that are common to sediments^22,23^.

Bacteria affiliated with the family Fusibacteraceae were conspicuous for their strong responses to additions of individual purine-based nucleobases and nucleosides, while also being highly labelled in the DNA-SIP and NanoSIMS analyses. Intriguingly, we could not identify genes for known extracellular nucleases in the *Fusibacteraceae* MAG BB-3 or in close relatives, suggesting they were absent or unknown enzymes might be encoded. Because they had multiple 5ʹ-nucleotidases that were predicted to be embedded on their cell walls, as well as multiple gene clusters predicted to encode specific nucleoside/nucleotide transporters, we hypothesize that these organisms could be ‘cheaters’, that is, they may use subcomponents of DNA liberated into the sedimentary environment by other DNA-degraders. Until now, no Fusibacteraceae or closely related bacteria have been reported to have any activity related to DNA degradation or the degradation of molecular subcomponents of DNA.

Finally, screening of MAGs from other marine sediment metagenomes for genes for secreted nucleases identified potential DNA-degraders among various other marine sediment sites. Most notably, various Bacteroidota MAGs from all sediment sites have the capacity for extracellular DNA degradation. These encoded both predicted extracellular nucleases and periplasmic nucleotidases, strongly indicating capacity to use extracellular DNA. Together with our experimental data that showed active DNA degradation by *Lutibacter* (Bacteroidota) populations, this indicates Bacteroidota members are potentially among the most important players in DNA turn-over in marine sediments in general. This is supported by the fact that *Lutibacter* spp. and related Flavobacteriaceae are especially prevalent in various distinct marine sediments, for example, reaching relative abundances of 7–9% in sediments from the North Sea, East China Sea and Svalbard^51–53^. The potential for extracellular DNA degradation was also identified in various MAGs from the Chloroflexota (classes Dehalococcodia and Anaerolineae) and Archaea. These MAGs were mainly assembled from deeper subsurface sediments, that is, 24–54 cm below seafloor^43^. We therefore propose they may play roles in DNA turn-over in deeper, energy-limited sediments, where these groups are well known to persist and become dominant members of subsurface communities^54,55^. This may explain why Chloroflexota were not determined to take-up ^13^C-carbon from ^13^C-DNA in our experiment, which used samples from relatively shallow marine sediments that may be more dominated by relatively fast-growing taxa. These results also add to mounting evidence for diverse roles of uncultured and enigmatic Archaea in organic matter processing in the marine subsurface^3,56,57^. Future experimental work should also target Archaea, as PCR primers used in this study did not cover most Archaea. Together, these additional metagenomic analyses extend our understanding of microorganisms with capacity to use DNA as a nutrient source in marine sediments of varying physical and geochemical properties, and highlight members of the Bacteroidota as potentially important players.

## Methods

### Sediment sampling and microcosm set-ups

Marine sediments (0–30 cm) for microcosm experiments were collected from two locations in northern Baffin Bay (Stations ‘KANE-2B’ 79°30.909 −70°50.819 and ‘GL-111’ 76°18.354 −73°13.180) using a box corer aboard the Canadian Coast Guard icebreaker CCGS *Amundsen*, in July 2014. Sediments were placed into 250-ml Schott bottles as fast as possible and completely sealed to exclude oxygen, and then were maintained at 4 °C or on ice during transport. Additional sediments (0–5 cm) for analysis of in situ communities were also obtained from Stations GL-111 (2013–2016) and KANE-2B (2014), and were frozen immediately at −20 °C shipboard. The sediments for microcosms were stored for 4 weeks before the start of the experiment. All further sediment manipulations were performed in the laboratory within an anoxic glove-box (containing approximately 88% nitrogen, 2% hydrogen, 10% CO_2_) to minimize oxygen contact. While anaerobic taxa prevailed in our microcosms (Extended Data Fig. 3a), it was possible that highly oxygen sensitive populations may have had their activities reduced due to slight exposure of sediments to oxygen during sampling. Sediments from both sites were mixed together in equal volumes, and then amended with cold (4 °C), anoxic artificial seawater (1:1 v/v) that included resazurin as a redox indicator. Resulting sediment slurries were homogenized thoroughly within an anoxic glove-box. Approximately 30 ml of sediment slurry was added to 100-ml serum flasks, and these were sealed and crimped with 2-cm-thick black butyl-rubber stoppers. Sediment slurries and microcosms were placed on top of frozen ice-packs during all preparation or manipulation steps in the anoxic glove-box. Microcosms were maintained at 4 °C during all subsequent incubations.

### Preparation of ^13^C- and ^12^C-labelled DNA for use as substrate

**Extended Data Fig. 10:**
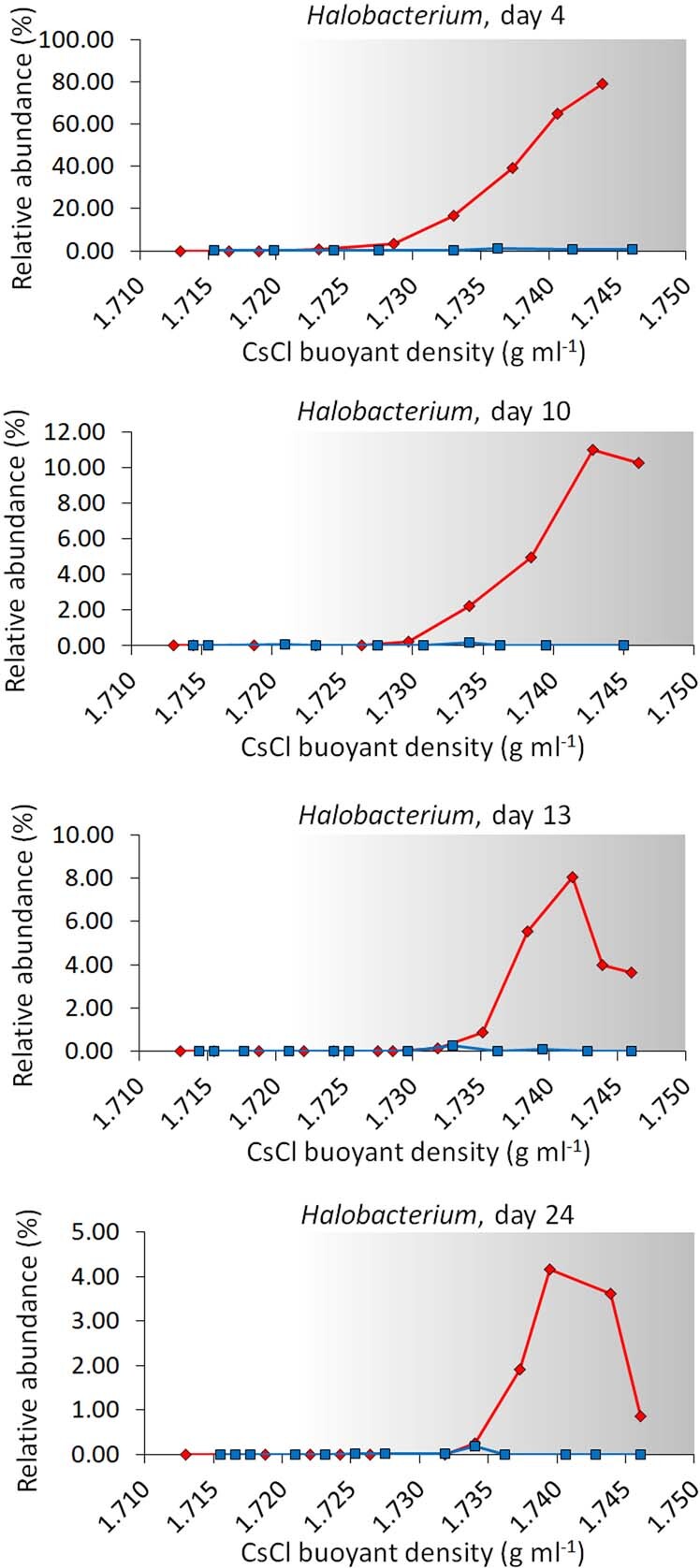
Relative abundances of archaeal *Halobacterium salinarum* 16S rRNA genes across isopycnic density gradient fractions, as determined by MiSeq amplicon sequencing. The archaeal DNA was added as substrate to microcosms. Relative abundances determined across ^13^C and ^12^C gradients are plotted in red diamonds and blue squares, respectively. The grey shaded areas represent the ‘heavy’ ends of the gradients. Source data

*H. salinarum* was grown in a medium with 1.25 g of NaCl, 0.1 g of MgSO_4_, 0.04 g of KCl, 0.006 g of sodium citrate and ‘carbon mix’ 0.05 g, per 5 ml of double distilled H_2_O (ddH_2_O). ‘Carbon mixes’ were either (1) ISOGRO-^13^C Powder-Growth Medium (99 at% ^13^C) (Sigma-Aldrich, cat. no. 606863-1G), or (2) ^12^C yeast extract (Oxoid, cat. no. LP0021B). Cultures were grown for three generations for up to 3 d. DNA was extracted using a CTAB-phenol method after cells were pelleted and supernatant removed. Briefly, pelleted cells were suspended in 400 µl of extraction buffer (100 mM Tris-HCl (pH 8.0), 100 mM sodium EDTA (pH 8.0), 100 mM sodium phosphate (pH 8.0), 1.5 M NaCl, 1% (wt/vol) cetyltrimethylammonium bromide) (Sigma-Aldrich, cat. no. H6269). An additional 55 µl of SDS (20%, wt/vol) was also added. This was transferred to a Lysing Matrix E 2-ml tube (MP Biomedicals) and the samples were subjected to bead beating at ‘speed 6’ for two rounds of 30 s using a FastPrep-24 bead beating instrument (MP Biomedicals), with cooling on ice between. The supernatant was decanted into a clean tube after centrifugation for 10 min at 6,000*g*. An equal volume of phenol/chloroform (containing 4% (vol/vol) isoamyl alcohol) was then added and the samples were mixed by inversion, and then centrifuged at 16,000*g* for 10 min at 25 °C. The aqueous phase was collected into a new tube and precipitated with 0.6 volume of isopropanol at 4 °C for 30 min. The precipitate was pelleted by centrifugation at 16,000*g* for 30 min at 4 °C and the supernatant removed carefully, and then washed with 70% (vol/vol) ethanol and air dried for 5 min. The DNA was finally resuspended in 100 µl of ddH_2_0. DNA extracts from the *Halobacterium* were washed with a Microcon YM-10 (Millipore, cat. no. Z648078) device to reduce residual low molecular solutes less than approximately 10 kDa. The extracts had 260/280 ratios averaging around 2.0 as determined by spectrophotometry using a Nanodrop-1000 device (Nanodrop). The highly gelatinous consistency of the purified extract further indicated that DNA was the major organic compound in the extract. The ^13^C-enriched nature of the *Halobacterium* DNA extract was demonstrated by examination of *Halobacterium* 16S rRNA gene relative abundances in SIP density gradients, which showed these sequences dominating in the most dense (‘heaviest’) fractions retrieved (Extended Data Fig. 10).

### Microcosm incubation conditions and subsampling

Microcosms were pre-incubated for 4 d before substrate additions, and this time point when substrates were added is herein referred to as day 0. Substrates were also added directly after subsampling at day 10. Purified ^13^C-labelled or unlabelled DNA extracted from *H. salinarum* (Extended Data Fig. 10), which is not present at detectable abundances in typical marine sediments, was added as substrate at concentrations of 36 µg ml^−1^. This amount was added because it is similar to that of total bioavailable DNA previously measured in marine sediments^10^. The nucleobases and nucleosides were all purchased from Sigma-Aldrich (>99% pure) and included adenine (cat. no. A8626), guanine (cat. no. G6779), cytosine (cat. no. C3506), thymine (cat. no. T0376), 2-deoxyadenosine (cat. no. D7400) and thymidine (cat. no. T9250), which were added individually to separate microcosms, each to final concentration of 100 µM. Parallel microcosms without added substrates were analysed as ‘no-substrate’ controls. All treatment series were performed in triplicate microcosms.

Initial samples for microbial community analyses were taken directly after preparation of the sediment slurry, and following substrate amendments subsamples were taken for microbial community profiling, DNA-SIP, CARD-FISH and metagenomics at days 0, 4, 10, 13, 24 and 31. For microbial community profiling via 16S rRNA gene amplicon sequencing, 100 µl from each microcosm was subsampled. Additional 1-ml subsamples were taken at each time point for chemical analyses. Subsamples were kept on ice-packs inside the anoxic glove-box during subsampling, and were then immediately snap-frozen on dry-ice outside the glove-box and subsequently stored at −80 °C. For CARD-FISH, 500-µl samples were fixed with 4% formaldehyde in phosphate-buffered saline pH 7.5 (PBS) for 4 h at 4 °C, washed twice with PBS and stored in PBS/ethanol (1:1) at −20 °C using standard procedures^58^. All tubes for subsampling were introduced into the anoxic glove-box at least 1 h before subsampling to remove traces of O_2_. Headspace gas (15 ml) was subsampled from microcosms using N_2_-flushed syringes fitted with needles and injected into pre-evacuated 12-ml glass exetainers (Labco) that were sealed with butyl-rubber stoppers.

### DNA extractions from sediments

DNA for 16S rRNA gene amplicon sequencing and for density gradient centrifugations was extracted from 100 µl of microcosm slurries using a combination of bead beating, a cetyltrimethylammonium bromide-containing buffer and phenol/chloroform extractions (detailed above). DNA for metagenomic library preparation was purified from 100-µl microcosm subsamples using a MoBio Ultra Pure PowerSoil Kit (MoBio, cat. no. 12888-50) following the manufacturer’s instructions. DNA for 16S rRNA gene amplicon sequencing of sediment samples collected and preserved in the field was extracted from 0.25 g of sediment using Qiagen DNeasy PowerSoil Pro kit (Qiagen, Cat. no. 47014), following the manufacturer’s instructions with two modifications as previously published^59^. Bead beating was performed at 5.5 m s^−1^ for 45 s with a Bead Ruptor 24 (OMNI).

### Density gradient centrifugations

DNA from triplicates of specific treatments and time points to be analysed by DNA-SIP was combined equally to a total of 500 ng and subject to ultracentrifugation in a CsCl solution with a VTi-90 rotor for 72 h following standard protocols^60^. Gradients were collected by fractionation into approximately 20 separate fractions of approximately 200 μl each. The densities of the collected fractions were measured using a refractometer (AR200; Reichert Analytical Instruments) at 22 °C. DNA was concentrated and purified by precipitation with polyethyleneglycol (molecular weight = 8,000) (Sigma-Aldrich, cat. no. P5413) and glycogen (Thermo Scientific, cat. no. R0561), and subsequently washed using methods previously described^60^. DNA was diluted 1:100 in ddH_2_O to facilitate PCR amplification.

### PCR and quantitative PCR amplification

PCR amplifications of 16S rRNA genes from DNA extracted from microcosms and density gradient centrifugation fractions were performed using a two-step PCR barcoding approach^61^. This was done using primers to target most Bacteria (341F: 5ʹ-CCTACGGGNGGCWGCAG-3ʹ and 785R: 5ʹ-GACTACHVGGGTATCTAATCC-3ʹ)^62^, and to avoid co-amplification of the added archaeal DNA from *H. salinarum*. This was done to avoid *Halobacterium* sequences possibly taking up high proportions of the resulting sequence data. Both primers included ‘head’ sequences (5ʹ-GCTATGCGCGAGCTGC-3ʹ) to facilitate barcoding in the second-step PCR^61^. PCR reactions for amplifying 16S rRNA genes from DNA-SIP gradients and microcosms were performed as follows: the first-step PCRs (total volume of 12.5 μl) contained 7.45 μl of ddH_2_0, 12.5 μl of 10× Taq Buffer (Thermo Scientific), 250 μM dNTP mix, 1.5 mM MgCl_2_, 0.2 μM of each primer, 0.05 μl of Thermo Scientific Taq DNA Polymerase (Thermo Scientific, cat. no. 11146165001) and 0.5 μl of DNA template (1:100 dilutions of crude extracts). PCR primers were 341F (5ʹ-CCTACGGGNGGCWGCAG-3ʹ) and 785R (5ʹ-GACTACHVGGGTATCTAATCC-3ʹ). PCR conditions for the first-step PCR were: 95 °C for 2 min; followed by 30 cycles of 95 °C for 30 s, 52 °C for 30 s and 72 °C for 30 s; followed by a final extension of 72 °C for 2 min. For the second-step barcoding PCRs, 1 μl of the first-step PCR was added to 25-μl PCRs containing the same concentrations of reagents detailed as for the first-step PCR, except the barcoded primer was 0.4 μM in each reaction. PCR conditions for the second-step PCR were the same as for the first-step PCR, except a total of 15 cycles were performed. PCR products were purified using a ZR-96 DNA Clean-up Kit (Zymo Research) and pooled to equimolar concentrations after quantification using the Quant-iT PicoGreen dsDNA Assay Kit (Thermo Fisher Scientific), or purified and normalized in a single step using the SequalPrep normalization kit (Thermo Fisher Scientific), and pooled and concentrated on columns (Analytik Jena) thereafter. Sequencing was performed by MicroSynth (Switzerland) and by the Joint Microbiome Facility (JMF) (Vienna, Austria) on Illumina MiSeq instruments using MiSeq Reagent Kit v3 chemistry with 300-base pair (bp) paired-end read mode. Sequencing libraries prepared by the JMF were prepared using the TruSeq Nano DNA Library Preparation Kit (Illumina). Negative DNA extract controls (no added sediment) and PCR negative controls (no DNA template) were performed and sequenced in parallel (even if no PCR product was detected).

PCR reactions for amplifying 16S rRNA genes from sediments collected and preserved in the field were performed as follows: PCRs (total volume of 25 μl) contained 6.5 μl of ddH_2_0, 12.5 μl of 2× KAPA HiFi Hot Start Ready Mix (KAPA, cat. no. KK2601), 2.5 μl (0.1 μM final) of each primer and 1 μl of DNA template (5 ng µl^−1^). PCR conditions were: 95 °C for 3 min; followed by 30 cycles of 95 °C for 30 s, 60–55 °C for 45 s (touchdown of 1 °C per cycle for the first 10 cycles, followed by 55 °C for remaining cycles) and 72 °C for 60 s; followed by a final extension of 72 °C for 5 min. PCR primers were 515F (5ʹ-GTGYCAGCMGCCGCGGTAA-3ʹ)^63^ and 806R (5ʹ-GGACTACNVGGGTWTCTAAT-3ʹ)^64^. PCR reactions were run in triplicate, followed by pooling and clean-up using NucleoMag NGS Clean-up and Size Select beads (Macherey-Nagel, cat. no. 744970.50). A second-step barcoding PCR was performed with index primers, followed by another bead-based clean-up using NucleoMag NGS Clean-up and Size Select beads. The Illumina-Nextera barcoded amplicon libraries were pooled in equimolar proportions and sequencing was performed using a MiSeq sequencer (Illumina) using the 2 × 300 bp MiSeq Reagent Kit v3.

Quantitative PCR assays for bacterial 16S rRNA genes from fractions collected from CsCl gradients were analysed using qPCR assays targeting Bacteria using the primers 341F (5ʹ-CCTACGGGAGGCAGCAG-3ʹ) and 534R (5ʹ-ATTACCGCGGCTGCTGGCA-3ʹ). Quantitative PCR assays for *H. salinarum* DNA were performed using the primers proX-F (5ʹ-CAGGACGGAACGCAGAGAGAA-3ʹ) and proX-R (5ʹ-ACTACCGCCATACCAAGACC-3ʹ).

Quantitative PCR reactions (10 μl total) contained 5 μl of iQ SYBR Green Supermix (BioRad, cat. no. 1708880), 0.5 μM of each primer, 3 μl of ddH_2_0 and 1 μl of DNA template (1:100 dilutions of crude extracts). PCR conditions were: 95 °C for 3 min; followed by 40 cycles of 95 °C for 30 s, 60 °C for 30 s for bacterial 16S rRNA gene assay or 55 °C for the *Halobacterium* assay, and 72 °C for 30 s; followed by a final extension of 72 °C for 2 min. Standards for 16S rRNA genes included a 16S rRNA gene derived from an in-house culture of *Desulfosporosinus acidiphilus*, which was cloned into a pPCR4-TOPO vector (Invitrogen) and reamplified by PCR using primers M13F (5ʹ-GTAAAACGACGGCCAG-3ʹ) and M13R (5ʹ-CAGGAAACAGCTATGAC-3ʹ), and then PCR purified and diluted to a range from 10^9^ to 10^3^ copies per microlitre. Genomic DNA from *H. salinarum* was used as standard for the *H. salinarum*-specific assay, and was diluted to a range from 10^9^ to 10^3^ copies per microlitre. All qPCR reactions were performed in triplicate. Quantitative PCR was performed with a CFX96 Touch Real-Time PCR Detection System (v.3.1) (BioRad). A melting curve analysis was also performed after the final extension using default instrument settings, whereby the temperature was increased from 65 °C to 95 °C.

### Bioinformatic processing of 16S rRNA gene amplicon data and statistics

The sequencing of 16S rRNA gene amplicons was performed as previously described^61^. Demultiplexed read pairs were assembled into ASVs using DADA2 (ref. ^65^) with the recommended workflow^66^. ASV sequences were subsequently classified using the Naïve Bayesian classifier^67,68^. A confidence cutoff of 50% was used for classification with the SILVA 119 SSU NR99 database as taxonomic reference^69^.

The IMNGS webserver^70^ was used to identify the presence and relative abundances of sequences related to ASVs representative of the ^13^C-labelled taxa identified by SIP, in different publicly available Short Read Archive (SRA) datasets. Sequence identity cut-offs of ≥95% for *Shewanella*, *Ca*. Izemoplasma and *Fusibacteraceae* ASVs, and ≥97% identity to *Lutibacter* ASVs, were used when querying the SRAs via IMNGS (Extended Data Fig. 8). A minimum overlap of 100 bp was required between query and database sequences.

### Statistical analyses of 16S rRNA gene amplicon sequencing data

All statistical analyses of 16S rRNA gene amplicon data were performed using the Rhea package (v.1.0.1-5)^71^ as implemented in the R software environment (v.1.1.383). To detect ASVs enriched in relative abundances in the dense fractions of CsCl density gradients from ^13^C versus ^12^C treatments, the ‘Serial Group Comparisons’ pipeline of Rhea was applied. Significant differences in relative abundances of taxa were compared among multiple heavy fractions of gradients from ^13^C- versus ^12^C-DNA treatments, and in two ‘windows’ of heavy fractions (Extended Data Fig. 1 and Supplementary Table 2). More specifically, relative abundance data of ASVs from 3–5 heavy fractions, ranging from 1.725 to 1.741 g ml^−1^ (window 1) and from 1.735 to 1.746 g ml^−1^ (window 2), from ^13^C-DNA amended versus ^12^C-DNA amended controls, for days 4, 10, 13 and 24, were used as input (Supplementary Table 2). The statistical analysis used the Wilcoxon signed rank sum test to test for significant differences in ASV relative abundances among treatments, as implemented in Rhea^72^. Default parameters were used except for the following: abundance_cutoff <0.001, prevalence_cutoff <0.1; max_median_cutoff <0.001; ReplaceZero = ‘NO’.

To identify ASVs that were enriched in relative abundances in the microcosm sediments of treatments that received supplementation of DNA or DNA subcomponents versus the sediments of parallel, no-substrate control microcosms, for each time point, the ‘Serial Group Comparisons’ pipeline of Rhea was applied using data from triplicate microcosms (Extended Data Fig. 1). Default parameters were used except for the following: abundance_cutoff <0.05, prevalence_cutoff <0.1; max_median_cutoff <0.001; ReplaceZero = ‘NO’. Only ASVs that were significantly enriched in relative abundances in treatments that received supplementation of DNA or DNA subcomponents, at two or more time points for each treatment series, were reported.

For beta diversity analysis of microbial communities in microcosms, principal coordinate analysis was performed using a Bray–Curtis dissimilarity matrix (including relative abundances). The plots were constructed with the ‘vegan’ package (v.2.5-3) in R. To avoid biases related to differences among library depths, sequencing libraries were all subsampled to a number of reads smaller than the smallest library (1,620 reads).

### Comparisons of microbial taxa in initial microcosms and in situ sediments

To assess the microbial taxa present in initial microcosms with in situ sediments, we compared datasets from microcosms in this study (generated with domain Bacteria-targeting primers 341F/785R) versus from in situ sediments (generated using universal primers targeting Bacteria and Archaea, 515F/806R), and processed sequence reads together using *mothur*^68^. Contigs from forward and reverse reads were produced using the make.contigs command, then sequences with ambiguous bases and those that exceeded 2 ± s.d. of average contig lengths were excluded. All sample read datasets were subsampled to a maximum of 5,000 sequences. Sequences were then classified to the genus level with the Naïve Bayesian classifier^67^, with a confidence cutoff of 50% and the SILVA 119 SSU NR99 database as taxonomic reference^69^.

### Metagenomic library preparation, sequencing and analyses

Metagenomic libraries were prepared for sequencing and indexed using the Nextera XT DNA Library Preparation Kit (Illumina, cat. no. FC-131-1024), following the manufacturer’s instructions. Five samples were selected to facilitate differential coverage binning, that is, (1) the initial sediment slurry; (2) day 4, ^12^C-DNA treatment; (3) day 13, ^13^C-DNA treatment; (4) day 24, ^12^C-DNA treatment; and (5) day 31, ^13^C-DNA treatment. DNA libraries were purified using magnetic beads via the Agencourt AMPure XP kit (Beckman Coulter, cat. no. A63881), and sequenced by the Vienna BioCenter Core Facilities on one lane of an Illumina HiSeq 2500 instrument using HiSeq V4 chemistry with 125-bp paired-end mode.

Raw sequences were trimmed to 100 bp, and each sample dataset was assembled separately using IDBA-UD (v.1.1.1) using default parameters^73^. Assembled contigs >1,000 bp were automatically binned into MAGs based on a combination of nucleotide coding frequencies and sequence assembly coverage using MetaBat2 using each present binning strategy (v.2.12.1)^74^, MaxBin2 (v.2.2.4)^75^ and CONCOCT (v.0.4.1)^76^. Coverage profiles for binning were acquired by mapping trimmed reads to assemblies using BWA^77^ and SAMtools^77^. Additionally, the sample from the day 31 ^13^C-DNA treatment was also assembled using MetaSpades (v.3.11.1)^78^ and additionally binned with MetaBat, MaxBin and CONCOCT. Genome bins from each binning tool and from each respective assembly were aggregated using DasTool (v.1.1.0)^79^. All genome bins were finally dereplicated using dRep (v.1.4.3)^80^, with the following options: all genomes were dereplicated using an ANI cut-off value of ≥98% in the secondary ANI comparison, and of the dereplicated genomes, those that were >50% complete and with <10% contamination were kept. The completeness and degree of potential contamination of the genome bins were evaluated by CheckM (v.1.0.7)^81^. Gene calling and initial genome annotations were performed via RAST^72^. Putative functions of predicted proteins were also checked via BLASTP^82^ against the NCBI-nr database and evaluated in relation to relevant literature. Predictions of subcellular locations of predicted proteins were performed using PSORTb (v.3.0)^83^, with settings appropriate for the predicted cell wall types of the respective organism. Biochemical inferences were primarily based on the MetaCyc database^84^. Gene synteny depictions were produced using EasyFig^85^.

### Screening of additional MAGs

Additional MAGs derived from marine sediment metagenomes were manually retrieved from publicly available datasets^39–43^. MAGs were initially screened by CheckM as described above, and all MAGs with >10% contamination were removed. MAGs from Svalbard sediments^39,40^, permeable sandy sediments from Australia^41^ and non-hydrothermal ‘control’ sediments from the Gulf of Mexico^42^ were annotated via RAST as described above. For MAGs from the White Oak River dataset^43^, all protein annotations for each MAG were downloaded from the Integrated Microbial Genomes and Microbiomes (IMG/M) server^32^. All proteins were subsequently subjected to PSORTb (v.3)^83^ with settings appropriate for the predicted cell wall types of the respective organism. All predicted secreted enzymes with catabolic potential for DNA and subcomponents of DNA were checked for Sec and TAT signal peptides using SignalP (v.5.0)^86^. Nuclease domains were checked by CD-search against the Conserved Domain Database (CDD) using default settings (<e-value 0.01)^87^.

### Phylogenetic and ANI analyses

All phylogenetic analyses of 16S rRNA gene sequences were performed in ARB (v.6.0.6) using maximum likelihood algorithms^88^ and the SILVA 132 SSU NR99 database^69^. Query sequences were first aligned to the SILVA alignment with the SINA aligner^79^, then sequences were inserted into the global reference tree using the parsimony option. A selection of near-full-length sequences of close relatives and cultivated relatives of the query sequences were then selected and used to construct trees de novo using RaxML^80^, fastDNAmL^89^ and PhyML^90^ algorithms as implemented in ARB with default settings and the ARB bacterial ‘Positional variability by parsimony’ filter. A consensus tree was then created with those three trees and short amplicon sequences were inserted into the consensus tree via the parsimony option of ARB.

Phylogenomic analyses were conducted by using an alignment of concatenated protein sequences derived from 43 single-copy marker genes retrieved from the CheckM analyses. A tree was first constructed using these protein alignments derived from the query genomes and 8,203 representative genomes downloaded from the NCBI database (as of July 2018). The tree was constructed using default parameters using IQ-TREE webserver with automatic substitution model selection and ultra-fast bootstrapping (1,000×)^91^. Manually selected reference sequences that were taxonomically informative for query sequences were obtained, and a second smaller tree was reconstructed using only the selected reference and query sequences.

ANIs for comparison of genome relatedness were determined using JSpeciesWS server^92^, based on BLAST (‘ANIb’).

### Taxonomic names

In addition to 16S rRNA-based SILVA taxonomies, we used the newly proposed names of the GTDB (v.0.1.3), which is based on genome phylogeny^93^.

### Chemical measurements

Carbon isotope compositions of CO_2_ (δ^13^C values in permille relative to Vienna Pee Dee Belemnite) were analysed by a headspace gas sampler (Gas-Bench II, Thermo Fisher) coupled to an isotope ratio mass spectrometer (Delta V Advantage, Thermo Fisher). CO_2_ reference gas was calibrated using ISO-TOP gas standards (Air Liquide) with a certified ^13^C concentration.

For total sediment iron and manganese, inductively coupled plasma optical emission spectrometry measurement was applied on a Perkin Elmer 5300 DV (Pekin Elmer) after total fusion of 0.1-g oven-dried sediment samples (1 g of original sample dried for 3 h at 105 °C, loss of ignition 6 h at 600 °C, followed by 6 h at 1,000 °C, loss on ignition approximately 80%) with 0.9 g of di-lithium tetraboarate (Spectromelt A10, Merck, cat. no. 1107831000) using a Linn Lifumat, 9 min at 1,050 °C (Linn High Therm). The fusion product was dissolved in 50 ml of 5 M HNO_3_ (Normapur, VWR, Double sub-boiled, Berghof BSB-939-IR) with 150 ml of deionized water (MilliQ, Merck-Millipore), and further diluted to a total volume of 250 ml. External linear matrix matched calibration was applied using single-element manganese and iron standards (Merck-Millipore), including quality control with LKSD1 and LKSD4 certified lake sediment standards (Natural Resources Canada).

### Cell sorting and single-cell SIP analysis by NanoSIMS

Cells of microorganisms from formaldehyde-fixed microcosm sediment samples were extracted from previously fixed sediments that were stored in PBS/ethanol (1:1) at −20 °C. Ethanol was removed from PBS/ethanol samples (500 µl) by pelleting and washing twice with PBS. Samples were diluted in 1.8 ml of PBS, and then sodium pyrophosphate (0.1% final) and Tween 20 (0.5% final) were added. They were vortexed for 20 min at medium speed (4–5) with a Vortex Genie 2 vortexer (Scientific Industries), with tubes closed with parafilm and taped down horizontally. Samples were sonicated with 50% power for 20 s with setting ‘5’, on ice (UW 2070 needle, Bandelin Electronics). Cell suspensions were made up to 4 ml with PBS in 13.2-ml Thinwall Polypropylene Tubes (Beckman Coulter), and then 2 ml of Nycodenz solution (80% w/v) (Alere Technologies, cat. no. 1002424) was injected carefully under the cell suspension by a long needle and syringe. Samples were centrifuged for 90 min at 4 °C in an SW 41 Ti Swinging-Bucket Rotor (Beckman Coulter) at 14,000*g*, with no deceleration when stopping. Total supernatant was collected into a new tube, and then ethanol was added to produce a 1:1 final solution. The collected solutions were stored at −20 °C.

For CARD-FISH standard protocols and buffers were used^58^. Samples of ~500 µl were filtered onto polycarbonate filters (GTTP type, 0.2-µm pore size) (Millipore, cat. no. GTTP02500), and then PBS (5 ml) was washed through. Filters were dried at 46 °C for 5 min before CARD-FISH. The newly designed 5ʹ-horseradish peroxidase-labelled (HRP) probe Fusi-6-HRP (5ʹ-TTCCTTAGGTACCGTCATTTTTCT-3ʹ) (Biomers) and the unlabelled helper probe Fusi-6-HelpR (5ʹ-GGCACGTATTTAGCCGGTGC-3ʹ) were used for hybridizations targeting populations representing the most abundant Fusibacteraceae ASV. The NONEUB probe (5ʹ-ACTCCTACGGGAGGCAGC-3ʹ) was used for negative controls and to gauge background fluorescence during FACS. The Fusi-6-HRP probe was designed to specifically target the most abundant Fusibacteraceae ASV (ASV_09916) and relatives in our 16S rRNA gene amplicon sequence dataset. The probe also matched other (*n* = 25) related Fusibacteraceae ASVs (all >91% sequence identity to ASV_09916), although those sequences were generally in low abundance (<0.5% on average across microcosms and time points). Although few other ASVs from other taxa also matched (*n* = 20), they all represented ASVs with extremely low abundances, that is, the most abundant of these ASVs had a maximum relative abundance of 0.0003%. When checked using the ProbeMatch function in SILVA^94^, only 29 hits were obtained for Fusibacteraceae sequences, and only one off-target match came from the Cyanobacteria. We therefore deemed that the Fusi-6 probe should primarily detect the abundant Fusibacteraceae populations.

Hybridizations of probes were performed overnight (~14–16 h) at 35 °C with 50% formamide, using previously described protocols and using lysozyme (Sigma-Aldrich, cat. no. 62970) permeabilisation^58^, and Oregon-Green 488-labelled tyramides (Thermo Fisher, cat. no. T20919). The overnight hybridization time enabled penetration of probes into the cells. The hybridizations were performed with whole filters, where the filters were carefully added to 2-ml tubes with 300 µl of CARD-FISH hybridization buffer with probes, mixed and placed horizontally during hybridization so the buffer covered most of the filters. After probe washing, and after tyramide signal amplification and washing^58^, cells were scrapped off filters by adding filters to the lids of 50 ml tubes (Falcon) with the side with cells facing up, and 200 µl of PBS was added to cover the top. The surfaces of the filters were then scraped gently with a cell-culture scraper (with 1.3-cm flexible blade, Techno Plastic Products) for 30 s. The PBS solution with cells was then pipetted to a clean tube, and stored on ice and in the dark before cell sorting via FACS. To check hybridizations, parallel samples that were not scraped off filters were performed and stained with 4,6-diamidino-2-phenylindole (DAPI), and visualized with an inverted Leica TCS SP8X CLSM using appropriate excitation/emission settings for DAPI and the Oregon-Green 488-labelled tyramides.

For cell sorting, cells from CARD-FISH were resuspended with 1.8 ml of PBS, gently filtered through a 35-µm cell strainer (Corning) and sorted in ‘purity-mode’ using a BD FACSMelody Cell Sorter (BD Biosciences). Hybridized cells were detected with green fluorescence (using manufacturer’s’ ‘FITC’ settings) and forward scatter, and sorting gates were placed higher than background fluorescence determined from NONEUB controls that were measured earlier. Approximately 5,000–7,000 cells were sorted directly onto polycarbonate filters (0.2-µm pore size, hydrophilic polycarbonate membrane, 47-mm diameter, Millipore), pre-coated with AuPd thin films (nominal thickness of 120 nm, obtained by sputter-deposition), which were placed on microscope cover-slips and on the 2-ml sort-tube holder. The flow rate for sorting was slowed to around 500 events per second, so that excess fluid did not build up and spread over the filters, thereby ensuring that cells would be sorted to, and dry on, a small area of the filter. After sorting, filters were air dried, and then washed by carefully pipetting 200 µl of 80%, 60%, 40% and 20% PBS, followed by deionized water, onto the filters (each for 30 s), until no salt deposits could be observed. Filters were finally air dried, and kept dry and in the dark at 20 °C until NanoSIMS.

For NanoSIMS analysis, the collected cells on AuPd-sputtered polycarbonate filters were measured by NanoSIMS on a Cameca NS50L instrument (France) at the Large-Instrument Facility for Environmental and Isotope Mass Spectrometry of the University of Vienna. The detectors of the multicollection assembly were positioned to enable parallel detection of ^12^C_2_^−^, ^12^C^13^C^−^,^12^C^14^N^−^, ^31^P^−^ and ^32^S^−^ secondary ions. Secondary electrons were detected simultaneously for gaining information about the sample morphology and topography. Before data acquisition, analysis areas were pre-conditioned in situ by rastering of a high-intensity, defocused Cs^+^ ion beam in the following sequence of high energy (HE, 16 keV) and extreme low ion impact energy (EXLIE, 50 eV):: HE at 100-pA beam current to a fluence of 5.0 × 10^14^ ions per cm^2^; EXLIE at 400-pA beam current to a fluence of 5.0 × 10^16^ ions per cm^2^; HE to a fluence of 5 × 10^14^ ions per cm^2^. Data were acquired as multilayer images obtained by sequential scanning of a finely focused Cs^+^ ion beam (~70-nm probe size at 1.5-pA primary ion current) over a field size of 60 × 60 µm^2^ at 512 × 512-pixel image resolution with a per-pixel dwell time of 10 ms per cycle. Image data were processed by using the WinImage software package (v.2.0.8) provided by Cameca. Before stack accumulation, the individual images were drift corrected. Secondary ion signal intensities were corrected for detector dead time (44 ns) on a per-pixel basis. Carbon isotope composition images displaying the ^13^C/(^12^C + ^13^C) isotope fraction, designated as at% ^13^C, were inferred from the C_2_^−^ secondary ion signal intensity distribution images via per-pixel calculation of ^13^C^12^C^−^/(2 × ^12^C^12^C^−^ + ^13^C^12^C^−^) intensity ratios. Regions of interest referring to individual cells were defined manually, based on the ^12^C^14^N^−^ and ^31^P^−^ secondary ion maps as indicators of cellular biomass, and cross-checked by the morphological features displayed by the secondary electron maps. ^13^C natural abundance values for bacterial biomass were obtained from analysis of single cells (performed in the same NanoSIMS measurement session) from a pure culture of *Eggerthella lenta*, grown in yeast extract-casitone-fatty acid medium with natural isotopic composition. FACS-sorted cells from the ^13^C-DNA incubations were assessed as significantly enriched in ^13^C if (1) the ^13^C isotope fraction was above the 99.9% confidence interval of the values determined on the cells from the natural abundance control and (2) the statistical counting error (3σ, Poisson) was smaller than the difference between the considered enriched cell and the mean value measured on the control. The Poisson error was calculated from the secondary ion signal intensities via 𝜎_Pois_ = 1/(^12^C^−^ + ^13^C−)^2^ × sqrt((^12^C^−^)^2^ × ^13^C^−^ + (^13^C^−^)^2^ × ^12^C^−^).

### Reporting Summary

Further information on research design is available in the Nature Research Reporting Summary linked to this article.

## Supplementary information

Supplementary Information Supplementary methods, results and discussion; Fig. 1; and Data 1.

Reporting Summary

Supplementary Table 1 Supplementary Tables 1–6.

## Data Availability

All sequence data were deposited under GenBank Bioproject PRJNA510104. PCR-derived 16S rRNA gene amplicon sequence data performed by Microsynth (microcosms) are available under accessions SAMN10603326–SAMN10603488. PCR-derived 16S rRNA gene amplicon sequence data performed by the JMF (DNA-SIP gradients) are available under accessions SAMN13338678–SAMN13338783. Metagenomic sequence read data from Greenland microcosms are available under accessions SAMN10594394–SAMN10594398. Metagenome-assembled genomes from Greenland microcosms are available under accessions SAMN10805732–SAMN10805736 and SAMN12272019–SAMN12272029. Metagenomic sequence read data from Svalbard marine sediments are available under Bioproject accessions PRJNA493859–PRJNA623111. Metagenome-assembled genomes from Svalbard sediments are available under Bioproject PRJNA623111 and accessions JADWMF000000000–JADWMK000000000. The 16S rRNA gene amplicon sequence data for the in situ communities are available under Bioproject accession PRJNA682441 and SRA accessions SAMN16990562–SAMN16990567. Previously generated metagenomic datasets and metagenome-assembled genomes that were reanalysed in this study are available under NCBI-GenBank Bioprojects: PRJNA270657 (White Oak Estuary, USA); PRJNA515295 (sandy sediments, Australia); and PRJNA362212 (Guaymas Basin, USA). Databases used were: Genome Taxonomy Database (GTDB) v.0.1.3 (https://gtdb.ecogenomic.org/); IMNGS webserver (as of November 2018) (https://www.imngs.org/); Integrated Microbial Genomes and Microbiomes (IMG/M) server (https://img.jgi.doe.gov/); SILVA database for ProbeMatch server (https://www.arb-silva.de/search/testprobe/); Conserved Domain Database (CDD) search server (https://www.ncbi.nlm.nih.gov/Structure/cdd/cdd.shtml); SILVA 119 SSU NR99 database (https://www.arb-silva.de/download/arb-files/); Short Read Archive (https://www.ncbi.nlm.nih.gov/sra); NCBI-nr (https://www.ncbi.nlm.nih.gov/protein/); and MetaCyc database (https://metacyc.org/). Source data are provided with this paper.

## Source data

Source Data Fig. 1 GenBank numbers of reference sequences used in phylogenetic tree. 16S rRNA gene sequences of query sequences added to the tree. The tree was based on the global SILVA alignment for 16S rRNA genes^94^. Relative abundances of 16S rRNA gene sequences in microcosms from taxa presented in Fig. 1. Relative abundances of 16S rRNA gene sequences in SIP gradient fractions used for statistical comparisons, and presented in Fig. 1. Source Data Fig. 2 NanoSIMS at% ^13^C values. Raw microscopy images (as TIF files) from Fig. 2 are available at https://doi.org/10.6084/m9.figshare.14387855.v1. Source Data Fig. 3 Phylogenome tree sequence names. Alignment fasta used for constructing tree. All GenBank accessions for original genomes or MAGs are provided in parentheses of sequence names. Gene/enzymes annotations for all tree annotations are listed in Supplementary Table 5—List of key annotations for DNA degradation in MAGs. Source Data Extended Data Fig. 2 Headspace CO_2_ isotope compositions. Relative abundances of *Halobacterium salinarum* in microcosms (16S rRNA gene amplicons). Quantitative PCR of *Halobacterium salinarum* DNA in microcosms. Source Data Extended Data Fig. 3 Relative abundances of major family-level lineages in microcosms (16S rRNA gene amplicons). Source Data Extended Data Fig. 4 Quantitative PCR results from DNA-SIP gradients. Source Data Extended Data Fig. 6 Summed relative abundances of 16S rRNA genes of genus/family-level clades of ^13^C-labelled taxa, across DNA-SIP gradients. Source Data Extended Data Fig. 8 SRA accessions and relative abundances of 16S rRNA gene sequences of taxa identified by IMNGS analysis. Source Data Extended Data Fig. 10 Relative abundances of *Halobacterium salinarum* 16S rRNA genes across DNA-SIP gradients.

## Code availability

Custom code for metagenome sequence read trimming is available from: https://github.com/kwasmund/Trim-Illumina.git.
