# Publisher Correction: Genomic insights into diverse bacterial taxa that degrade extracellular DNA in marine sediments

**DOI:** 10.1038/s41564-021-00936-6

**Published:** 2021-07-19

**Authors:** Kenneth Wasmund, Claus Pelikan, Arno Schintlmeister, Michael Wagner, Margarete Watzka, Andreas Richter, Srijak Bhatnagar, Amy Noel, Casey R. J. Hubert, Thomas Rattei, Thilo Hofmann, Bela Hausmann, Craig W. Herbold, Alexander Loy

**Affiliations:** 1 Division of Microbial Ecology, Centre for Microbiology and Environmental Systems Science, University of Vienna, Vienna, Austria; 2 Austrian Polar Research Institute, Vienna, Austria; 3 Department of Chemistry and Bioscience, Aalborg University, Aalborg, Denmark; 4 Large-Instrument Facility for Environmental and Isotope Mass Spectrometry, Centre for Microbiology and Environmental Systems Science, University of Vienna, Vienna, Austria; 5 Division of Terrestrial Ecosystem Research, Centre for Microbiology and Environmental Systems Science, University of Vienna, Vienna, Austria; 6 Geomicrobiology Group, Department of Biological Sciences, University of Calgary, Calgary, Alberta Canada; 7 Division of Computational Systems Biology, Centre for Microbiology and Environmental Systems Science, University of Vienna, Vienna, Austria; 8 Division of Environmental Geosciences, Centre for Microbiology and Environmental Systems Science, University of Vienna, Vienna, Austria; 9 Joint Microbiome Facility of the Medical University of Vienna and the University of Vienna, Vienna, Austria; 10 Department of Laboratory Medicine, Medical University of Vienna, Vienna, Austria

**Keywords:** Environmental microbiology, Environmental sciences

Correction to: *Nature Microbiology* 10.1038/s41564-021-00917-9, published online 14 June 2021.

This Article was originally published with an incorrect copyright status, and should have been Open Access. This error has now been corrected and the Article is now published under a Creative Commons licence CC BY 4.0.

