# Supplementary material for: Genomic insights into diverse bacterial taxa that degrade extracellular DNA in marine sediments
Source: Nat Microbiol. 2021 Jun 14;6(7):885–98. doi: 10.1038/s41564-021-00917-9 (PMC8289736; doi:10.1038/s41564-021-00917-9)
Supplement: Supplementary file 1 — Supplementary methods, results and discussion; Fig. 1; and Data 1. [file 41564_2021_917_MOESM1_ESM.pdf]

nature portfolio

https://doi.org/10.1038/s41564-021-00917-9

Supplementary information

Genomic insights into diverse bacterial taxa that degrade extracellular DNA in marine sediments

In the format provided by the authors and unedited

Supplementary information for:

Genomic insights into diverse bacterial taxa that degrade extracellular DNA in marine sediments

Kenneth Wasmund[1,2,3,*], Claus Pelikan[1,2], Arno Schintlmeister[1,4], Michael Wagner[1,3,4], Margarete Watzka[5], Andreas Richter[2,5], Srijak Bhatnagar[6], Amy Noel[6], Casey R.J. Hubert[6], Thomas Rattei[7], Thilo Hofmann[8], Bela Hausmann[9,10], Craig W. Herbold[1], Alexander Loy[1,2,9]

[1] Division of Microbial Ecology, Centre for Microbiology and Environmental Systems Science, University of Vienna, Vienna, Austria.
[2]Austrian Polar Research Institute, Vienna, Austria.
[3]Department of Chemistry and Bioscience, Aalborg University, Aalborg, Denmark.
[4]Large-Instrument Facility for Environmental and Isotope Mass Spectrometry, Centre for Microbiology and Environmental Systems Science, University of Vienna, Vienna, Austria.
[5]Division of Terrestrial Ecosystem Research, Centre for Microbiology and Environmental Systems Science,, University of Vienna, Vienna, Austria.
[6]Geomicrobiology Group, Department of Biological Sciences, University of Calgary, Calgary, Canada.
[7]Division of Computational Systems Biology, Centre for Microbiology and Environmental Systems Science, University of Vienna, Vienna, Austria.
[8]Division of Environmental Geosciences, Centre for Microbiology and Environmental Systems Science, University of Vienna, Vienna, Austria.
[9]Joint Microbiome Facility of the Medical University of Vienna and the University of Vienna, Vienna, Austria.
[10]Department of Laboratory Medicine, Medical University of Vienna, Vienna, Austria.

*Correspondence to Kenneth Wasmund: kwasmund@gmail.com

Supplementary Results and Discussion

Microbial community structures in microcosms

The overall bacterial community structures among microcosms shifted over the time course of the experiment, yet were similar between no-substrate control treatments and microcosms supplemented with ^{12}C- or ^{13}C-DNA over time (Extended Data Fig. 3A,B). This indicated that the amounts of added DNA did not significantly alter the overall community dynamics. The communities were generally dominated by the deltaproteobacterial families *Geobacteraceae* and *Desulfuromonadaceae*, which averaged 15% and 11% over all time points, respectively. Other predominant taxa were from the gammaproteobacterial families *Shewanellaceae*, *Colwelliaceae*, *Nitrincolaceae* (GTDB, formerly *Oceanospirillaceae*), and the epsilonprotoebacterial family *Campylobacteraceae*, which averaged 3.5%, 4.7%, 14.5% and 7.5% over all time points, respectively. Members of the families *Flavobacteriaceae*, *Ca*. Izemoplasmataceae (formerly *Tenericutes* family 'NB1-n'), and *Fusibacteraceae* (GTDB, formerly 'Clostridial Family-XII') increased in relative abundances towards the end of the incubations, and made up to 11%, 5% and 14.5% of the sequences at day 31, respectively. The high relative abundances of *Desulfuromonadaceae*, *Geobacteraceae*, *Shewanellaceae*, *Colwelliaceae*, *Campylobacteraceae* and *Oceanospirillaceae* (GTDB name *Nitrincolaceae*) is conspicuously similar to the composition of taxa previously found to dominate in manganese-rich marine sediments from various locations[1–3].

Microbial taxa in microcosms versus in situ sediments

To compare microbial taxa present in the *in situ* sediments (Stations GL-111 and KANE-2b) versus in the microcosms (no-substrate controls only), we compared genera identified among both sets of samples. As described in the Materials and Methods, we used PCR primers targeting 16S rRNA genes of the domain Bacteria to analyse our microcosm and DNA-SIP experiment, in order to avoid co-amplification of added archaeal *Halobacterium* DNA. The in situ sediments were analysed by the University of Calgary laboratory, using universal PCR primers targeting 16S rRNA genes of the domains Bacteria and Archaea. Because the sequences from both datasets do not overlap, they could not be integrated for ASV formation, and we therefore compared taxonomic classifications of sequences from both datasets together, using the same method and database.

This collectively identified a total of 938 genera among both in situ sediments and microcosm datasets, with 24 archaeal genera (excluding *Halobacterium*) and 913 bacterial genera (Supp. Table 1). Archaea were on average 16.3% relative abundance in the in situ samples, and

0.2% of sequences in the microcosms. The most abundant archaeal taxa missing from the microcosms were typical chemolithotrophic ammonia-oxidizers, i.e., *Candidatus* Nitrosopumilus, which was the dominant archaeal genus in situ, making 13.4% relative abundance on average. Therefore, the Bacteria-targeting primers used for analysing the microcosms and DNA-SIP mainly missed low abundance taxa (<0.13% average relative abundance in situ) and archaeal taxa that have specialised roles in oxidizing ammonia, and that are not known to be hydrolysers of organic carbon such as DNA (although this cannot be ruled-out for undescribed taxa) [4]. Among bacterial taxa, 140 genera were detected in situ but were not detected in the microcosms, although the genera not detected in microcosms were only 0.014% relative abundance on average in situ. This was either due to PCR primer bias and/or changes in communities during sediment transport and handling. The bacterial genus *Nannocystis* (Myxococcales) was the most abundant genus not detected in microcosms (excluding 'Bacteria_unclassified'), having 0.13% average relative abundance in the in situ samples (Supp. Table 1). When genera were ranked based on average in situ relative abundance, *Nannocystis* ranked 117 among Bacteria. Overall, this indicated most of the abundant taxa present in situ were also present in the microcosms.

Biogeochemical processes in microcosms

Chemical analyses indicated that sulfate was not depleted over the time course of the incubations (results not shown) and no indications for sulfide production could be detected by smell in any of the microcosms at the end of the experiment. Measurements of total manganese and iron demonstrated high concentrations of these metals in the sediments, i.e., 6.25 and 40.4 g kg^{-1} (dry weight), respectively. This indicated that metals were abundant potential electron acceptors for microorganisms during the anoxic incubations. Together with the high prevalence of typical metal-reducing taxa such as Geobacteraceae and Desulfuromonadaceae (see above), this indicated that the reduction of metals such as manganese and/or iron was likely the predominant terminal respiratory process during this experiment.

Potential caveats of DNA-SIP analysis

As mentioned in the Main section, a potential limitation of our DNA-SIP analysis may have been that determination of ^{13}C-labelling of DNA from low GC-content bacteria may have been limited since gradient densities <1.713 g ml^{-1} were not sequenced. This is because DNA with low GC-content has a lower density than high GC-content DNA, and might be expected to accumulate in gradient densities <1.713 g ml^{-1} [5]. However, taxa of the Fusibacteraceae, *Lutibacter* and *Ca*. Izemoplasma were determined to be ^{13}C-labelled and their genomes have very low GC-contents, i.e., 30-33% GC-content, which showed that our approach still easily detected ^{13}C-labelling of these very low GC-content organisms. Indeed, peaks in relative abundances in

gradients from ^{13}C-treatments for these taxa were determined around 1.73 g ml^{-1} (Extended Data Fig. 6). This was likely because they were highly ^{13}C-labelled, and/or, because DNA typically diffuses across DNA-SIP cesium chloride gradients, as can be inferred by the detection of DNA from these aforementioned low GC-content bacteria even across the dense gradient fractions (>1.725 g ml^{-1}) (Extended Data Fig. 6). Further, it is known that the GC-content of ribosomal RNAs have more median GC-contents (~50-60%) compared to other parts of corresponding genomes [6], possibly suggesting the fragments of genomes containing 16S rRNA genes that were amplified in this study, are still likely to occur across the higher gradient densities analysed in this study. Nevertheless, we acknowledge that some other taxa with low GC-content DNA and with low amounts of ^{13}C-labelling and therefore quantitatively less-important roles in the process, might have been missed in our study.

Catabolic pathways for sub-components of DNA indicate capacity to use DNA-derived carbon and energy

The two different copies of predicted 'purine deoxyribonucleoside phosphorylases' presumably offer specificity to the two purine nucleobases adenine and guanine (Fig. 4). Specific adenosine deaminases were also annotated in all genomes of putative DNA-degraders, except for any of the *Ca*. Izemoplasmataceae.

All putative DNA-degrading taxa encoded cytidine deamidases. Although uracil-specific phosphorylases that are required for further processing of 2-deoxycytidine via 2-deoxyuracil could not be identified, it is possible that the pyrimidine-nucleoside phosphorylases can catalyse both due to their known bifunctionality for these structurally similar substrates[7].

The potential to catabolise the purine bases adenine and guanine were indicated by several lines of evidence. Genes for deaminases specific for both purine bases were present in *Fusibacteraceae* and various *Ca*. Izemoplasmataceae genomes (Fig. 4). In *Lutibacter* spp., adenine deaminase genes could only be identified in two organisms in the NCBI database, while in *Shewanella* spp., only *S. halifaxensis* appeared to harbor this gene. Guanine deaminase genes were also present in numerous *Lutibacter,* and were only identified in one *Shewanella,* i.e., *S. algae*.

Genes for enzymes required for the further catabolism of xanthine could not be unambiguously identified, since most are not described and are only predicted to occur based on biochemical inferences[8]. Nevertheless, several enzymes predicted to have amidohydrolase and hydantoinase activities were also encoded between the xanthine dehydrogenase genes (Extended Data Fig. 9). These possibly play a role in the cleavage of the heterocyclic rings, and thus, further

catabolism of xanthine. Genes for any enzymes involved in purine catabolism were notably absent from any MAGs from the Bacilli family CAG-313 (Fig. 4).

Potential to catabolise the 2-deoxy-D-ribose-phosphate sugar moiety that can be liberated from nucleosides was indicate by the presence of genes for enzymes for catabolism of 2-deoxy-D-ribose-phosphate to D-glyceraldehyde 3-phosphate, an intermediate of glycolysis, and acetaldehyde, in all our MAGs (Fig. 4 and Fig. 5). Genes for aldehyde-alcohol dehydrogenases that could convert acetaldehyde to acetyl-CoA, were present in most labelled groups except in *Lutibacter*, and were only present in some marine *Ca*. Izemoplasmataceae MAGs, i.e., *Ca*. Izemoplasma sp. ZiA1 and HR1. Genes for a protein microcompartment that was postulated to be used for catabolising the liberated aldehydes in *Ca*. Izemoplasma sp. HR2[9], were not found in any other *Ca*. Izemoplasma MAGs analysed here.

Distinct responses of diverse bacteria to additions of DNA, nucleobases or nucleosides

Among microcosms where different nucleosides and nucleobases were amended, only 2 ASVs responded to thymine, while 14 different ASVs responded to thymidine. Interestingly, although no Fusibacteraceae ASVs became enriched in microcosms in response to additions of DNA only, numerous ASVs (n=13) from this group were enriched in response to purine-based nucleobases and/or nucleosides, especially those containing adenine moieties. Three of these Fusibacteraceae ASVs were determined to be ^{13}C-labelled in the DNA-SIP experiment. Fusibacteraceae ASVs were the only taxa enriched by 2-deoxyadenosine, whereby 13 different ASVs responded. For instance, Fusibacteraceae ASV 05827 dominated communities amended with 2-deoxyadenosine at day 10, reaching approximately 31% in relative abundance, versus 0.6% in the no-substrate controls (Supp. Data 1). Six of the Fusibacteraceae ASVs that responded to 2-deoxyadenosine also responded to adenine. These ASVs also showed higher responses to the sugar-containing nucleoside 2-deoxyadenosine than the corresponding nucleobase adenine. Apart from taxa that were also labelled in the SIP analyses, several ASVs of the family Endozoicomonadaceae (class Gammaproteobacteria) were noticeably enriched in a number of treatments in this study, i.e., by DNA, adenine, guanine and thymidine (Fig. 1 and Supp. Data 1).

These analyses showed that additions of different individual sub-components of DNA to microcosms invoked highly distinct shifts in 16S rRNA gene relative abundances of diverse bacterial taxa. Interestingly, the different responses also occurred among distinct yet related members of some phylogenetic clades. For instance, various ASVs from the Fusibacteraceae responded differently to several DNA sub-components, especially purine-based molecules. This shows how different sub-components of DNA have the potential to be partitioned among different

microorganisms. Free DNA subcomponents could arise if they are liberated from the parent molecule, e.g., after extracellular digestion of DNA by nucleases excreted by other primary DNA-degraders. This may therefore open-up multiple niches for different taxa, and in doing so, promote microbial diversity. In relation to this, additions of purine-based molecules consistently elucidated greater responses and from more diverse taxa, compared to responses to pyrimidine-based molecules. This aligns with previous research in estuarine waters, which showed that purine additions to water samples elicited considerably stronger end-product (urea) production than pyrimidines[10]. Further, we noted that more ASVs responded to nucleosides than their respective nucleobases. This signifies that the sugar moiety was preferentially exploited by some bacteria for energy conservation, rather than the base. This was especially notable for the pyrimidine nucleoside thymidine, which stimulated significant responses from fourteen different ASVs, in comparison thymine itself that elucidated responses from only two ASVs.

Fusibacteraceae are efficient degraders of nucleosides and nucleobases

In the microcosms experiment, various ASVs of the Fusibacteraceae showed strong responses to additions of the various nucleosides and nucleobases. They also did not increase in relative abundances when DNA was added as substrate. Although unknown extracellular nucleases may exist, their absence was notable because most previously described extracellular nucleases of anaerobic bacteria are derived from related Firmicutes, such as *Bacillus* and *Staphylococcus* species[11,12]. This led us to infer that the Fusibacteraceae may indeed lack extracellular nucleases, although future research would be needed to clarify this. On the otherhand, genes for multiple predicted cell wall 5'-nucleotidases highlighted their potential to use nucleosides and nucleobases. Further, in-line with observations from the microcosm experiments that showed strong responses of Fusibacteraceae to 2-deoxyadenosine amendments in the microcosms, the *Fusibacter* sp. 3D3 reference genome harboured multiple copies of adenosine deaminase genes. This may have enabled extra production of this enzyme and therefore more efficient processing of these molecules to the sugar and base moieties, which could then be further catabolised. This may therefore explain their strong increases in relative abundances to additions of these molecules.

DNA-degrading taxa identified by SIP are globally distributed in marine sediments

To examine the global distributions of the four DNA-degrading bacterial genera/species identified in the SIP analyses, we examined the presence of 16S rRNA gene sequences closely related to ASVs that were determined to be labelled in the SIP analyses (Fig. 1) in publically available Short Read Archive (SRA) datasets. We identified 247 SRA samples from marine sediments that simultaneously contained sequences from all of the five genera/species. The relative abundances of these taxa in representative samples of each of the sites (n=18) showed that these

taxa are globally distributed (Extended Data Fig. 8). The samples included diverse marine ecosystems, from both coastal to deep-sea environments, as well as polar and tropical marine environments. They also spanned distant locations, from both Arctic and Antarctic regions, as well as from European, Asian and North and South American continental margins. At the most, these five taxa collectively represented 10% of some microbial communities in methane seep associated sediments off the coast of Oregon, USA. Sequences related to *Ca*. Izemoplasma ASV_06088 were found up to 2% in river delta sediments of the Adriatic Sea, Italy.

Description of relatedness of MAGs to known organisms and genomes

Here we provide a description of the phylogenetic affiliations and novelty of MAGs recovered in this study revealed by phylogenomic analysis (Fig. 3), genome based average nucleotide identity (ANI) analyses (Supp. Table 4), as well as in relation to 16S rRNA genes recovered from the same samples. Taxonomic cut-offs based on genome ANI[13] and 16S rRNA sequence identities[14] were considered. The *Lutibacter* MAG BB-2 was phylogenetically affiliated with the genus *Lutibacter*, yet could be considered a novel species based on an ANI of 78% to *Lutibacter flavis*, and the fact that no recovered 16S rRNA genes had >98% identity to any *Lutibacter* spp.. The *Shewanella* MAG BB-1 was phylogenetically affiliated within the genus *Shewanella* and most related to *Shewanella sediminis.* Nevertheless, it probably constituted a novel species based on 86.5% ANI from 62% of aligned genome to *Shewanella sediminis*. The *Fusibacteraceae* MAG BB-3 was broadly related to *Fusibacter* sp. 3D3 based of phylogenomic analysis, had only 65.2% ANI from only 16% aligned, and together with the fact that 16S rRNA gene OTUs from this family had <94% identity to any cultured species, it may thus represent a novel genus. The *Ca.* Izemoplasma acidinucleici MAG was most related to yet clearly distinct from *Ca.* Izemoplasma sp. HR1, with 70.5% genome ANI from only 42.9% aligned. The assembled 16S rRNA gene of *Ca.* Izemoplasma MAG had only 94% sequence identity to *Ca.* Izemoplasma sp. HR1. This MAG therefore represents a novel species for which we propose the name *Ca.* Izemoplasma acidinucleici.

Genes for de novo biosynthesis of nucleotides in *Ca*. Izemoplasmataceae genomes

Among *Ca*. Izemoplasma, all genes except one required for *de novo* syntheses of purines could be identified (Supp. Table 6). Only a nucleoside diphosphate kinase required for phosphorylation of GDP to GTP was not encoded. Nevertheless, this pathway must be performed by an alternative means otherwise the organisms would not be viable. Instead, it may be performed by a predicted multi-functional adenylate kinase, as was previously predicted to perform this function in related phytoplasmas[15]. Similarly, all genes required for complete *de novo* pyrimidine biosyntheses from glutamine and bicarbonate could be identified among *Ca*. Izemoplasma except

for a canonical carbamoyl phosphate synthetase, which was only present in *Ca*. Izemoplasma acidinucleici (Supp. Table 6). Instead, we identified genes for carbamate kinases in most *Ca*. Izemoplasma that may instead catalyze this step, as was shown previously for various archaea[16].

Large protein with DNA-binding and nuclease domains among *Ca*. Izemoplasmatales

Encoded within the 'DNA-degradation loci' of various *Ca*. Izemoplasmatales (Fig. 6) was a 'Large protein with nucleic acid-binding residues, C-terminal endonuclease I domain and 'Lamin Tail Domain'. These domains were detected by BLASTP against the NCBI's Conserved Domain Database (CDD)[17] (Supp. information Fig. 1). Details regarding these domains include:

- 'Generic binding surface' domain:
    - Mostly consists of nucleic acid substrates in most OB-fold complexes[16].
- YhcR_OBF_like domainv (RPA_2b):
    - Subfamily of OB-fold domains *Bacillus subtilis* YhcR. The YhcR consists of a sugar-nonspecific nuclease[17].
- 'Lamin Tail Domain' (LTD):
    - In some secreted or periplasmic proteins, LTDs are associated with other substrate-binding domains such as those for oligosaccharides. These associations indicate possible roles for LTDs to act as tethering proteins to membranes or associated protein[18].

We hypothesize that this large protein could function to hold polymeric DNA in close proximity to the cell during digestion, since it contains the domains necessary to do so. In that way, DNA could be degraded in concert with the other secreted nucleases close to the cell, thereby minimizing diffusion of liberated sub-components and enable efficient uptake by the associated transporters.

Proposal of the novel species *Ca*. Izemoplasma acidinucleici and amendment of the genus name *Ca*. Izimaplasma to *Ca*. Izemoplasma

Based on a combination of unique phylogeny (based on 16S rRNA genes and concatenated single copy marker proteins) and genomic similarity (ANI), as well as genome-based predictions of nucleic acid catabolism that are supported by SIP results, we propose a novel species name *Candidatus* Izemoplasma acidinucleici for the *Ca*. Izemoplasma MAG recovered in this study. We have revised the previously suggested genus name *Ca*. Izimaplasma[9] to *Candidatus* Izemoplasma of the family *Izemoplasmataceae* and order *Izemoplasmatales*. We propose the MAG of *Candidatus* Izemoplasma acidinucleici as type material for this lineage, since it fulfils the

properties outlined by Chuvochina *et. al.*[19], with the only exception being that it is contained within 191 contigs.

- *Ca*. Izemoplasma gen. nov. (I.ze.mo.plas'ma. Gr. n. Izema, that what settles, sediment; - o-, connecting vowel; Gr. neut. n. plasma, something that forms, N.L. neut. n. Izemoplasma, a formed structure from sediment) with *Ca*. Izemoplasma acidinucleici (a.ci.di.nu.cle'i.ci. N.L. n. acidum nucleicu m, nucleic acid; N.L. gen. n. acidinucleici, of nucleic acid, referring to).

References for Supporting Information

1. Cho, H. *et al.* Acetate-utilizing microbial communities revealed by stable-isotope probing in sediment underlying the upwelling system of the Ulleung Basin, East Sea. *Mar. Ecol. Prog. Ser.* **634**, 45–61 (2020).
2. Vandieken, V. *et al.* Three manganese oxide-rich marine sediments harbor similar communities of acetate-oxidizing manganese-reducing bacteria. *ISME J.* **6**, 2078–2090 (2012).
3. Buongiorno, J. *et al.* Complex Microbial Communities Drive Iron and Sulfur Cycling in Arctic Fjord Sediments. *Appl. Environ. Microbiol.* **85**, e00949-19 (2019).
4. Qin, W. *et al. Candidatus* Nitrosocaldales. *Bergey's Manual of Systematics of Archaea and Bacteria* 1–2 (2017) doi:10.1002/9781118960608.obm00120.
5. Buckley, D. H., Huangyutitham, V., Hsu, S.-F. & Nelson, T. A. Stable isotope probing with ^{15}N achieved by disentangling the effects of genome G+C content and isotope enrichment on DNA density. *Appl. Environ. Microbiol.* **73**, 3189–3195 (2007).
6. Lueders, T. Stable Isotope Probing of Hydrocarbon-Degraders. in *Handbook of Hydrocarbon and Lipid Microbiology*. (eds Timmis K. N.) (Springer, Berlin, Heidelberg. 2010).
7. Leer, J. C., Hammer-Jespersen, K. & Schwartz, M. Uridine phosphorylase from *Escherichia coli*. Physical and chemical characterization. *Eur. J. Biochem.* **75**, 217–224 (1977).
8. Hartwich, K., Poehlein, A. & Daniel, R. The purine-utilizing bacterium *Clostridium acidurici* 9a: a genome-guided metabolic reconsideration. *PLoS One* **7**, e51662 (2012).
9. Skennerton, C. T. *et al.* Phylogenomic analysis of *Candidatus* 'Izimaplasma'species: free-living representatives from a Tenericutes clade found in methane seeps. *ISME J.* **10**, 2679–2692 (2016).
10. Berg, G. M. & Jørgensen, N. O. G. Purine and pyrimidine metabolism by estuarine bacteria. *Aquat. Microb. Ecol.* **42**, 215–226 (2006).
11. Parks, D. H. *et al.* A standardized bacterial taxonomy based on genome phylogeny substantially revises the tree of life. *Nat. Biotechnol.* **36**, 996–1004 (2018).
12. Pediaditakis, M. & Kaufenstein, M. *Bacillus subtilis hlpB* Encodes a Conserved Stand-Alone HNH Nuclease-Like Protein That Is Essential for Viability Unless the *hlpB* Deletion Is Accompanied by the Deletion of Genes Encoding the AddAB DNA Repair Complex. *J. Bacteriol.* **194**, 6184–6194 (2012).

13. Konstantinidis, K. T., Rosselló-Móra, R. & Amann, R. Uncultivated microbes in need of their own taxonomy. *ISME J.* **11**, 2399–2406 (2017).
14. Yarza, P. *et al.* Uniting the classification of cultured and uncultured bacteria and archaea using 16S rRNA gene sequences. *Nat. Rev. Microbiol.* **12**, 635–645 (2014).
15. Kube, M., Mitrovic, J., Duduk, B., Rabus, R. & Seemüller, E. Current view on phytoplasma genomes and encoded metabolism. *Sci. World J.* **2012**, 185942 (2012).
16. Alcántara, C., Cervera, J. & Rubio, V. Carbamate kinase can replace in vivo carbamoyl phosphate synthetase. Implications for the evolution of carbamoyl phosphate biosynthesis. *FEBS Lett.* **484**, 261–264 (2000).
17. Marchler-Bauer, A. *et al.* CDD/SPARCLE: functional classification of proteins via subfamily domain architectures. *Nucleic Acids Res.* **45**, D200–D203 (2017).
18. Mitchell, A. L. *et al.* InterPro in 2019: improving coverage, classification and access to protein sequence annotations. *Nucleic Acids Res.* **47**, D351–D360 (2019).
19. Chuvochina, M. *et al.* The importance of designating type material for uncultured taxa. *Syst. Appl. Microbiol.* **42**, 15–21 (2019).

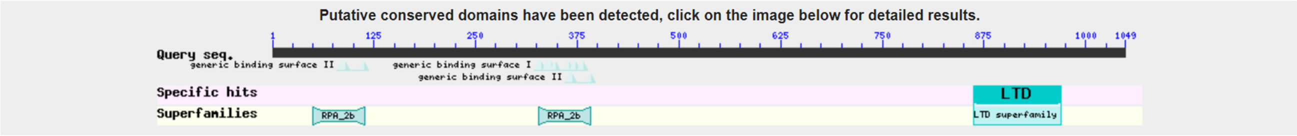

Supp. Information Fig. 1. Result of BLASTP analysis of peg.646 (Supp. Table 5) from *Candidatus* Izemoplasma acidinucleici against the Conserved Domain Database (CDD).

Supplementary Data 1. Box plots of 16S rRNA gene relative abundances of ASVs that were significantly enriched (*p* value <0.05) in a treatment versus the no-substrate control from the same time points. Only ASVs that were significantly enriched at ≥2 time points are presented. Red highlighted treatments indicate which treatments were significantly enriched compared to their corresponding no-substrate controls, at ≥2 time points. The 'Serial Group Comparisons' pipeline of Rhea was applied using data from triplicate microcosms, using Wilcoxon Signed Rank Sum Test (two-sided; non-parametric; no adjustments for multiple comparisons). All inputs and outputs (including exact *p* values) from the Rhea statistical analysis pipeline are available to download from: https://doi.org/10.6084/m9.figshare.14398853.v1. Treatments for nucleobases and nucleosides were only sequenced from the first three time points, i.e., days 4, 10 and 13. Taxonomic strings displayed = domain;Phylum;Class;Order;Family; Genus; and are derived from the classifications against SILVA 16S rRNA gene database (see Materials and Methods). Further taxonomic information in relation to the Genome Taxonomic Database (GTDB) can be interpreted from Fig. 1. In box plots: center lines show the medians; box limits indicate the 25th and 75th percentiles as determined by R software; whiskers extend 1.5 times the interquartile range from the 25th and 75th percentiles; data points are plotted as open circles.

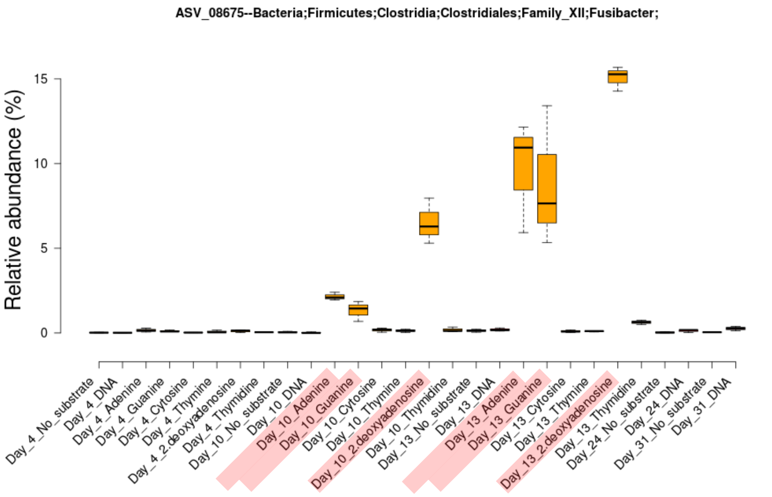
ASV_08675--Bacteria;Firmicutes;Clostridia;Clostridiales;Family_XII;Fusibacter;
Relative abundance (%)
0
5
10
15
Day_4_No_substrate
Day_4_DNA
Day_4_Adenine
Day_4_Guanine
Day_4_Cytosine
Day_4_Thymine
Day_4_2deoxyadenosine
Day_4_Thymidine
Day_10_No_substrate
Day_10_DNA
Day_10_Adenine
Day_10_Guanine
Day_10_Cytosine
Day_10_Thymine
Day_10_2deoxyadenosine
Day_10_Thymidine
Day_13_No_substrate
Day_13_DNA
Day_13_Adenine
Day_13_Guanine
Day_13_Cytosine
Day_13_Thymine
Day_13_2deoxyadenosine
Day_13_Thymidine
Day_24_No_substrate
Day_24_DNA
Day_31_No_substrate
Day_31_DNA

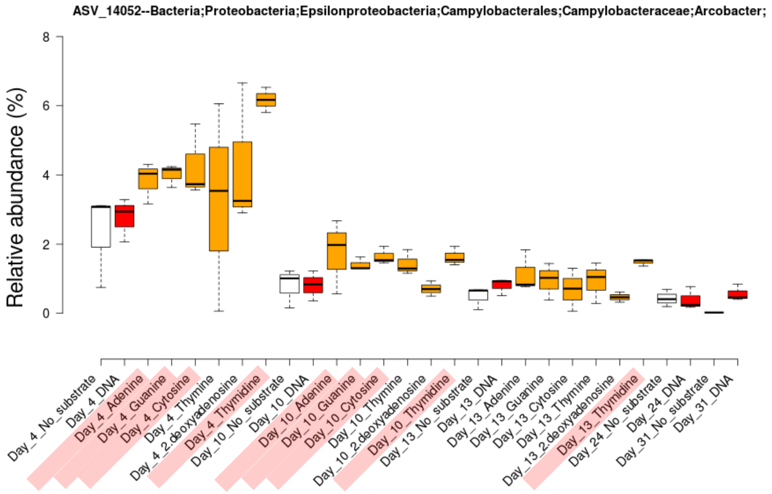
ASV_14052--Bacteria;Proteobacteria;Epsilonproteobacteria;Campylobacterales;Campylobacteraceae;Arcobacter;
Relative abundance (%)
0
2
4
6
8
Day_4_No_substrate
Day_4_DNA
Day_4_Adenine
Day_4_Guanine
Day_4_Cytosine
Day_4_Thymine
Day_4_2deoxyadenosine
Day_4_Thymidine
Day_10_No_substrate
Day_10_DNA
Day_10_Adenine
Day_10_Guanine
Day_10_Cytosine
Day_10_Thymine
Day_10_2deoxyadenosine
Day_10_Thymidine
Day_13_No_substrate
Day_13_DNA
Day_13_Adenine
Day_13_Guanine
Day_13_Cytosine
Day_13_Thymine
Day_13_2deoxyadenosine
Day_13_Thymidine
Day_24_No_substrate
Day_24_DNA
Day_31_No_substrate
Day_31_DNA

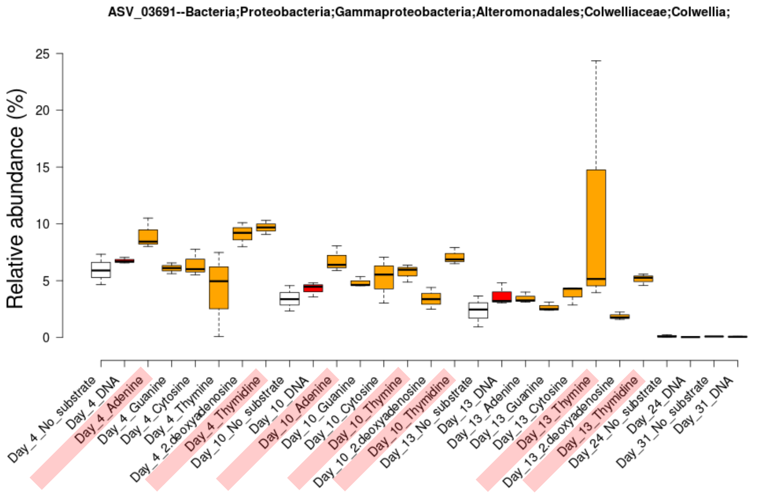
ASV_03691--Bacteria;Proteobacteria;Gammaproteobacteria;Alteromonadales;Colwelliaceae;Colwellia;
Relative abundance (%)
0
5
10
15
20
25
Day_4_No_substrate
Day_4_DNA
Day_4_Adenine
Day_4_Guanine
Day_4_Cytosine
Day_4_Thymine
Day_4_2deoxyadenosine
Day_4_Thymidine
Day_10_No_substrate
Day_10_DNA
Day_10_Adenine
Day_10_Guanine
Day_10_Cytosine
Day_10_Thymine
Day_10_2deoxyadenosine
Day_10_Thymidine
Day_13_No_substrate
Day_13_DNA
Day_13_Adenine
Day_13_Guanine
Day_13_Cytosine
Day_13_Thymine
Day_13_2deoxyadenosine
Day_13_Thymidine
Day_24_No_substrate
Day_24_DNA
Day_31_No_substrate
Day_31_DNA

ASV_05827--Bacteria;Firmicutes;Clostridia;Clostridiales;Family_XII;Fusibacter;

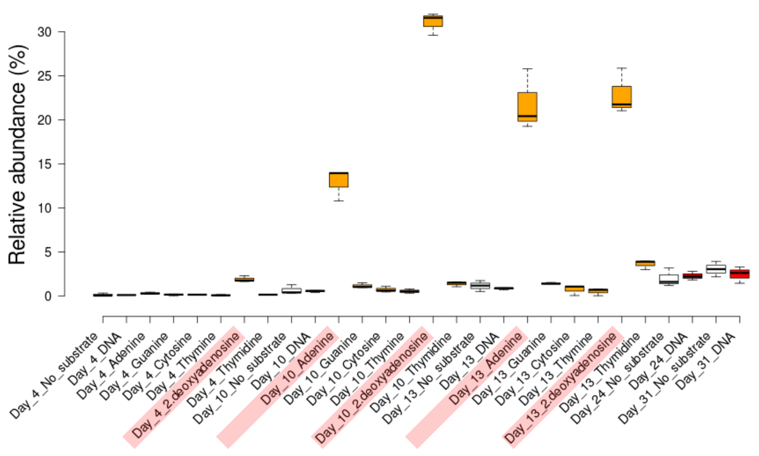

ASV_06881--Bacteria;Firmicutes;Clostridia;Clostridiales;Family_XII;Fusibacter;

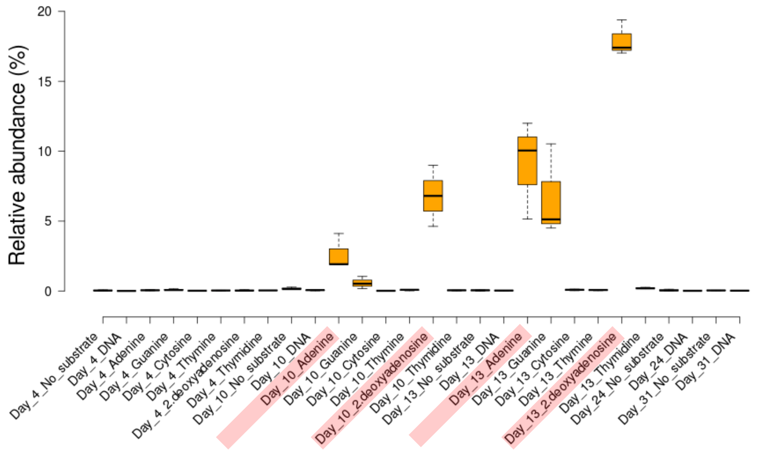

ASV_12207--Bacteria;Firmicutes;Clostridia;Clostridiales;Family_XII;Fusibacter;

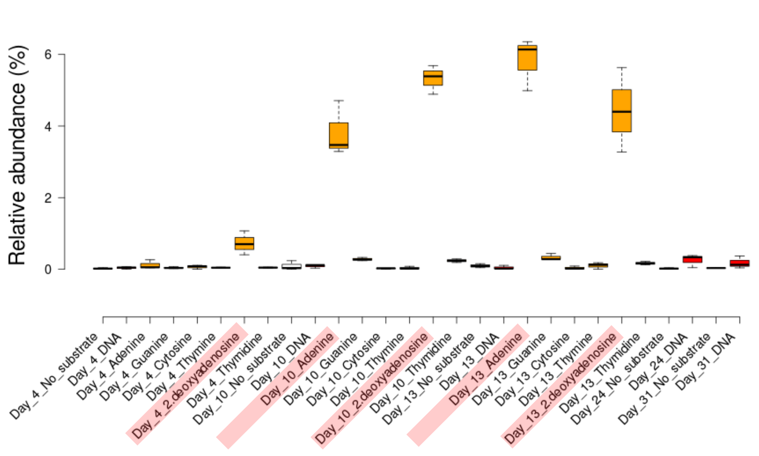

ASV_04154--Bacteria;Proteobacteria;Gammaproteobacteria;Oceanospirillales;Oceanospirillales_unclassified;Oceanospirillales_uncla

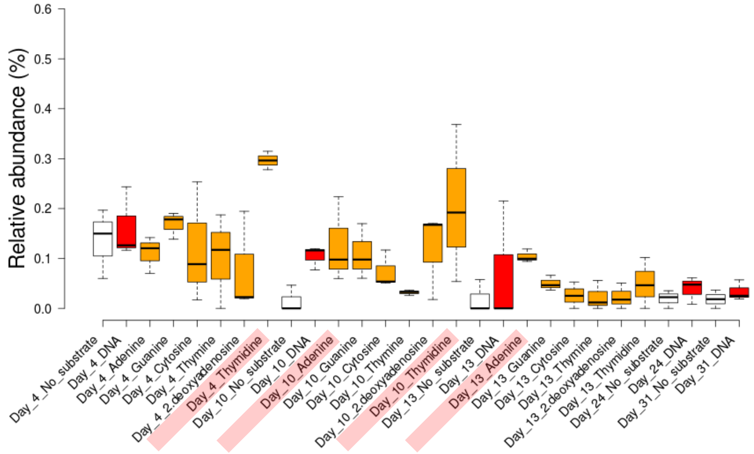

ASV_05145--Bacteria;Proteobacteria;Gammaproteobacteria;Alteromonadales;Colwelliaceae;Colwelliaceae_unclassified;

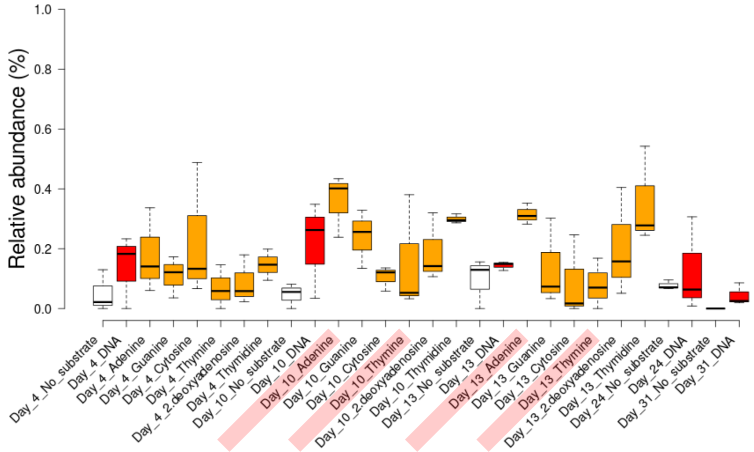

ASV_06164--Bacteria;Firmicutes;Clostridia;Clostridiales;Family_XII;Fusibacter;

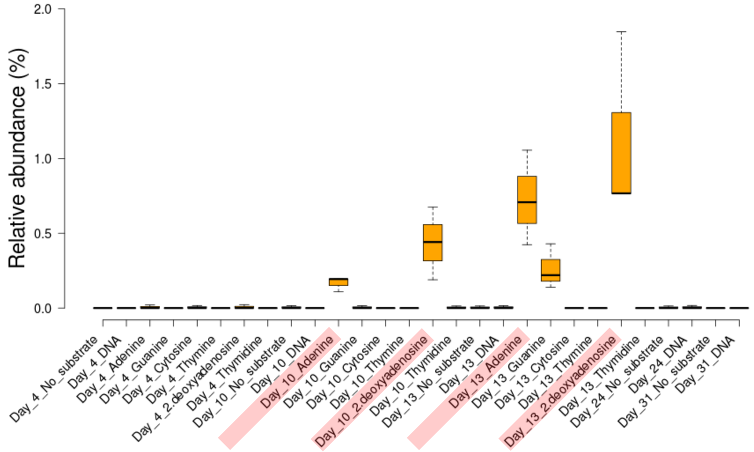

ASV_07899--Bacteria;Proteobacteria;Deltaproteobacteria;Desulfuromonadales;Desulfuromonadales_unclassified;Desulfuromonadales_u

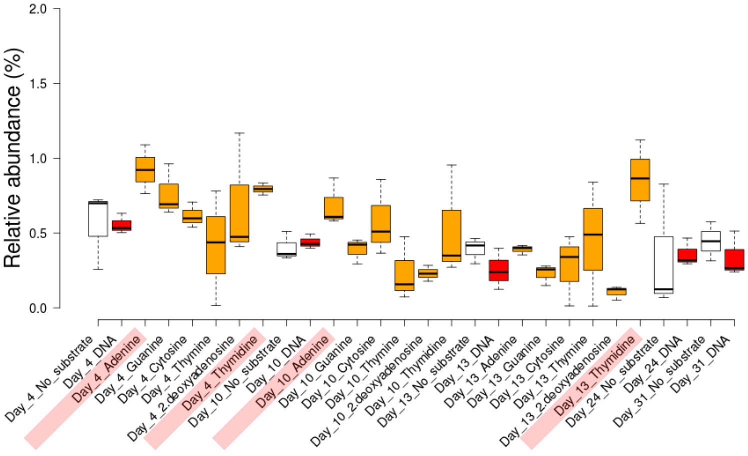

ASV_08637--Bacteria;Proteobacteria;Gammaproteobacteria;Oceanospirillales;Oceanospirillaceae;Neptunomonas;

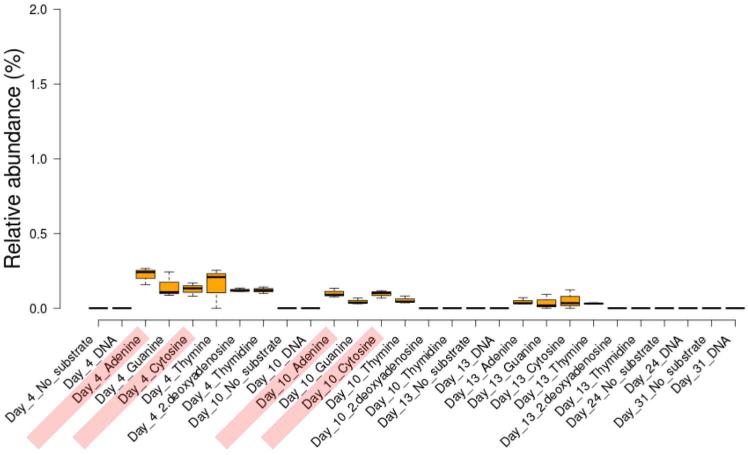

ASV_09337--Bacteria;Proteobacteria;Gammaproteobacteria;Oceanospirillales;Hahellaceae;Endozoicomonas;

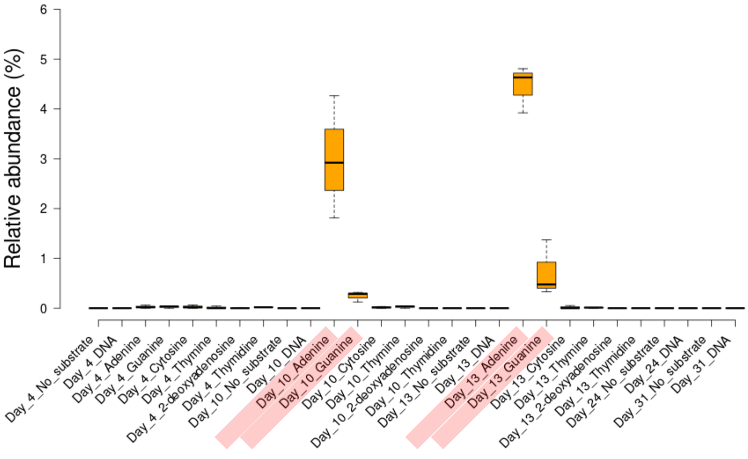

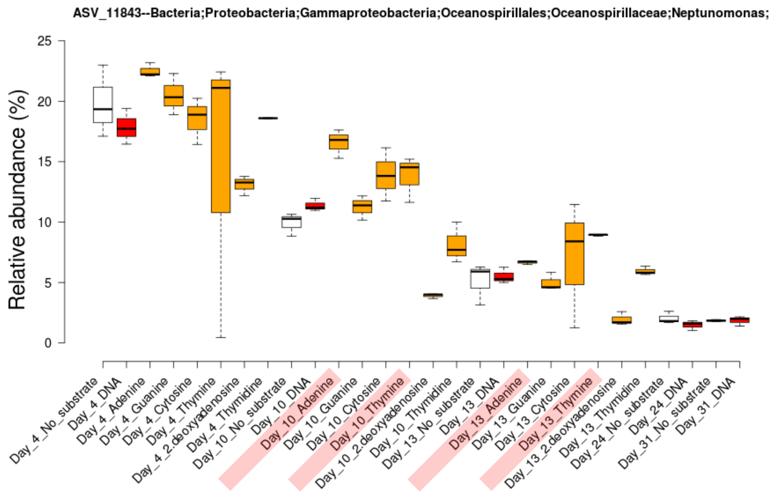
ASV_11843--Bacteria;Proteobacteria;Gammaproteobacteria;Oceanospirillales;Oceanospirillaceae;Neptunomonas;
Relative abundance (%)
Day_4_No_substrate
Day_4_DNA
Day_4_Adenine
Day_4_Guanine
Day_4_Cytosine
Day_4_Thymine
Day_4_2.deoxyadenosine
Day_4_Thymidine
Day_10_No_substrate
Day_10_DNA
Day_10_Adenine
Day_10_Guanine
Day_10_Cytosine
Day_10_Thymine
Day_10_2.deoxyadenosine
Day_10_Thymidine
Day_13_No_substrate
Day_13_DNA
Day_13_Adenine
Day_13_Guanine
Day_13_Cytosine
Day_13_Thymine
Day_13_2.deoxyadenosine
Day_13_Thymidine
Day_24_No_substrate
Day_24_DNA
Day_31_No_substrate
Day_31_DNA

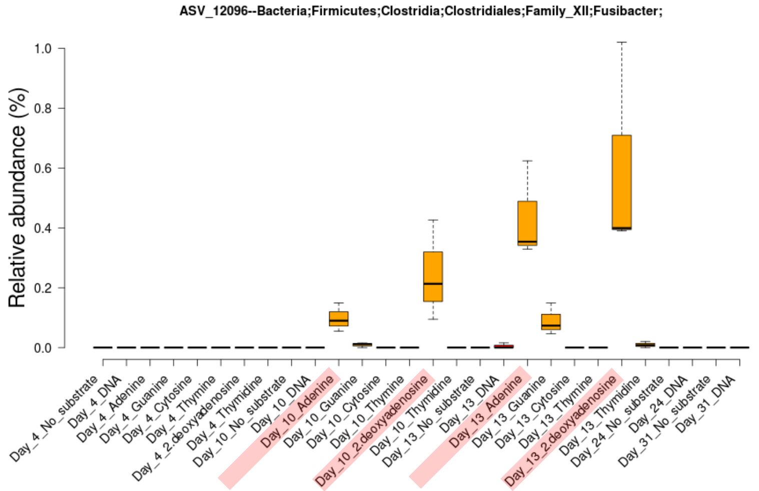
ASV_12096--Bacteria;Firmicutes;Clostridia;Clostridiales;Family_XII;Fusibacter;
Relative abundance (%)

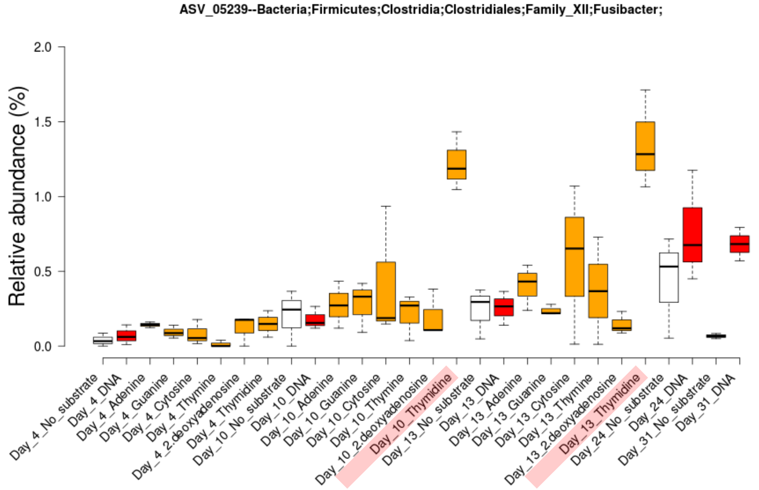
ASV_05239--Bacteria;Firmicutes;Clostridia;Clostridiales;Family_XII;Fusibacter;
Relative abundance (%)

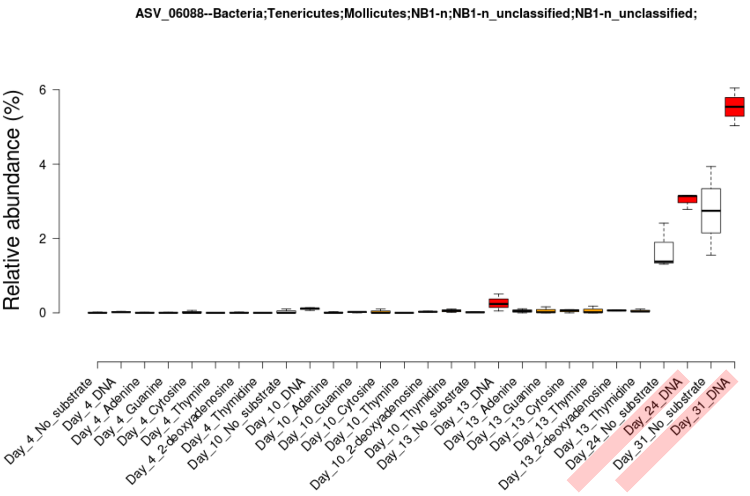
ASV_06088--Bacteria;Tenericutes;Mollicutes;NB1-n;NB1-n_unclassified;NB1-n_unclassified;
Relative abundance (%)
0
2
4
6
Day_4_No_substrate
Day_4_DNA
Day_4_Adenine
Day_4_Guanine
Day_4_Cytosine
Day_4_Thymine
Day_4_2-deoxyadenosine
Day_4_Thymidine
Day_10_No_substrate
Day_10_DNA
Day_10_Adenine
Day_10_Guanine
Day_10_Cytosine
Day_10_Thymine
Day_10_2-deoxyadenosine
Day_10_Thymidine
Day_13_No_substrate
Day_13_DNA
Day_13_Adenine
Day_13_Guanine
Day_13_Cytosine
Day_13_Thymine
Day_13_2-deoxyadenosine
Day_13_Thymidine
Day_24_No_substrate
Day_24_DNA
Day_31_No_substrate
Day_31_DNA

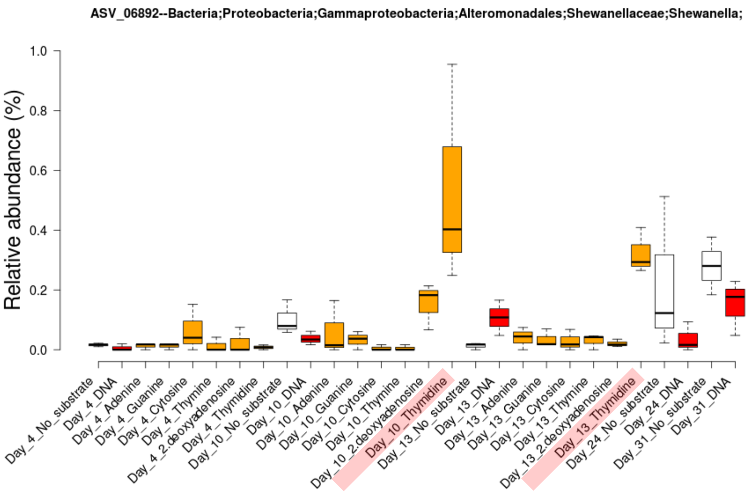
ASV_06892--Bacteria;Proteobacteria;Gammaproteobacteria;Alteromonadales;Shewanellaceae;Shewanella;
Relative abundance (%)
0.0
0.2
0.4
0.6
0.8
1.0
Day_4_No_substrate
Day_4_DNA
Day_4_Adenine
Day_4_Guanine
Day_4_Cytosine
Day_4_Thymine
Day_4_2-deoxyadenosine
Day_4_Thymidine
Day_10_No_substrate
Day_10_DNA
Day_10_Adenine
Day_10_Guanine
Day_10_Cytosine
Day_10_Thymine
Day_10_2-deoxyadenosine
Day_10_Thymidine
Day_13_No_substrate
Day_13_DNA
Day_13_Adenine
Day_13_Guanine
Day_13_Cytosine
Day_13_Thymine
Day_13_2-deoxyadenosine
Day_13_Thymidine
Day_24_No_substrate
Day_24_DNA
Day_31_No_substrate
Day_31_DNA

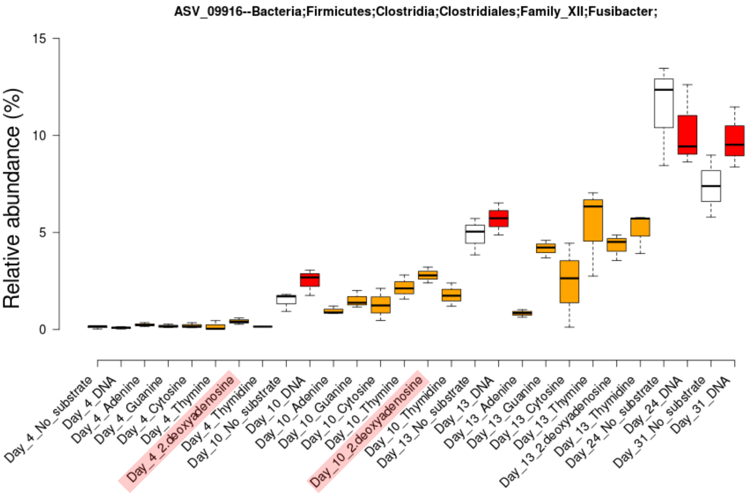
ASV_09916--Bacteria;Firmicutes;Clostridia;Clostridiales;Family_XII;Fusibacter;
Relative abundance (%)
0
5
10
15
Day_4_No_substrate
Day_4_DNA
Day_4_Adenine
Day_4_Guanine
Day_4_Cytosine
Day_4_Thymine
Day_4_2-deoxyadenosine
Day_4_Thymidine
Day_10_No_substrate
Day_10_DNA
Day_10_Adenine
Day_10_Guanine
Day_10_Cytosine
Day_10_Thymine
Day_10_2-deoxyadenosine
Day_10_Thymidine
Day_13_No_substrate
Day_13_DNA
Day_13_Adenine
Day_13_Guanine
Day_13_Cytosine
Day_13_Thymine
Day_13_2-deoxyadenosine
Day_13_Thymidine
Day_24_No_substrate
Day_24_DNA
Day_31_No_substrate
Day_31_DNA

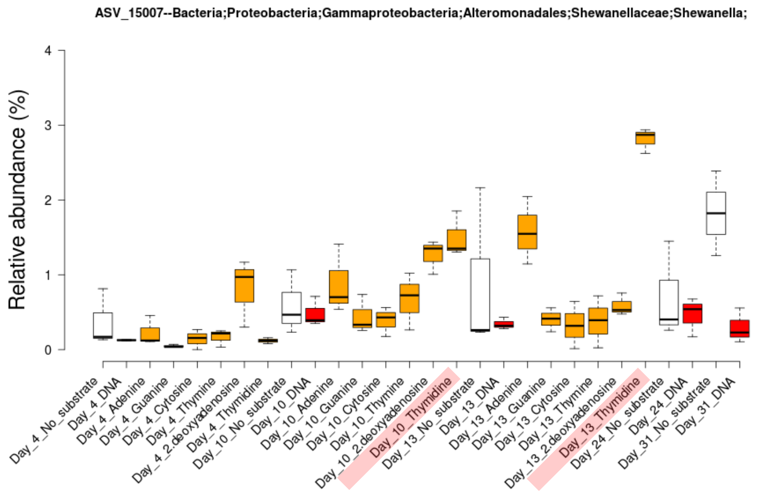
ASV_15007--Bacteria;Proteobacteria;Gammaproteobacteria;Alteromonadales;Shewanellaceae;Shewanella;
Relative abundance (%)
0
1
2
3
4
Day_4_No_substrate
Day_4_DNA
Day_4_Adenine
Day_4_Guanine
Day_4_Cytosine
Day_4_Thymine
Day_4_2.deoxyadenosine
Day_4_Thymidine
Day_10_No_substrate
Day_10_DNA
Day_10_Adenine
Day_10_Guanine
Day_10_Cytosine
Day_10_Thymine
Day_10_2.deoxyadenosine
Day_10_Thymidine
Day_13_No_substrate
Day_13_DNA
Day_13_Adenine
Day_13_Guanine
Day_13_Cytosine
Day_13_Thymine
Day_13_2.deoxyadenosine
Day_13_Thymidine
Day_24_No_substrate
Day_24_DNA
Day_31_No_substrate
Day_31_DNA

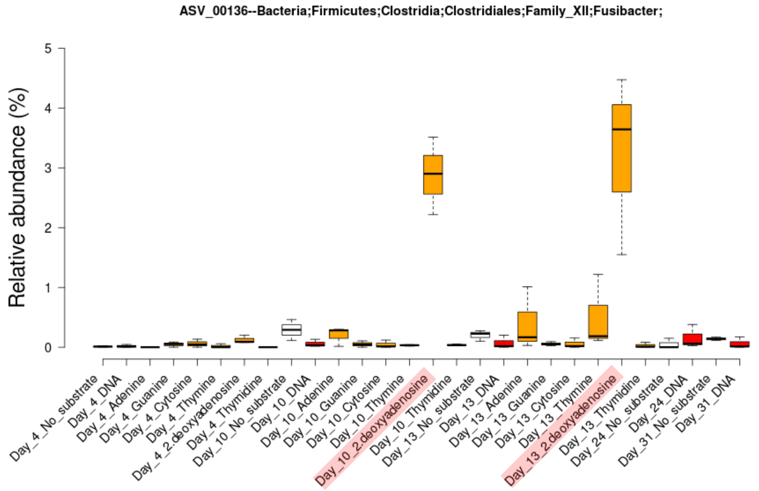
ASV_00136--Bacteria;Firmicutes;Clostridia;Clostridiales;Family_XII;Fusibacter;
Relative abundance (%)
0
1
2
3
4
5
Day_4_No_substrate
Day_4_DNA
Day_4_Adenine
Day_4_Guanine
Day_4_Cytosine
Day_4_Thymine
Day_4_2.deoxyadenosine
Day_4_Thymidine
Day_10_No_substrate
Day_10_DNA
Day_10_Adenine
Day_10_Guanine
Day_10_Cytosine
Day_10_Thymine
Day_10_2.deoxyadenosine
Day_10_Thymidine
Day_13_No_substrate
Day_13_DNA
Day_13_Adenine
Day_13_Guanine
Day_13_Cytosine
Day_13_Thymine
Day_13_2.deoxyadenosine
Day_13_Thymidine
Day_24_No_substrate
Day_24_DNA
Day_31_No_substrate
Day_31_DNA

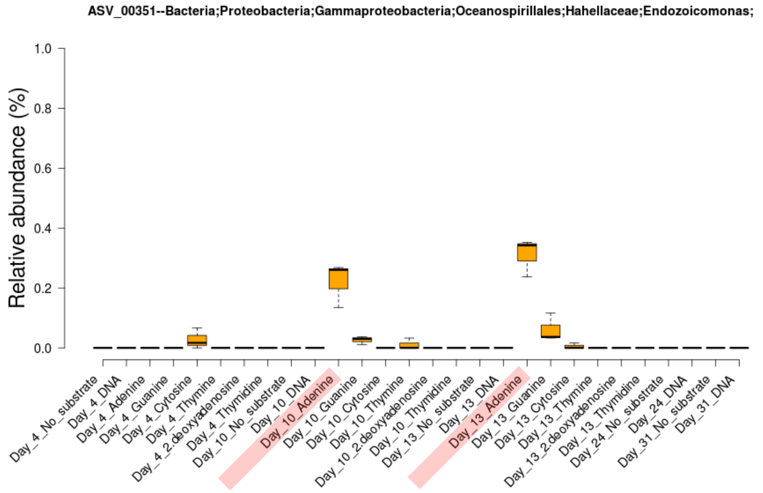
ASV_00351--Bacteria;Proteobacteria;Gammaproteobacteria;Oceanospirillales;Hahellaceae;Endozoicomonas;
Relative abundance (%)
0.0
0.2
0.4
0.6
0.8
1.0
Day_4_No_substrate
Day_4_DNA
Day_4_Adenine
Day_4_Guanine
Day_4_Cytosine
Day_4_Thymine
Day_4_2.deoxyadenosine
Day_4_Thymidine
Day_10_No_substrate
Day_10_DNA
Day_10_Adenine
Day_10_Guanine
Day_10_Cytosine
Day_10_Thymine
Day_10_2.deoxyadenosine
Day_10_Thymidine
Day_13_No_substrate
Day_13_DNA
Day_13_Adenine
Day_13_Guanine
Day_13_Cytosine
Day_13_Thymine
Day_13_2.deoxyadenosine
Day_13_Thymidine
Day_24_No_substrate
Day_24_DNA
Day_31_No_substrate
Day_31_DNA

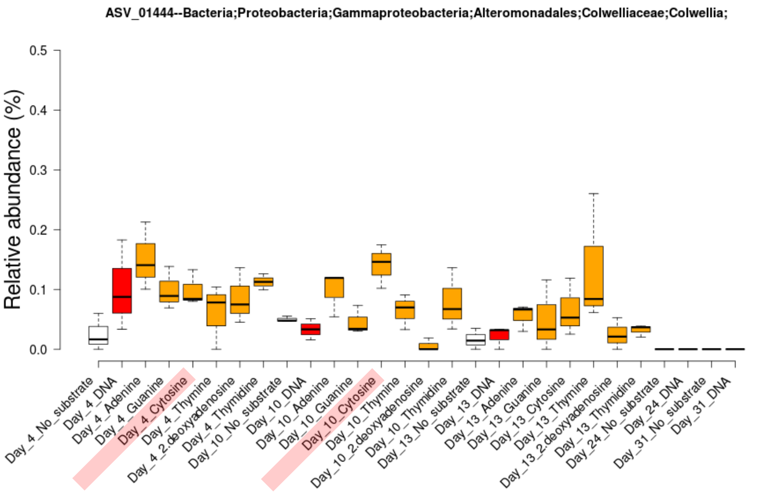
ASV_01444--Bacteria;Proteobacteria;Gammaproteobacteria;Alteromonadales;Colwelliaceae;Colwellia;
Relative abundance (%)
0.0
0.1
0.2
0.3
0.4
0.5
Day_4_No_substrate
Day_4_DNA
Day_4_Adenine
Day_4_Guanine
Day_4_Cytosine
Day_4_Thymine
Day_4_2deoxyadenosine
Day_4_Thymidine
Day_10_No_substrate
Day_10_DNA
Day_10_Adenine
Day_10_Guanine
Day_10_Cytosine
Day_10_Thymine
Day_10_2deoxyadenosine
Day_10_Thymidine
Day_13_No_substrate
Day_13_DNA
Day_13_Adenine
Day_13_Guanine
Day_13_Cytosine
Day_13_Thymine
Day_13_2deoxyadenosine
Day_13_Thymidine
Day_24_No_substrate
Day_24_DNA
Day_31_No_substrate
Day_31_DNA

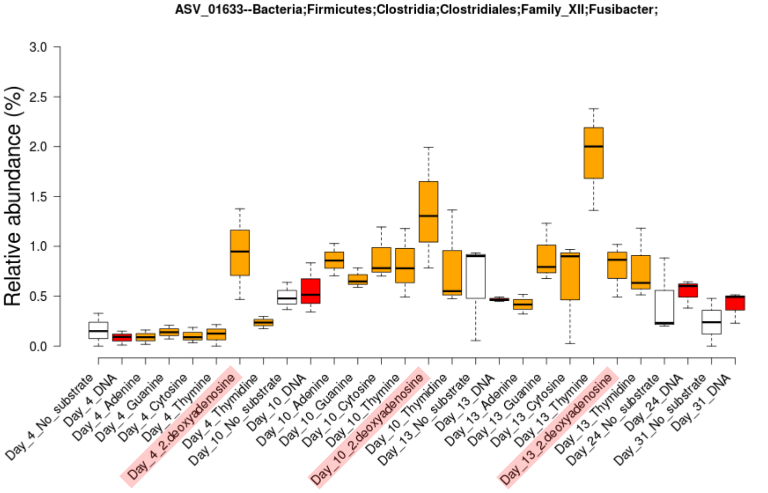
ASV_01633--Bacteria;Firmicutes;Clostridia;Clostridiales;Family_XII;Fusibacter;
Relative abundance (%)
0.0
0.5
1.0
1.5
2.0
2.5
3.0
Day_4_No_substrate
Day_4_DNA
Day_4_Adenine
Day_4_Guanine
Day_4_Cytosine
Day_4_Thymine
Day_4_2deoxyadenosine
Day_4_Thymidine
Day_10_No_substrate
Day_10_DNA
Day_10_Adenine
Day_10_Guanine
Day_10_Cytosine
Day_10_Thymine
Day_10_2deoxyadenosine
Day_10_Thymidine
Day_13_No_substrate
Day_13_DNA
Day_13_Adenine
Day_13_Guanine
Day_13_Cytosine
Day_13_Thymine
Day_13_2deoxyadenosine
Day_13_Thymidine
Day_24_No_substrate
Day_24_DNA
Day_31_No_substrate
Day_31_DNA

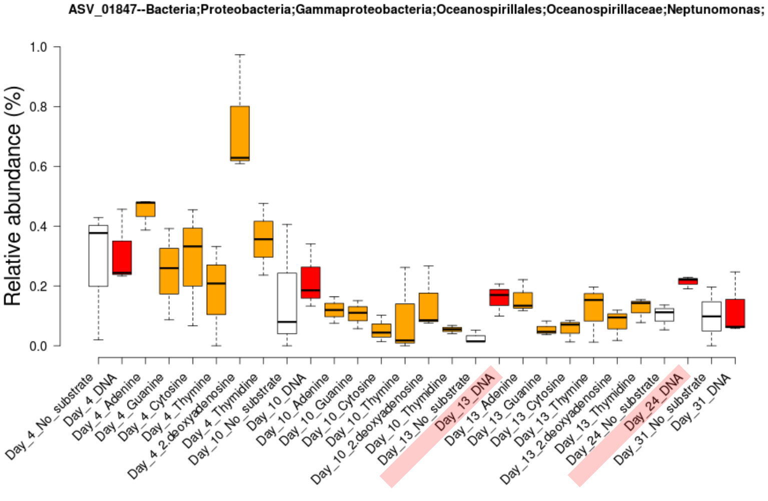
ASV_01847--Bacteria;Proteobacteria;Gammaproteobacteria;Oceanospirillales;Oceanospirillaceae;Neptunomonas;
Relative abundance (%)
0.0
0.2
0.4
0.6
0.8
1.0
Day_4_No_substrate
Day_4_DNA
Day_4_Adenine
Day_4_Guanine
Day_4_Cytosine
Day_4_Thymine
Day_4_2deoxyadenosine
Day_4_Thymidine
Day_10_No_substrate
Day_10_DNA
Day_10_Adenine
Day_10_Guanine
Day_10_Cytosine
Day_10_Thymine
Day_10_2deoxyadenosine
Day_10_Thymidine
Day_13_No_substrate
Day_13_DNA
Day_13_Adenine
Day_13_Guanine
Day_13_Cytosine
Day_13_Thymine
Day_13_2deoxyadenosine
Day_13_Thymidine
Day_24_No_substrate
Day_24_DNA
Day_31_No_substrate
Day_31_DNA

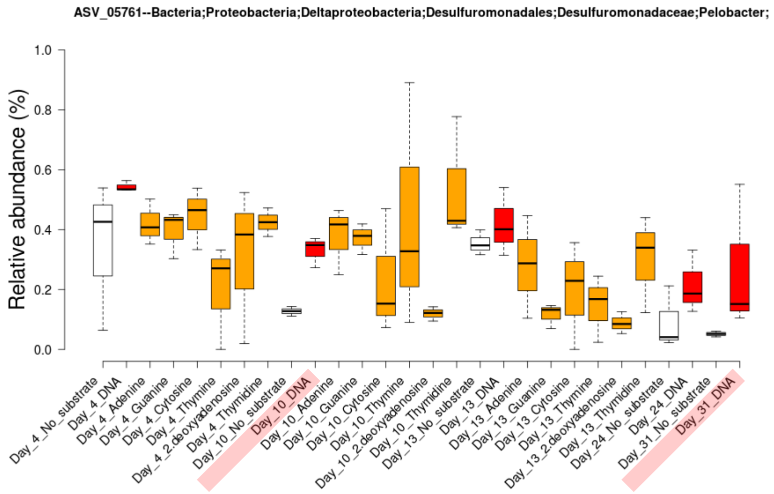
ASV_05761--Bacteria;Proteobacteria;Deltaproteobacteria;Desulfuromonadales;Desulfuromonadaceae;Pelobacter;
Relative abundance (%)
0.0
0.2
0.4
0.6
0.8
1.0
Day_4_No_substrate
Day_4_DNA
Day_4_Adenine
Day_4_Guanine
Day_4_Cytosine
Day_4_Thymine
Day_4_2deoxyadenosine
Day_4_Thymidine
Day_10_No_substrate
Day_10_DNA
Day_10_Adenine
Day_10_Guanine
Day_10_Cytosine
Day_10_Thymine
Day_10_2deoxyadenosine
Day_10_Thymidine
Day_13_No_substrate
Day_13_DNA
Day_13_Adenine
Day_13_Guanine
Day_13_Cytosine
Day_13_Thymine
Day_13_2deoxyadenosine
Day_13_Thymidine
Day_24_No_substrate
Day_24_DNA
Day_31_No_substrate
Day_31_DNA

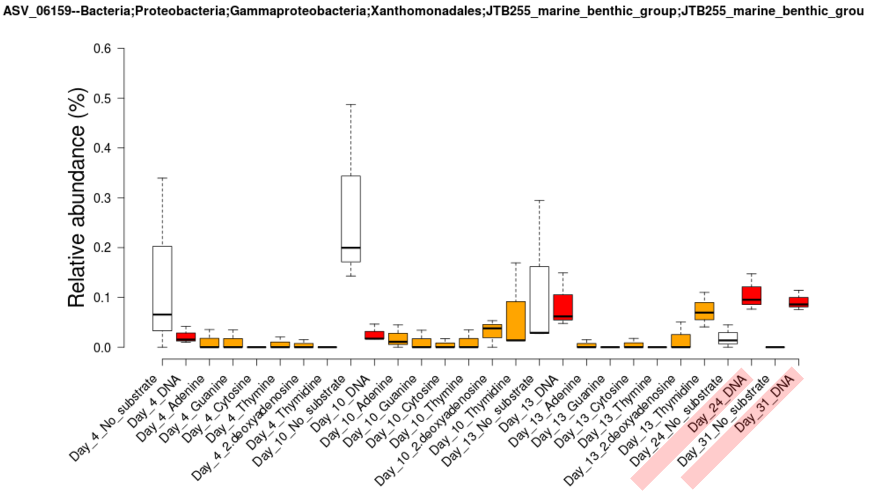
ASV_06159--Bacteria;Proteobacteria;Gammaproteobacteria;Xanthomonadales;JTB255_marine_benthic_group;JTB255_marine_benthic_grou
Relative abundance (%)
0.0
0.1
0.2
0.3
0.4
0.5
0.6
Day_4_No_substrate
Day_4_DNA
Day_4_Adenine
Day_4_Guanine
Day_4_Cytosine
Day_4_Thymine
Day_4_2deoxyadenosine
Day_4_Thymidine
Day_10_No_substrate
Day_10_DNA
Day_10_Adenine
Day_10_Guanine
Day_10_Cytosine
Day_10_Thymine
Day_10_2deoxyadenosine
Day_10_Thymidine
Day_13_No_substrate
Day_13_DNA
Day_13_Adenine
Day_13_Guanine
Day_13_Cytosine
Day_13_Thymine
Day_13_2deoxyadenosine
Day_13_Thymidine
Day_24_No_substrate
Day_24_DNA
Day_31_No_substrate
Day_31_DNA

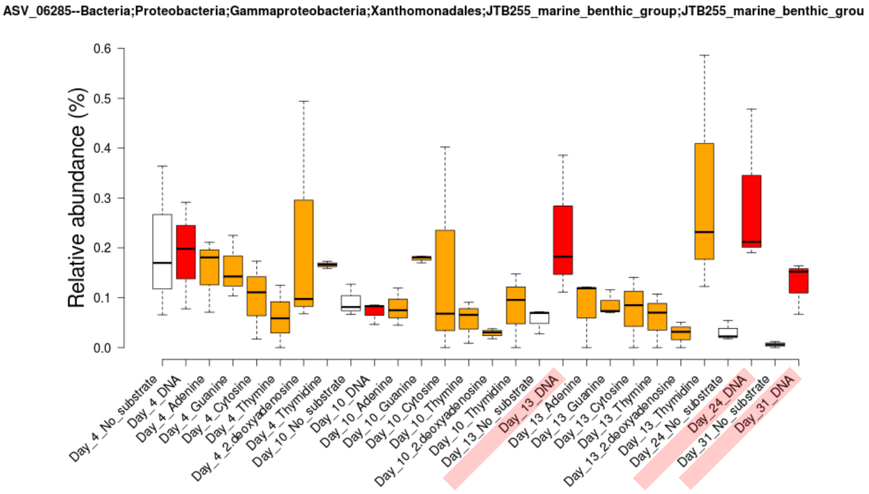
ASV_06285--Bacteria;Proteobacteria;Gammaproteobacteria;Xanthomonadales;JTB255_marine_benthic_group;JTB255_marine_benthic_grou
Relative abundance (%)
0.0
0.1
0.2
0.3
0.4
0.5
0.6
Day_4_No_substrate
Day_4_DNA
Day_4_Adenine
Day_4_Guanine
Day_4_Cytosine
Day_4_Thymine
Day_4_2deoxyadenosine
Day_4_Thymidine
Day_10_No_substrate
Day_10_DNA
Day_10_Adenine
Day_10_Guanine
Day_10_Cytosine
Day_10_Thymine
Day_10_2deoxyadenosine
Day_10_Thymidine
Day_13_No_substrate
Day_13_DNA
Day_13_Adenine
Day_13_Guanine
Day_13_Cytosine
Day_13_Thymine
Day_13_2deoxyadenosine
Day_13_Thymidine
Day_24_No_substrate
Day_24_DNA
Day_31_No_substrate
Day_31_DNA

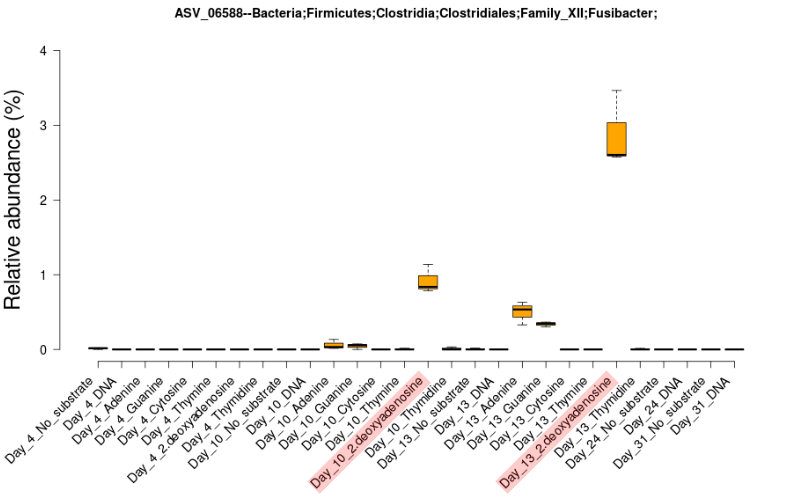
ASV_06588--Bacteria;Firmicutes;Clostridia;Clostridiales;Family_XII;Fusibacter;
Relative abundance (%)
0
1
2
3
4
Day_4_No_substrate
Day_4_DNA
Day_4_Adenine
Day_4_Guanine
Day_4_Cytosine
Day_4_Thymine
Day_4_2deoxyadenosine
Day_4_Thymidine
Day_10_No_substrate
Day_10_DNA
Day_10_Adenine
Day_10_Guanine
Day_10_Cytosine
Day_10_Thymine
Day_10_2deoxyadenosine
Day_10_Thymidine
Day_13_No_substrate
Day_13_DNA
Day_13_Adenine
Day_13_Guanine
Day_13_Cytosine
Day_13_Thymine
Day_13_2deoxyadenosine
Day_13_Thymidine
Day_24_No_substrate
Day_24_DNA
Day_31_No_substrate
Day_31_DNA

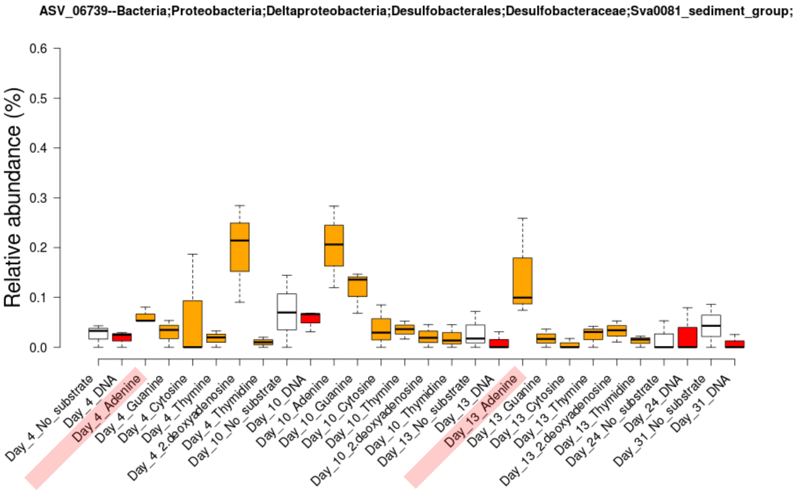
ASV_06739--Bacteria;Proteobacteria;Deltaproteobacteria;Desulfobacterales;Desulfobacteraceae;Sva0081_sediment_group;
Relative abundance (%)
0.0
0.1
0.2
0.3
0.4
0.5
0.6
Day_4_No_substrate
Day_4_DNA
Day_4_Adenine
Day_4_Guanine
Day_4_Cytosine
Day_4_Thymine
Day_4_2deoxyadenosine
Day_4_Thymidine
Day_10_No_substrate
Day_10_DNA
Day_10_Adenine
Day_10_Guanine
Day_10_Cytosine
Day_10_Thymine
Day_10_2deoxyadenosine
Day_10_Thymidine
Day_13_No_substrate
Day_13_DNA
Day_13_Adenine
Day_13_Guanine
Day_13_Cytosine
Day_13_Thymine
Day_13_2deoxyadenosine
Day_13_Thymidine
Day_24_No_substrate
Day_24_DNA
Day_31_No_substrate
Day_31_DNA

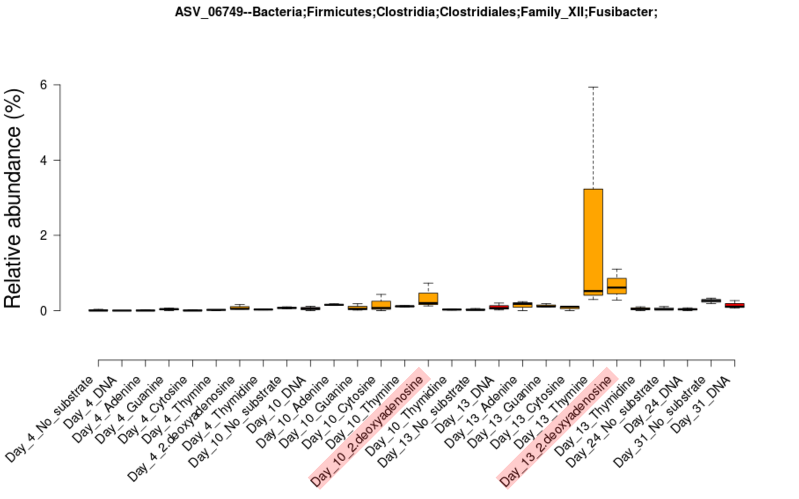
ASV_06749--Bacteria;Firmicutes;Clostridia;Clostridiales;Family_XII;Fusibacter;
Relative abundance (%)
0
2
4
6
Day_4_No_substrate
Day_4_DNA
Day_4_Adenine
Day_4_Guanine
Day_4_Cytosine
Day_4_Thymine
Day_4_2deoxyadenosine
Day_4_Thymidine
Day_10_No_substrate
Day_10_DNA
Day_10_Adenine
Day_10_Guanine
Day_10_Cytosine
Day_10_Thymine
Day_10_2deoxyadenosine
Day_10_Thymidine
Day_13_No_substrate
Day_13_DNA
Day_13_Adenine
Day_13_Guanine
Day_13_Cytosine
Day_13_Thymine
Day_13_2deoxyadenosine
Day_13_Thymidine
Day_24_No_substrate
Day_24_DNA
Day_31_No_substrate
Day_31_DNA

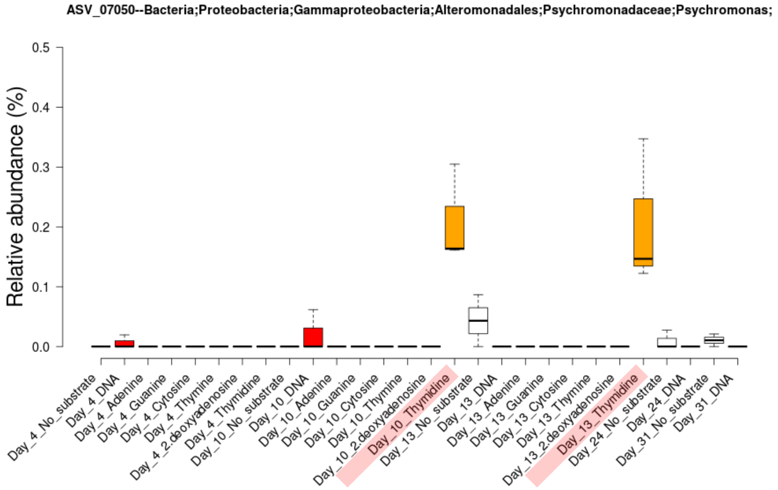
ASV_07050--Bacteria;Proteobacteria;Gammaproteobacteria;Alteromonadales;Psychromonadaceae;Psychromonas;
Relative abundance (%)
0.5
0.4
0.3
0.2
0.1
0.0
Day_4_No_substrate
Day_4_DNA
Day_4_Adenine
Day_4_Guanine
Day_4_Cytosine
Day_4_Thymine
Day_4_2deoxyadenosine
Day_4_Thymidine
Day_10_No_substrate
Day_10_DNA
Day_10_Adenine
Day_10_Guanine
Day_10_Cytosine
Day_10_Thymine
Day_10_2deoxyadenosine
Day_10_Thymidine
Day_13_No_substrate
Day_13_DNA
Day_13_Adenine
Day_13_Guanine
Day_13_Cytosine
Day_13_Thymine
Day_13_2deoxyadenosine
Day_13_Thymidine
Day_24_No_substrate
Day_24_DNA
Day_31_No_substrate
Day_31_DNA

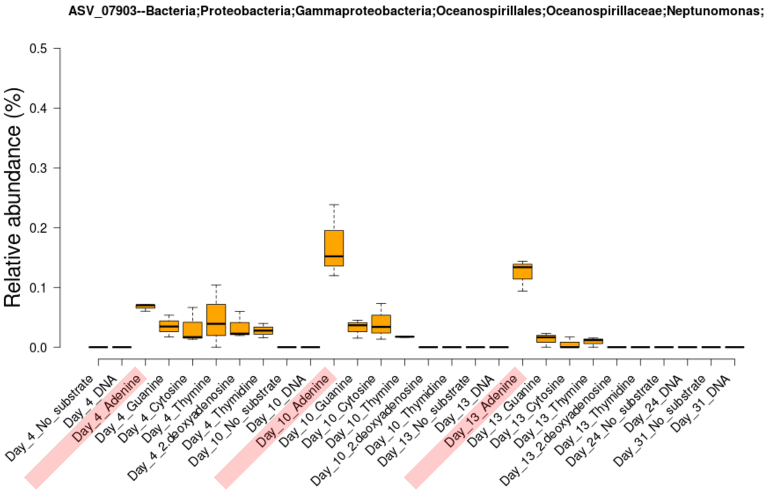
ASV_07903--Bacteria;Proteobacteria;Gammaproteobacteria;Oceanospirillales;Oceanospirillaceae;Neptunomonas;
Relative abundance (%)
0.5
0.4
0.3
0.2
0.1
0.0
Day_4_No_substrate
Day_4_DNA
Day_4_Adenine
Day_4_Guanine
Day_4_Cytosine
Day_4_Thymine
Day_4_2deoxyadenosine
Day_4_Thymidine
Day_10_No_substrate
Day_10_DNA
Day_10_Adenine
Day_10_Guanine
Day_10_Cytosine
Day_10_Thymine
Day_10_2deoxyadenosine
Day_10_Thymidine
Day_13_No_substrate
Day_13_DNA
Day_13_Adenine
Day_13_Guanine
Day_13_Cytosine
Day_13_Thymine
Day_13_2deoxyadenosine
Day_13_Thymidine
Day_24_No_substrate
Day_24_DNA
Day_31_No_substrate
Day_31_DNA

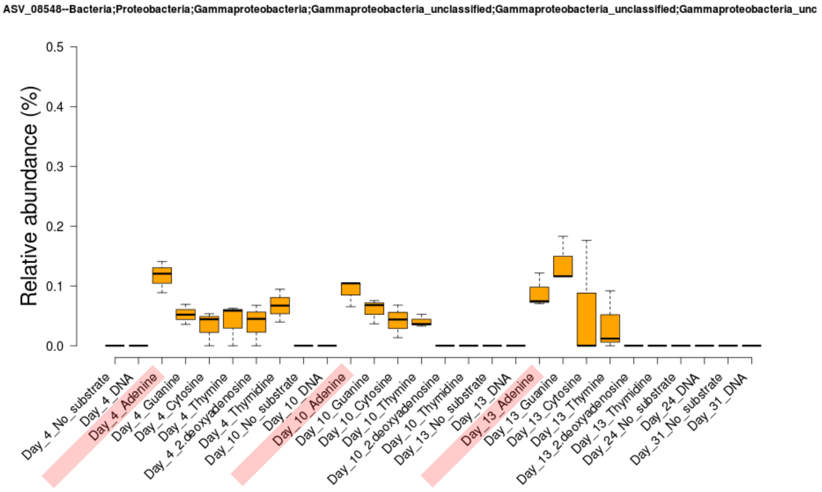
ASV_08548--Bacteria;Proteobacteria;Gammaproteobacteria;Gammaproteobacteria_unclassified;Gammaproteobacteria_unclassified;Gammaproteobacteria_unc
Relative abundance (%)
0.5
0.4
0.3
0.2
0.1
0.0
Day_4_No_substrate
Day_4_DNA
Day_4_Adenine
Day_4_Guanine
Day_4_Cytosine
Day_4_Thymine
Day_4_2deoxyadenosine
Day_4_Thymidine
Day_10_No_substrate
Day_10_DNA
Day_10_Adenine
Day_10_Guanine
Day_10_Cytosine
Day_10_Thymine
Day_10_2deoxyadenosine
Day_10_Thymidine
Day_13_No_substrate
Day_13_DNA
Day_13_Adenine
Day_13_Guanine
Day_13_Cytosine
Day_13_Thymine
Day_13_2deoxyadenosine
Day_13_Thymidine
Day_24_No_substrate
Day_24_DNA
Day_31_No_substrate
Day_31_DNA

ASV_09061--Bacteria;Firmicutes;Clostridia;Clostridiales;Family_XII;Fusibacter;

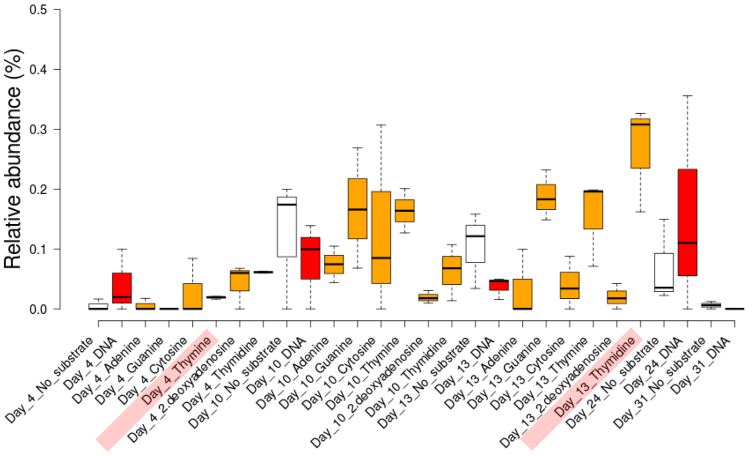

ASV_10750--Bacteria;Proteobacteria;Gammaproteobacteria;Alteromonadales;Psychromonadaceae;Psychromonas;

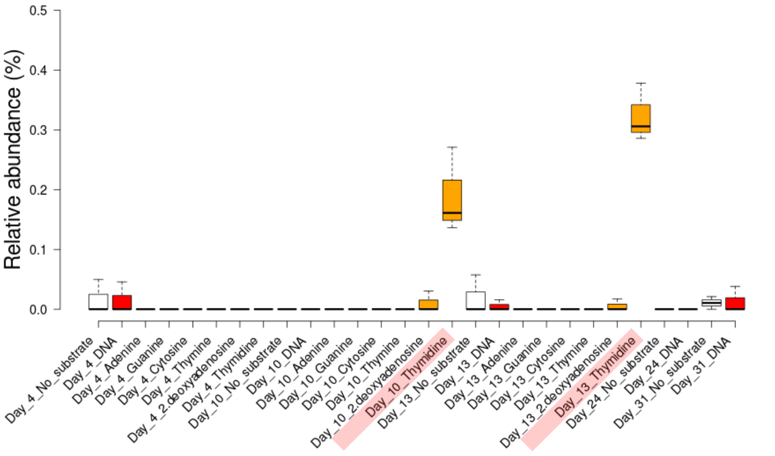

ASV_11797--Bacteria;Firmicutes;Clostridia;Clostridiales;Family_XII;Fusibacter;

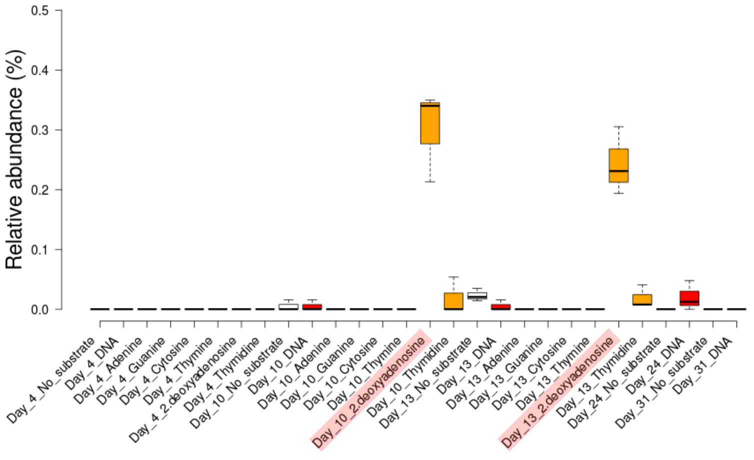

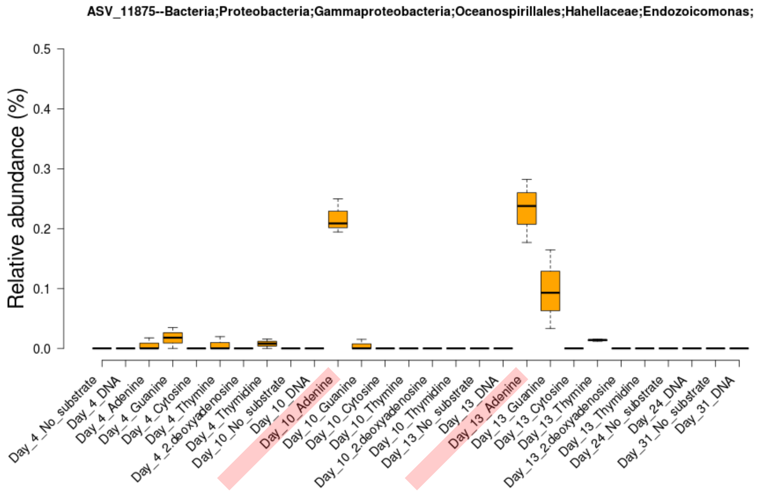
ASV_11875--Bacteria;Proteobacteria;Gammaproteobacteria;Oceanospirillales;Hahellaceae;Endozoicomonas;
Relative abundance (%)
0.0
0.1
0.2
0.3
0.4
0.5
Day_4_No_substrate
Day_4_DNA
Day_4_Adenine
Day_4_Guanine
Day_4_Cytosine
Day_4_Thymine
Day_4_2.deoxyadenosine
Day_4_Thymidine
Day_10_No_substrate
Day_10_DNA
Day_10_Adenine
Day_10_Guanine
Day_10_Cytosine
Day_10_Thymine
Day_10_2.deoxyadenosine
Day_10_Thymidine
Day_13_No_substrate
Day_13_DNA
Day_13_Adenine
Day_13_Guanine
Day_13_Cytosine
Day_13_Thymine
Day_13_2.deoxyadenosine
Day_13_Thymidine
Day_24_No_substrate
Day_24_DNA
Day_31_No_substrate
Day_31_DNA

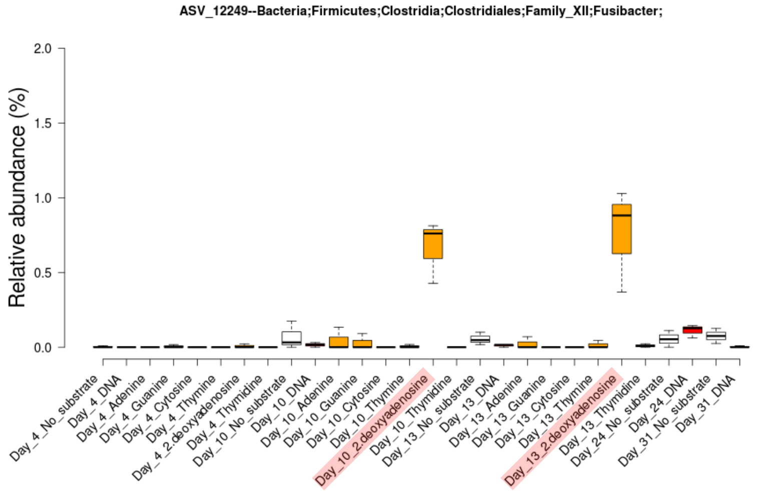
ASV_12249--Bacteria;Firmicutes;Clostridia;Clostridiales;Family_XII;Fusibacter;
Relative abundance (%)
0.0
0.5
1.0
1.5
2.0
Day_4_No_substrate
Day_4_DNA
Day_4_Adenine
Day_4_Guanine
Day_4_Cytosine
Day_4_Thymine
Day_4_2.deoxyadenosine
Day_4_Thymidine
Day_10_No_substrate
Day_10_DNA
Day_10_Adenine
Day_10_Guanine
Day_10_Cytosine
Day_10_Thymine
Day_10_2.deoxyadenosine
Day_10_Thymidine
Day_13_No_substrate
Day_13_DNA
Day_13_Adenine
Day_13_Guanine
Day_13_Cytosine
Day_13_Thymine
Day_13_2.deoxyadenosine
Day_13_Thymidine
Day_24_No_substrate
Day_24_DNA
Day_31_No_substrate
Day_31_DNA

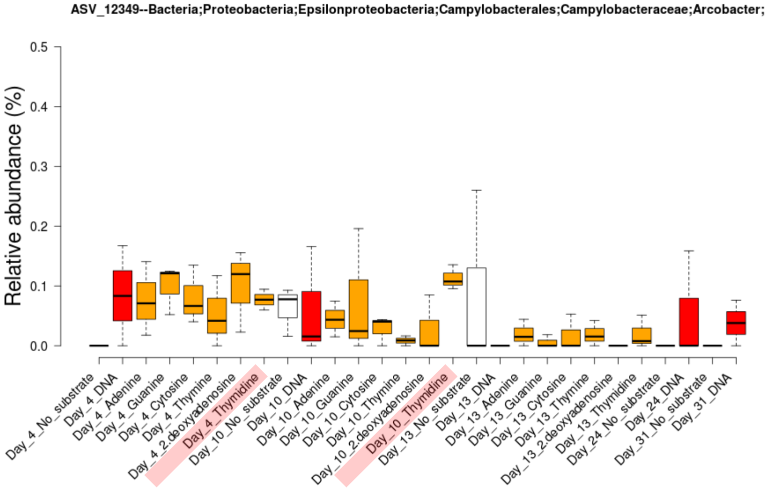
ASV_12349--Bacteria;Proteobacteria;Epsilonproteobacteria;Campylobacterales;Campylobacteraceae;Arcobacter;
Relative abundance (%)
0.0
0.1
0.2
0.3
0.4
0.5
Day_4_No_substrate
Day_4_DNA
Day_4_Adenine
Day_4_Guanine
Day_4_Cytosine
Day_4_Thymine
Day_4_2.deoxyadenosine
Day_4_Thymidine
Day_10_No_substrate
Day_10_DNA
Day_10_Adenine
Day_10_Guanine
Day_10_Cytosine
Day_10_Thymine
Day_10_2.deoxyadenosine
Day_10_Thymidine
Day_13_No_substrate
Day_13_DNA
Day_13_Adenine
Day_13_Guanine
Day_13_Cytosine
Day_13_Thymine
Day_13_2.deoxyadenosine
Day_13_Thymidine
Day_24_No_substrate
Day_24_DNA
Day_31_No_substrate
Day_31_DNA

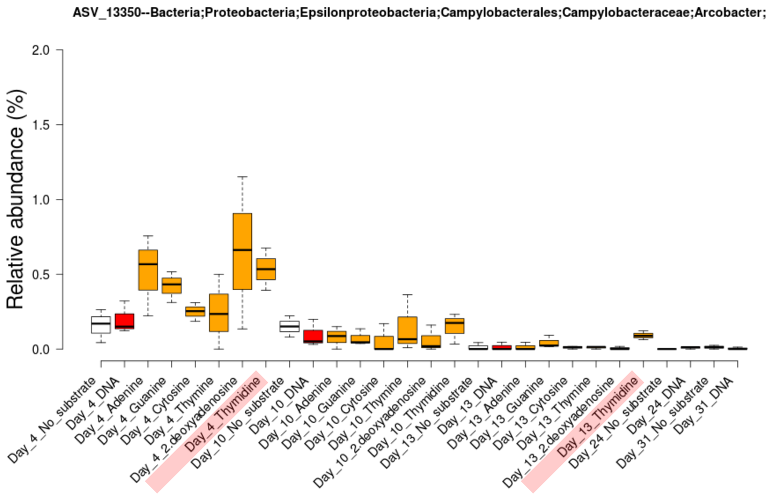
ASV_13350--Bacteria;Proteobacteria;Epsilonproteobacteria;Campylobacterales;Campylobacteraceae;Arcobacter;
Relative abundance (%)
0.0
0.5
1.0
1.5
2.0
Day_4_No_substrate
Day_4_DNA
Day_4_Adenine
Day_4_Guanine
Day_4_Cytosine
Day_4_Thymine
Day_4_2.deoxyadenosine
Day_4_Thymidine
Day_10_No_substrate
Day_10_DNA
Day_10_Adenine
Day_10_Guanine
Day_10_Cytosine
Day_10_Thymine
Day_10_2.deoxyadenosine
Day_10_Thymidine
Day_13_No_substrate
Day_13_DNA
Day_13_Adenine
Day_13_Guanine
Day_13_Cytosine
Day_13_Thymine
Day_13_2.deoxyadenosine
Day_13_Thymidine
Day_24_No_substrate
Day_24_DNA
Day_31_No_substrate
Day_31_DNA

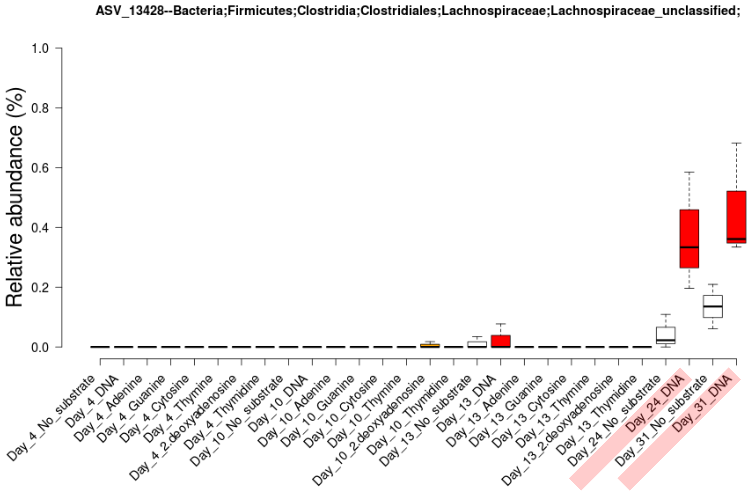
ASV_13428--Bacteria;Firmicutes;Clostridia;Clostridiales;Lachnospiraceae;Lachnospiraceae_unclassified;
Relative abundance (%)
0.0
0.2
0.4
0.6
0.8
1.0
Day_4_No_substrate
Day_4_DNA
Day_4_Adenine
Day_4_Guanine
Day_4_Cytosine
Day_4_Thymine
Day_4_2.deoxyadenosine
Day_4_Thymidine
Day_10_No_substrate
Day_10_DNA
Day_10_Adenine
Day_10_Guanine
Day_10_Cytosine
Day_10_Thymine
Day_10_2.deoxyadenosine
Day_10_Thymidine
Day_13_No_substrate
Day_13_DNA
Day_13_Adenine
Day_13_Guanine
Day_13_Cytosine
Day_13_Thymine
Day_13_2.deoxyadenosine
Day_13_Thymidine
Day_24_No_substrate
Day_24_DNA
Day_31_No_substrate
Day_31_DNA

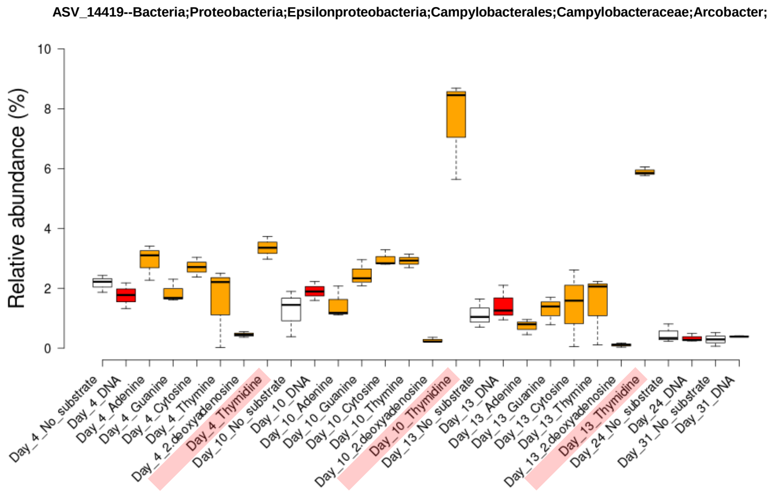
ASV_14419--Bacteria;Proteobacteria;Epsilonproteobacteria;Campylobacterales;Campylobacteraceae;Arcobacter;
Relative abundance (%)
0
2
4
6
8
10
Day_4_No_substrate
Day_4_DNA
Day_4_Adenine
Day_4_Guanine
Day_4_Cytosine
Day_4_Thymine
Day_4_2.deoxyadenosine
Day_4_Thymidine
Day_10_No_substrate
Day_10_DNA
Day_10_Adenine
Day_10_Guanine
Day_10_Cytosine
Day_10_Thymine
Day_10_2.deoxyadenosine
Day_10_Thymidine
Day_13_No_substrate
Day_13_DNA
Day_13_Adenine
Day_13_Guanine
Day_13_Cytosine
Day_13_Thymine
Day_13_2.deoxyadenosine
Day_13_Thymidine
Day_24_No_substrate
Day_24_DNA
Day_31_No_substrate
Day_31_DNA

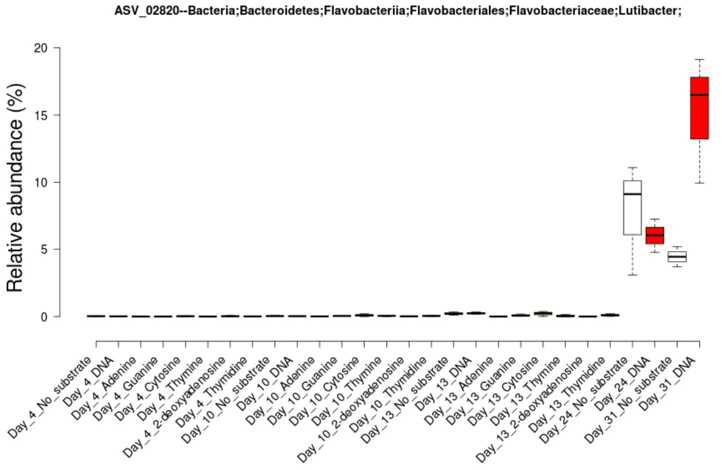

ASV_02820--Bacteria;Bacteroidetes;Flavobacteriia;Flavobacteriales;Flavobacteriaceae;Lutibacter;
Relative abundance (%)
0
5
10
15
20
Day_4_No_substrate
Day_4_DNA
Day_4_Adenine
Day_4_Guanine
Day_4_Cytosine
Day_4_Thymine
Day_4_2-deoxyadenosine
Day_4_Thymidine
Day_10_No_substrate
Day_10_DNA
Day_10_Adenine
Day_10_Guanine
Day_10_Cytosine
Day_10_Thymine
Day_10_2-deoxyadenosine
Day_10_Thymidine
Day_13_No_substrate
Day_13_DNA
Day_13_Adenine
Day_13_Guanine
Day_13_Cytosine
Day_13_Thymine
Day_13_2-deoxyadenosine
Day_13_Thymidine
Day_24_No_substrate
Day_24_DNA
Day_31_No_substrate
Day_31_DNA
