# Supplementary material for: Genomic insights into diverse bacterial taxa that degrade extracellular DNA in marine sediments
Source: Nat Microbiol. 2021 Jun 14;6(7):885–98. doi: 10.1038/s41564-021-00917-9 (PMC8289736; doi:10.1038/s41564-021-00917-9)
Supplement: Supplementary file 2 — Reporting Summary [file 41564_2021_917_MOESM2_ESM.pdf]

nature research

Corresponding author(s): Kenneth Wasmund

Last updated by author(s): Apr 28, 2021

Reporting Summary

Nature Research wishes to improve the reproducibility of the work that we publish. This form provides structure for consistency and transparency in reporting. For further information on Nature Research policies, see Authors & Referees and the Editorial Policy Checklist.

Statistics

For all statistical analyses, confirm that the following items are present in the figure legend, table legend, main text, or Methods section.

n/a	Confirmed	
☐	☒	The exact sample size (*n*) for each experimental group/condition, given as a discrete number and unit of measurement
☐	☒	A statement on whether measurements were taken from distinct samples or whether the same sample was measured repeatedly
☐	☒	The statistical test(s) used AND whether they are one- or two-sided<br>*Only common tests should be described solely by name; describe more complex techniques in the Methods section.*
☒	☐	A description of all covariates tested
☒	☐	A description of any assumptions or corrections, such as tests of normality and adjustment for multiple comparisons
☐	☒	A full description of the statistical parameters including central tendency (e.g. means) or other basic estimates (e.g. regression coefficient) AND variation (e.g. standard deviation) or associated estimates of uncertainty (e.g. confidence intervals)
☐	☒	For null hypothesis testing, the test statistic (e.g. *F*, *t*, *r*) with confidence intervals, effect sizes, degrees of freedom and *P* value noted<br>*Give P values as exact values whenever suitable.*
☒	☐	For Bayesian analysis, information on the choice of priors and Markov chain Monte Carlo settings
☒	☐	For hierarchical and complex designs, identification of the appropriate level for tests and full reporting of outcomes
☒	☐	Estimates of effect sizes (e.g. Cohen's *d*, Pearson's *r*), indicating how they were calculated

Our web collection on statistics for biologists contains articles on many of the points above.

Software and code

Policy information about availability of computer code

Data collection	Detailed in the Materials and Methods or Supplementary Information:<br><br>FACS -- BD FACSChorus v1.3.<br>NanoSIMS -- NS50L, version 4.3<br>CFX96 Touch™ Real-Time PCR Detection System (version 3.1) (Bio-Rad)
Data analysis	All code used was part of software packages detailed in the Materials and Methods or Supplementary Information:<br><br>Code for metagenomic sequence read trimming is available here (https://github.com/kwasmund/Trim-Illumina.git)<br><br>mothur (version 1.33.0)<br>IDBA-UD (version, 1.1.1)<br>MetaBat2 (version 2.12.1)<br>MaxBin2 (version 2.2.4)<br>CONCOCT (version 0.4.1)

samtools (version 1.9)
MetaSpades (version 3.11.1)
DasTool (version 1.1.0)
dRep (version 1.4.3)
CheckM (version 1.0.7)
SignalP (v5.0)
DADA2. (version 1.16)
RAST server (annotations used 'classic RAST' pipeline) (https://rast.nmpdr.org/)
BLASTP (web-server) (https://blast.ncbi.nlm.nih.gov/Blast.cgi?PROGRAM=blastp&BLAST_PROGRAMS=blastp&PAGE_TYPE=BlastSearch&SHOW_DEFAULTS=on&LINK_LOC=blasthome)
Rhea package (version 1.0.1-5)
R software environment (version 1.1.383)
'vegan' package (version 2.5-3) (in R)
PSORTb (version 3.0)
ARB (version 6.0.6)
RaxML (in ARB) (version 7.7.2)
PhyML (in ARB) (version 2.4.5)
fastDNAml (in ARB) (version 1.1.0)
Genome Taxonomy Database (GTDB) (version 0.1.3)
JSpeciesWS webserver (http://jspecies.ribohost.com/jspeciesws/#analyse)

WinImage software package (version 2.0.8) (from Cameca)
BWA (version 0.7.16a)

For manuscripts utilizing custom algorithms or software that are central to the research but not yet described in published literature, software must be made available to editors/reviewers. We strongly encourage code deposition in a community repository (e.g. GitHub). See the Nature Research guidelines for submitting code & software for further information.

Data

Policy information about availability of data

All manuscripts must include a data availability statement. This statement should provide the following information, where applicable:

- Accession codes, unique identifiers, or web links for publicly available datasets
- A list of figures that have associated raw data
- A description of any restrictions on data availability

All sequence data is available via the NCBI-Genbank repository and accession numbers are detailed in the manuscript.
"All sequence data was deposited under Genbank Bioproject PRJNA510104. PCR-derived 16S rRNA gene amplicon sequence data performed by Microsynth (microcosms) is available under accessions SAMN10603326-SAMN10603488. PCR-derived 16S rRNA gene amplicon sequence data performed by the JMF (DNA-SIP gradients) is available under accessions SAMN13338678-SAMN13338783. Metagenomic sequence read data from Greenland microcoms is available under accessions SAMN10594394-SAMN10594398. Metagenome-assembled genomes from Greenland microcosms are available under accessions SAMN10805732-SAMN10805736 and SAMN12272019-SAMN12272029. Metagenomic sequence read data from Svalbard marine sediments are available under Bioproject accessions PRJNA493859-PRJNA623111. Metagenome-assembled genomes from Svalbard sediments are available under Bioproject PRJNA623111 and accessions JADWMF000000000-JADWMK000000000. The 16S rRNA gene amplicon sequence data for the in-situ communities is available under Bioproject accession PRJNA682441 and SRA accessions SAMN16990562- SAMN16990567."

Previously generated metagenomic datasets and metagenome-assembled genomes that were reanalysed in this study are available under NCBI-Genbank Bioprojects: PRJNA270657 (Baker et al. 2015, White Oak Estuary, USA); PRJNA515295 (Kessler et al. 2012, Sandy sediments, Australia; and PRJNA362212 (Dombrowski et al. 2018, Guaymas Basin, USA).

Databases used were: Genome Taxonomy Database (GTDB) version 0.1.3 (https://gtdb.ecogenomic.org/); IMNGS webserver (as of Nov. 2018) (https://www.imngs.org/); Integrated Microbial Genomes and Microbiomes (IMG/M) server (https://img.jgi.doe.gov/); SILVA ProbeMatch server (https://www.arb-silva.de/search/testprobe/); Conserved Domain Database (CDD) search server (https://www.ncbi.nlm.nih.gov/Structure/cdd/cdd.shtml); SILVA 119 SSU NR99 database (https://www.arb-silva.de/download/arb-files/); Short Read Archive (https://www.ncbi.nlm.nih.gov/sra); NCBI-nr (https://www.ncbi.nlm.nih.gov/protein/); MetaCyc database (https://metacyc.org/).

Field-specific reporting

Please select the one below that is the best fit for your research. If you are not sure, read the appropriate sections before making your selection.

☒ Life sciences ☐ Behavioural & social sciences ☐ Ecological, evolutionary & environmental sciences

For a reference copy of the document with all sections, see nature.com/documents/nr-reporting-summary-flat.pdf

Life sciences study design

All studies must disclose on these points even when the disclosure is negative.

Sample size	No statistical methods were used to pre-determine sample size. In all cases, we performed triplicates/sampling of all experimental treatments, and from three-to-five time points (for DNA-SIP) and two or more time-points (for microcosm comparisons). Triplicates were taken to provide enough samples for a minimum number needed for statistical comparisons and are standard practise in such microbial

	ecology experiments. We also had to take into account the feasibility of having limited amounts of starting sediment material for the various treatments. When combined with the time-series results and only classifiying taxa as significantly enriched among same treatments if detected at multiple time-points, this should provide sufficient distinguishing power. The time-series was subsampled over one month was performed to capture activity among relatively slow growing sediment bacteria. The amounts of sediments added to each microcosm was selected based on providing sufficient material to sub-sample small fractions over time.
Data exclusions	No data was excluded.
Replication	The overall microcosm experiment was performed once. In all cases, we performed triplicates/sampling of all experimental treatments. We deemed this successful because triplicates showed reproducible taxa distributions, and slow and reproducible shifts in relative abundances over time. For species-level relative abundance comparisons of 16S rRNA gene sequence data, we only reported treatment-induced differences if they were statistically significant at two or more time points (and specifically state if otherwise). The NanoSIMS was also performed once, and was deemed successful because the isotopic labelling was very highly significant compared to control cells, and was supported by the DNA-SIP results.
Randomization	Sediments were thoroughly mixed for over 5 mins prior to allocation to microcosms, and the allocations to microcosms was performed randomly.
Blinding	Blinding was not relevant to this study because the subject matter is 'inert'.

Reporting for specific materials, systems and methods

We require information from authors about some types of materials, experimental systems and methods used in many studies. Here, indicate whether each material, system or method listed is relevant to your study. If you are not sure if a list item applies to your research, read the appropriate section before selecting a response.

Materials & experimental systems

n/a	Involved in the study
☒	☐ Antibodies
☒	☐ Eukaryotic cell lines
☒	☐ Palaeontology
☒	☐ Animals and other organisms
☒	☐ Human research participants
☒	☐ Clinical data

Methods

n/a	Involved in the study
☒	☐ ChIP-seq
☐	☒ Flow cytometry
☒	☐ MRI-based neuroimaging

Flow Cytometry

Plots

Confirm that:

☒ The axis labels state the marker and fluorochrome used (e.g. CD4-FITC).

☒ The axis scales are clearly visible. Include numbers along axes only for bottom left plot of group (a 'group' is an analysis of identical markers).

☐ All plots are contour plots with outliers or pseudocolor plots.

☐ A numerical value for number of cells or percentage (with statistics) is provided.

Methodology

Sample preparation	Cells of microorganisms from formaldehyde-fixed microcosm sediment samples were extracted from previously fixed sediments which were stored in PBS:ethanol (1:1) at -20 ℃. Ethanol was removed from PBS:ethanol samples (500 µl) by pelleting and washing 2 times with PBS. Samples were diluted in 1.8 ml PBS, and then sodium pyrophosphate (0.1% final) and tween 20 (0.5% final) were added. They were vortexed for 20 mins at medium speed (4-5) with a Vortex Genie 2 vortexer (Scientific Industries), with tubes closed with parafilm and taped-down horizontally. Samples were sonicated with 50% power for 20 s with setting ‘5’, on ice (UW 2070 needle, Bandelin Electronics). Cell suspensions were made-up to 4 ml with PBS in 13.2 ml Thinwall Polypropylene Tubes (Beckman Coulter), and then 2 ml of Nycodenz solution (80% w/v) (Alere Technologies. Cat. no. 1002424) was injected carefully under the cell suspension by a long needle and syringe. Samples were centrifuged for 90 mins at 4 ℃ in a SW 41 Ti Swinging-Bucket Rotor (Beckman Coulter) at 14000 g, with no deceleration when stopping. Total supernatant was collected to a new tube, and then ethanol was added to produce a 1:1 final solution. The collected solutions were stored at -20 ℃.<br>For CARD-FISH standard protocols and buffers were used 58. Samples of ~ 500 µl were filtered onto polycarbonate filters (GTTP type, 0.2 µm pore size) (Millipore. Cat. no. GTTP02500), and then PBS (5 ml) was washed through. Filters were dried at 46℃ for 5 mins before CARD-FISH. The newly designed 5’-horseradish peroxidase-labeled (HRP) probe Fusi-6-HRP (5’-TTCCTTAGGTACCGTCATTTTTCT-3’) (Biomers) and the unlabelled helper probe Fusi-6-HelpR (5’-GGCACGTATTTAGCCGGTGC-3’) were used for hybridisations targeting populations representing the most abundant Fusibacteraceae ASV. The NONEUB probe (5’-ACTCCTACGGGAGGCAGC-3’) was used for negative controls and to gauge background fluorescence during FACS. The Fusi-6-

HRP probe was designed to specifically target the most abundant Fusibacteraceae ASV (ASV_09916) and relatives in our 16S rRNA gene amplicon sequence dataset. The probe also matched other (n=25) related Fusibacteraceae ASVs (all >91% sequence identity to ASV_09916), although those sequences were generally in low abundance (<0.5% on average across microcosms and time-points). Although few other ASVs from other taxa also matched (n=20), they all represented ASVs with extremely low abundances, i.e., the most abundant of these ASVs had a maximum relative abundance of 0.0003%. When checked using the ProbeMatch function in SILVA94, only 29 hits were obtained for Fusibacteraceae sequences, and only 1 off-target match came from the Cyanobacteria. We therefore deemed that the Fusi-6 probe should primarily detect the abundant Fusibacteraceae populations.
Hybridisation of probes were performed overnight (~14-16 hrs) at 35°C with 50% formamide, using previously described protocols and using lysozyme (Sigma-Aldrich. Cat. no. 62970) permeabilisation58, and Oregon-Green 488-labeled tyramides (Thermo Fischer. Cat. no. T20919). The overnight hybridisation time enabled penetration of probes into the cells. The hybridisations were performed with whole filters, where the filters were carefully added to 2 ml tubes with 300 µl of CARD-FISH hybridisation buffer with probes, mixed, and placed horizontally during hybridisation so the buffer covered most of the filters. After probe washing, and after tyramide signal amplification and washing58, cells were scrapped-off filters by adding filters to the lids of 50 ml tubes (Falcon) with the side with cells facing up, and 200 µl PBS was added to cover the top. The surface of the filters were then scrapped gently with a cell-culture scraper (with 1.3 cm flexible blade, TPP) for 30 s. The PBS solution with cells was then pipetted to a clean tube, and stored on ice and in the dark before cell sorting via FACS. To check hybridisations, parallel samples that were not scrapped-off filters were performed and stained with 4,6-diamidino-2-phenylindole (DAPI), and visualised with an inverted Leica TCS SP8X CLSM using appropriate excitation/emission settings for DAPI and the Oregon-Green 488-labeled tyramides.
For cell sorting, cells from CARD-FISH were resuspended with 1.8 ml of PBS, gently filtered through a 35 µm cell strainer (Corning), and sorted in 'purity-mode' using a BD FACSMelody™ Cell Sorter (BD Biosciences). Hybridised cells were detected with green-fluorescence (using manufacturer's' 'FITC' settings) and forward scatter, and sorting gates were placed higher than background fluorescence determined from NONEUB controls that were measured prior. Approximately 5000-7000 cells were sorted directly to polycarbonate filters (0.2 µm pore size, hydrophilic polycarbonate membrane, 47 mm diameter, Millipore), pre-coated with AuPd thin films (nominal thickness of 120 nm, obtained by sputter-deposition), which were placed on microscope cover-slips and on the 2 ml sort-tube holder. The flow rate for sorting was slowed to around 500 events per second, so that excess fluid did not build-up and spread over the filters, thereby ensuring that cells would be sorted to, and dry on, a small area of the filter.

Instrument

BD FACSMelody™ Cell Sorter (BD Biosciences)

Software

BD FACSChorus.

Cell population abundance

The FACS work in this study was not used for quantitative purposes.

Gating strategy

'Background' fluorescence of cells and residual particles from sediments was obtained by first running cells/samples hybridised by CARD-FISH with the NONEUB-HRP probe (and performing all steps as performed in standard CARD-FISH for specific probe hybridisations) through the FACS machine and recording 200,000 events 3 times. This was followed by running cells/samples hybridised by CARD-FISH with the specific probe Fusi-6-HRP through the FACS machine and recording 200,000 events 3 times, then setting the gate above the fluorescent signals obtained from the negative control, but that captured fluorescent signals of the specific probe-conferred events.

☒ Tick this box to confirm that a figure exemplifying the gating strategy is provided in the Supplementary Information.
